# Global Analysis of Polyfluorene via AB-Type Suzuki–Miyaura Polymerization: Empirical and Mechanistic Rationalization of Structural and Reaction Parameters on Molar Mass, Dispersity, and Yield

**DOI:** 10.1021/acspolymersau.5c00121

**Published:** 2025-12-22

**Authors:** Alexander Kleine, Ulrich S. Schubert, Michael Jäger

**Affiliations:** † Institute of Organic and Macromolecular Chemistry (IOMC), 9378 Friedrich Schiller University Jena, Jena 07743, Germany; ‡ Center for Energy and Environmental Chemistry Jena (CEEC Jena), Friedrich Schiller University Jena, Jena 07743, Germany

**Keywords:** chain-growth, step-growth, controlled polymerization, palladium-catalyzed, ring-walking efficiency, catalyst dissociation, effective SCTP length

## Abstract

The preparation of conjugated polymers with precisely tunable optoelectronic properties is based on modern polymerization techniques. In addition to conventional step-growth polycondensation for the underlying C–C coupling steps, the Suzuki-Miyaura catalyst-transfer polymerization (SCTP) continues to attract great interest, as it enables chain growth with controlled molar masses, dispersity values, and end group fidelity. The combination of these beneficial properties allows for use in defined block copolymers or as functional building blocks in functional polymer architectures aiming to control energy and charge transfer at the macromolecular level. For an ideal SCTP mechanism, a ring-walking step is crucial—and many experimental parameters contribute to the overall success of the polymerization to tailor the desired degree of polymerization, low dispersity values, and high yields. Many reports explored specific combinations within the available parameter space, yet a unifying analysis to identify limitations as well as promising future directions to advance synthetic methodologies is needed. In this work, all available experimental data for the AB-type 2,7-linked poly­(fluorene) is collected, and a detailed algorithmic global analysis is presented, including the tabulated results for machine learning and reinspection of the collected raw data. It is found that SCTP is typically obeyed for monomer-to-catalyst ratios up to 25, while the combination of ^*t*^Bu_3_P-based Pd precatalysts and boronate-based monomers enables excellent SCTP fidelity with monomer-to-catalyst ratios up to 500.

## Graphical abstract

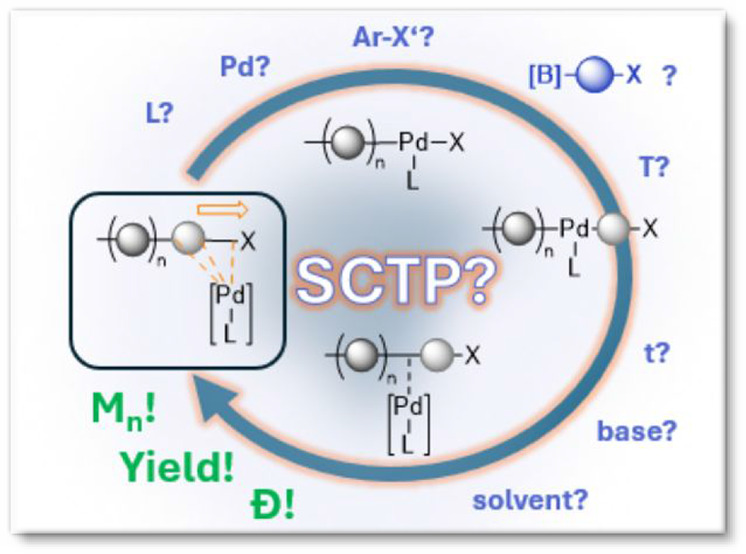

## 1 Introduction

The progress to prepare modern conjugated polymers (CPs)^1–4^ and their ability to facilitate charge- and energy-transfer processes as well as the conversion of light and electrical energy and *vice versa* enables their broad application as (opto-)­electronic active materials in organic solar cells, organic light-emitting diodes, organic field-effect transistors, or electric semiconductors, as well as photocatalytic components. The enormous application potential of CPs descends from the precise adjustability of the intrinsic photophysical and redox-chemical properties, which are primarily dictated by the chemical structure and substitution pattern of the repeating units along the chain. In addition, the side chain group (R) can be used to modulate spatial interchain interactions (stacking) as well as to tune the solubility, processability, and ultimately the morphological stability of the polymer domains in the device. Besides the structural parameters, conjugated polymers are further characterized by the average number of repeating units (*n*) and, related to this, the number-averaged molar mass (*M*_n_), as well as the dispersity (*Đ*). In addition, the role of the precise α- and/or ω-end groups of the chains has gained recent interest, e.g., to scrutinize the impact as trap sites for charge and energy transfer processes. Notably, the chain ends can be exploited to design and to couple functional (macro)­molecular units, e.g., to prepare conjugated block copolymers to warrant for directional transfer processes, for enhanced phase separation, etc.^5^ Hence, the rational design of such materials is tied to the ability to prepare CPs with great precision.^6^

The synthesis methods for the family of CPs have been reviewed extensively,^1–3,5,7–19^ providing great detail on (i) the employed repeating units (type of the repeating unit, the side group(s) R, or the complementary A and B functionality which set the C–C cross-coupling methodology), (ii) the active catalytic system that is formed from a metal precatalyst, ligands, and the activation mode, and (iii) general reaction conditions (reaction temperature, reaction time, molarity, solvent, base). However, the complex interplay of these influencing parameters makes it impossible to investigate all combinations in a systematic fashion. Hence, reviews in the field focus on the findings for each CP family, leading to invaluable but specific insights.

In this regard, the initiative toward “Findable, Accessible, Interoperable, and Re-usable (FAIR) data”^20^ holds great potential to derive a more comprehensive analysis of the individual reports, as well as to analyze the data in a quantitative manner, and to identify the main parameters. Hence, any existing robust data set forms the basis to scrutinize the inherent fundamental challenges, as delineated in the following. 2,7-Fluorenediyl monomers bearing complementary A/B functional groups for the Suzuki–Miyaura cross-coupling reaction provide a well-documented basis for analysis. With this goal, poly­(2,7-fluorene)­s represent an ideal substance class that benefits from consistent characterization by analytical size exclusion chromatography (SEC, mostly vs PS calibration) to provide comparable *M*_n_ and *Đ* data, whereas the chemical yield may be taken with caution in the case of substance loss upon purification. More appeal toward the progress of Suzuki-Miyaura catalyst-transfer polymerization (SCTP) originates from the identification of inherent limitations, which is related to the efficiency of the ring-walking step (*vide infra*) and, thus, the distance of Pd migration in the SCTP cycle—much longer than for thiophenes.

In addition, modern high-resolution mass spectrometry (HR-MS) is occasionally performed to gain further insights into the end group distribution, which is invaluable in combination with *M*_n_, *Đ*, and yield data to draw conclusions from the polymerization process itself.

Scheme 1 depicts the parameters and information that can be grouped into structural parameter classes (shown in blue) of the monomer (type of the boronic acid derivative, the halide, and the side chain group), the catalytic system (composed of or formed from different metal sources, ligands, stoichiometry, and the monoaryl halide that forms the α-terminus upon initiation), the reaction parameters (given by the solvent, base, reaction time, temperature, and concentration), and the resulting polymer characteristics (molar mass, dispersity, and yield, shown in green).

**Scheme 1:**
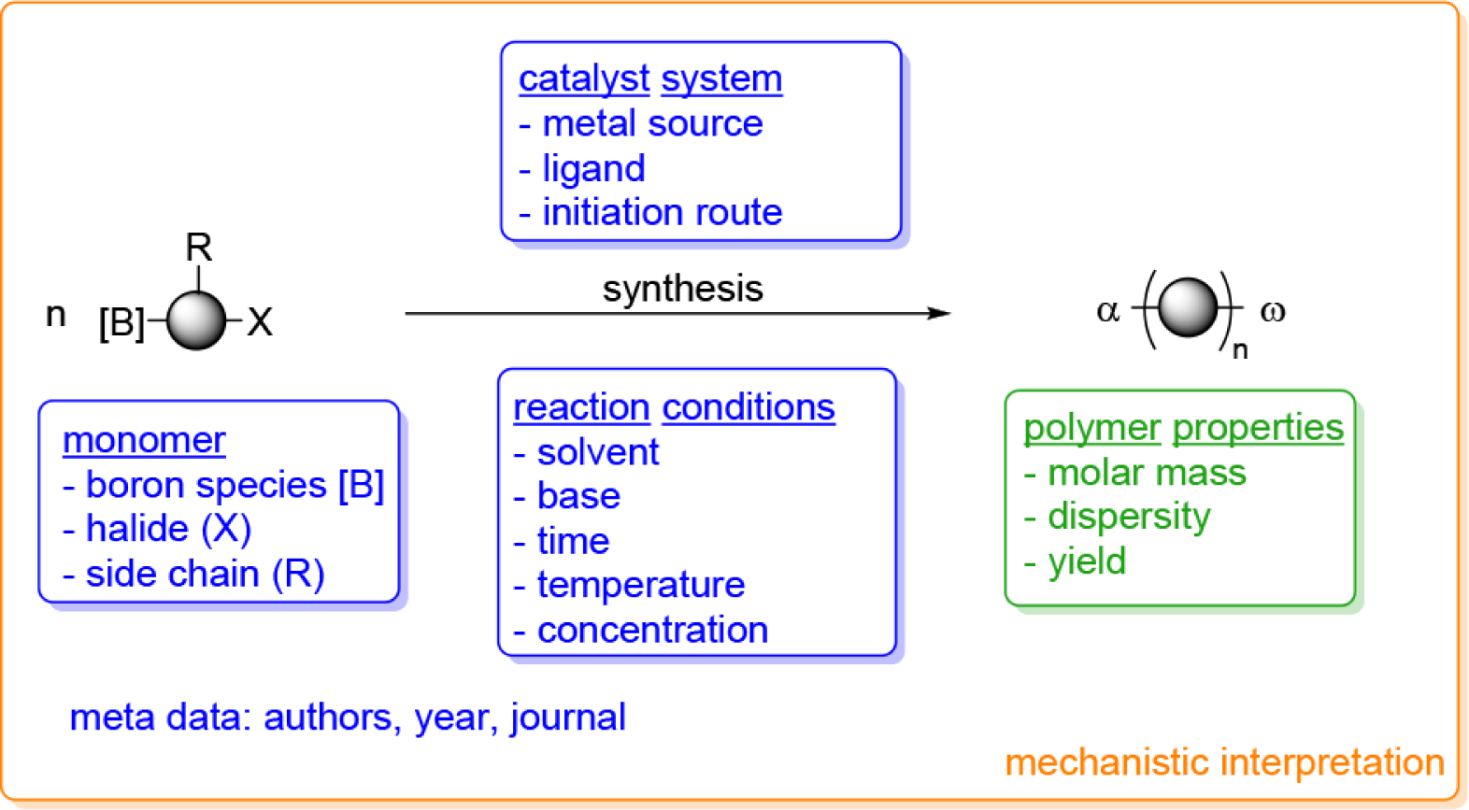
Schematic Representation of the AB-Type Suzuki Polymerization According to Influencing Parameters and Resulting Parameters Used in the Global Mechanistic Interpretation^a^

a Influencing parameters shown in blue classified as monomer, catalyst system, and reaction conditions, resulting in polymer properties (green) of the obtained polymer.

In this work, we present a systematic quantitative global analysis aiming at a comprehensive interpretation in terms of the catalytic (side) reactions. The data set was compiled manually from the literature reports on AB-type 2,7-fluorene monomers for the Suzuki–Miyaura cross-coupling reaction, i.e., from 36 publications with a total of 489 polymerization experiments. Notably, the experimental data was augmented with meta information taken from Supporting Information or the narrative experimental part. The tabulated electronic data set is provided in Supporting Information, to meet with the FAIR data principle and to assist further analysis, e.g., by *machine learning* and/or data mining, as well as *language-based models* and *chemical interpretation AI platforms*. The data table contains further bibliographic information (e.g., first author, corresponding author(s), publication year), to extract systematic subsets from the same group or historic developments (Figure 1).

**Figure 1:**
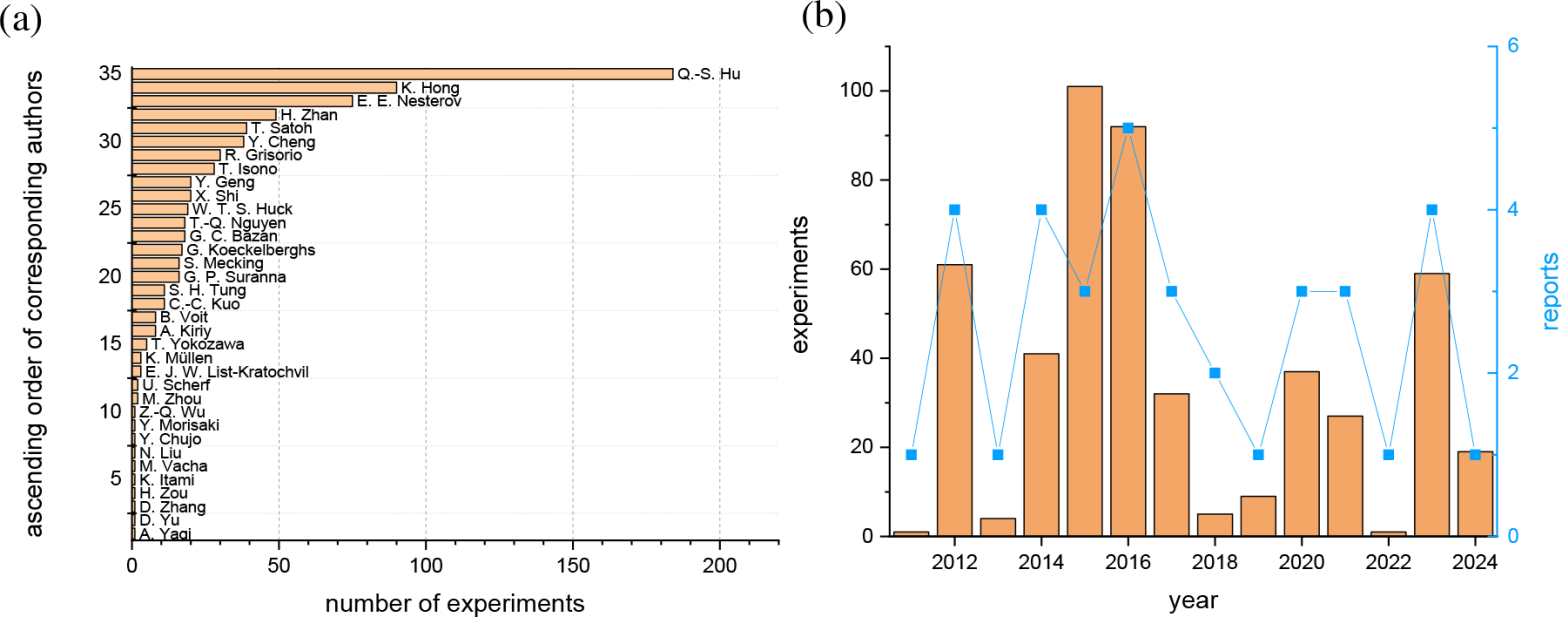
Meta data of the reported experiments (489) by work group (a) and by year (b) including the number of reports by year (blue, right axis).

## 2 Background of the Suzuki–Miyaura Polymerization

In general, Suzuki–Miyaura polymerizations/polycondensations are based on bifunctional substrates, bearing a complementary (pseudo)­halide and or boron derivative which will be denoted as A and B, respectively. These orthogonal functionalities can be utilized in an AA/BB-type approach, in which equal amounts of the AA and BB monomers are used, or in an AB-type polymerization using an asymmetric AB monomer. In view of the task to perform a global survey toward well-defined poly­(2,7-fluorenes) including defined end groups, we deliberately limit the discussion to the AB-type approach and an empirical analysis of the parameters.

In the following, the essentials of Suzuki–Miyaura cross-coupling with detailed explanation in Supporting Information will be introduced; i.e., the implications for the polymerization via a chain-growth or a step-growth mechanism will be derived. In order to assign respective contributions, the experimentally reported molar masses, dispersity values, and yields will be evaluated to reveal the interplay of both idealized mechanisms. This approach requires a robust categorization to assign the dominant mechanism, which is essential for meaningful analysis. In this regard, poly­(fluorene)­s appear to be ideal candidates owing to the rich set of reported data and the inherent mechanistic challenges (*vide infra*).

### 2.1 Overview of Suzuki–Miyaura Cross-Coupling for Polymerization

The Suzuki–Miyaura cross-coupling reaction proceeds via a Pd^0^-catalyzed cycle involving three main steps: oxidative addition (OA) of an aryl halide to Pd^0^, transmetalation (TM) with an aryl boron derivative, and reductive elimination (RE) to form the biaryl product and regenerate the catalyst (see Section S1.1 for details and reaction scheme).^21,22^ The reaction typically requires a mixture of aqueous and organic solvents, inorganic bases (often generating hydroxide ions *in situ*), and Pd catalysts. In polymer synthesis, AB-type monomers are used, because the polymer chains can be selectively initiated by monofunctional aryl halides to gain control of the α-terminus. In practice, the polymer initiation (see Section S1.2 for details and reaction scheme) can be achieved *in situ* using Pd^II^ salts at the expense of sacrificial homocoupling of boronic acid derivatives, releasing active Pd^0^ and forming AA-terminated byproducts. However, to retain AB-type fidelity, more controlled approaches utilize preformed Pd^0^ sources (e.g., Pd­(dba)_2_) or Buchwald-type precatalysts, which undergo a well-defined reductive elimination to generate active Pd^0^ without competing ligand interference. Next, the polymerization is typically assumed to follow a chain-growth mechanism (see Section S1.3), termed Suzuki–Miyaura catalyst-transfer polymerization (SCTP), if the Pd catalyst remains associated with the polymer chain. This implies that after each RE step, the Pd species migrates via π-coordination (“ring-walking”) to the new chain end, enabling subsequent OA and monomer addition. This mechanism leads to well-defined polymer molecular weight and dispersity values (*Đ* ≤ 1.20) that can be tuned by the monomer-to-catalyst ratio and conversion. The major deviations from the idealized ring walking result in catalyst dissociation and, thus, the initiation of new chains by OA into the monomer. Hence, the resulting boronic acid derivative terminus enables step-growth by chain–chain couplings (see Section S1.4 for details and reaction scheme), leading to increased molecular weight and higher dispersity values. Notably, chain–chain couplings release the active Pd^0^ catalyst, which amplifies this effect, and thus, high polymer yields with unexpectedly high molecular weight and broad dispersity reveal a significant step-growth contribution. One strategy to mitigate catalyst dissociation is based on the usage of excess monoaryl halide (Ar–X), in particular, if the rate of OA is higher than that of the excessive monomer. Another recent strategy aims to reduce the rate of OA of the monomer.^23^ Finally, ω-end group functionalization can be achieved upon addition of an excess of monoboronic acid derivative after the polymerization stage, which is highly attractive for the preparation of heterotelechelic polymers or precise block copolymer formation.

Nonetheless, the SCTP fidelity is determined by the propensity to ring-walking.^24^ If its efficiency is below unity, the step-growth effect will accumulate during the polymerization and, thus, is expected at a larger monomer-to-catalyst ratio or under excessively forcing reaction conditions. Hence, we selected poly­(fluorene) as an important and well-studied polymer, which features a “long” ring-walking distance compared to paradigmatic poly­(thiophene)­s.

### 2.2 Generalized Polymerization Scheme

Scheme 2 summarizes the interplay of the most important steps beyond the SCTP cycle, which will serve to rationalize the formed polymer characteristics (*M*_n_, *Đ*, and yield). In most studies exploiting AB-type monomers, an active catalyst fragment [Pd­(L)] is converted with a monofunctional aryl halide (Scheme 2, step i) in an initiation step to form “Ar–Pd­(L)–Br”. Afterward, the bifunctional monomer is added to enter the SCTP cycle, i.e., undergoing transmetalation (TM), reductive elimination (RE), ring-walking (RW), and oxidative addition to the intermediate ω-terminus to extend the polymer chain by one unit (*n*_new_ = *n* + 1).

**Scheme 2:**
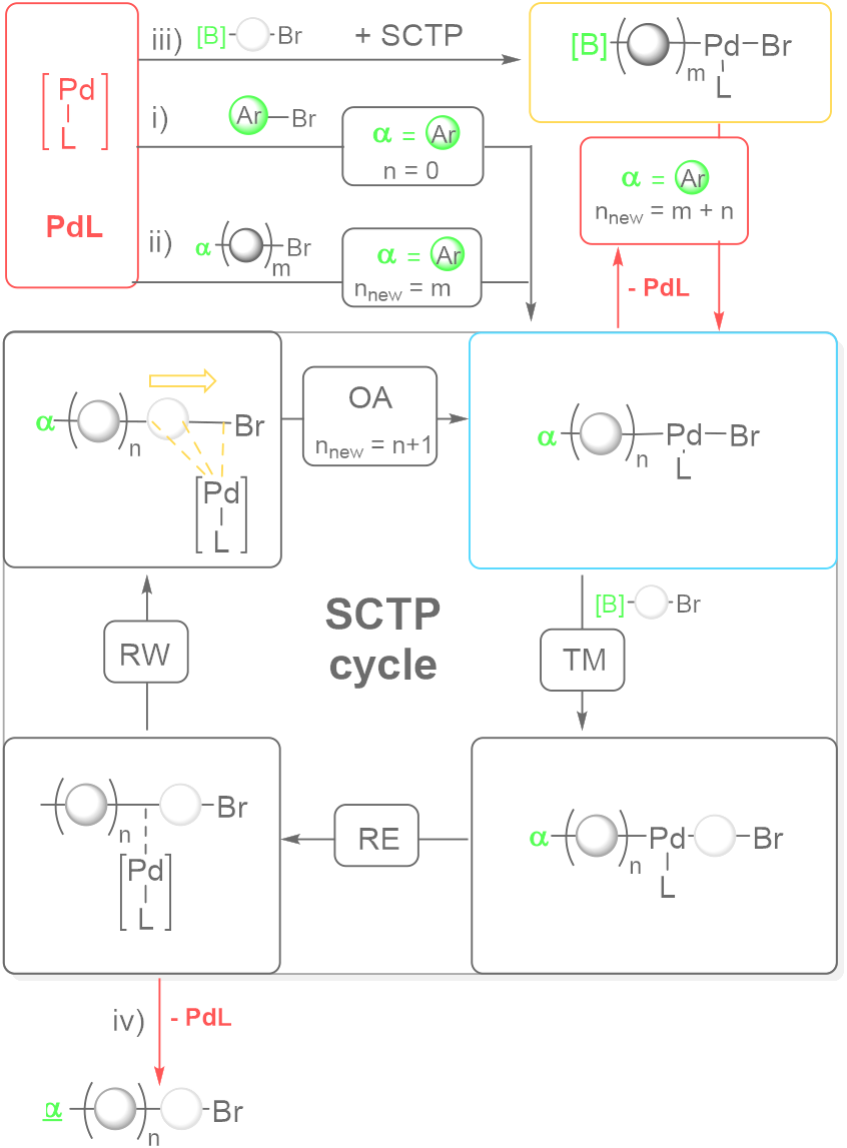
Schematic Representation of the Extended Catalytic Cycle of the Polymerization of AB-Type Monomers^a^

a The scheme is composed of initiation reactions (red box) from free active Pd­(L) with (i) monoaryl halide or restarting a polymer chain (ii) with the correct α-terminus, as well as with (iii) monomer. Steps (i) and (ii) yield the active polymer-Pd species (blue box) to follow the SCTP cycle by transmetalation (TM), reductive eliminiation (RE), ring-walking (RW), and oxidative addition to the ω-terminus to extend the polymer chain by one unit (*n*_new_ = n + 1). Contrary to ring-walking to maintain the SCTP cycle, catalyst dissociation releases a polymer and a free active Pd­(L) fragment (iv), prone to new chain initiation with monomer (and SCTP cycles) to form a boron-α-terminus (orange box). Subsequent reactions include the correct α-decoration and chain couplings upon reaction with an active polymer-Pd species. Note the inevitable release of free active Pd­(L) catalyst.

However, in the case of competing catalyst dissociation after reductive elimination, a free catalyst fragment [Pd­(L)] is formed. As will be discussed later, a surplus monofunctional aryl halide can be used in the first step, so [Pd­(L)] can reinitiate new chains (Scheme 2, step i) and reinsert into the same or a different polymer chain (Scheme 2, step ii). Notably, the catalytic cycle is (re)­entered and a correct α-terminus will be obtained. If reinitiation dominates, a lower *M*_n_ but narrow *Đ* is expected with respect to the theoretical value derived from the monomer-to-catalyst ratio (M/[Pd]) and the conversion. If reinsertion dominates, no significant deviations in *M*_n_ and *Đ* will be expected, as the mechanism resembles that of the reversible addition/fragmentation transfer (RAFT) mechanism.

More likely, [Pd­(L)] may also react with the monomer to initiate and to grow new chains with a reactive boronic acid (ester) α-terminus [B] (Scheme 2, step iii). Consequently, the “[B]–(fluorene)_n_–Pd­(L)–Br” species can undergo transmetalation reactions at the α-chain end. The first possibility involves the coupling between the α-terminus of “[B]–(fluorene)_n_–Pd­(L)–Br” and the ω-terminus of the “Ar–(fluorene)_m_–Pd­(L)–Br” that is present in Scheme 2. Hence, the chain coupling would lead to larger *M*_n_ and higher *Đ* values resembling step-growth behavior. The second possibility—if surplus monofunctional aryl halide is present to regenerate sizable amounts of “Ar–Pd­(L)–Br”—leads to a simple decoration of the correct Ar group at the α-terminus. Nonetheless, as [Pd­(L)] will be regenerated, the contribution of these reactions will accumulate during the progress of the reaction.

### 2.3 Categorization by Molar Mass, Dispersity, and Yield

It is important to realize that any deviation from the initial SCTP stage starts upon catalyst dissociation and that the additional pathways will accumulate and contribute to the formed ensemble of polymers. Hence, the polymerization results are grouped into four categories (Table 1) by dispersity and yield, which are indicative of the (dominating) growth mechanism. This assignment is plausible, considering that a chain-growth mechanism leads to low dispersity values and molar masses that obey the conversion and monomer-to-catalyst ratio (category “I”), while a step-growth mechanism is commonly expected to lead to broad distributions and molar masses that are dictated by coupling in the very late stage of coupling toward unity (category “IV”).

**Table 1:**
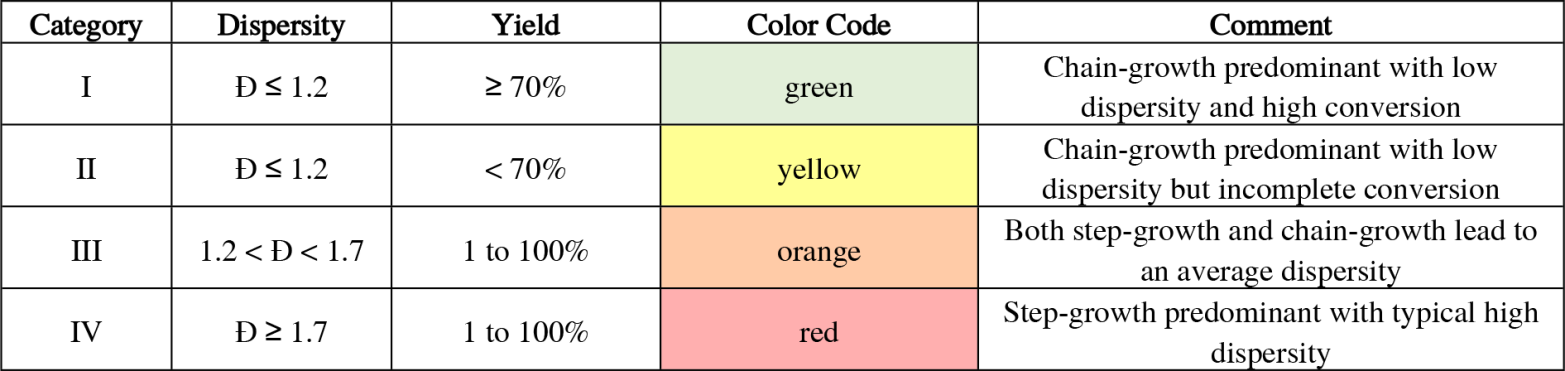
Categorization of the Results by Dispersity and Yield

In case of intermediate dispersity values that indicate an interplay of mechanisms, we included category “III” (1.2 < *Đ* < 1.7, shown in orange). In addition, low yields also imply limited clear assignments, which justifies category “II”. These categorizations will serve to highlight differences between step-growth polymerization (“IV”) and a controlled chain-growth polymerization commonly referred to as the Suzuki–Miyaura catalyst-transfer polymerization (“I”), as well as the interplay (“III”) and limited interpretability (“II”).

### 2.4 Application-Oriented Work

The utilization of poly­(fluorene) has been widely demonstrated for advanced architectures, e.g., polymer-analogous modifications, macroinitiators, and graft polymers (see Supporting Information for detailed information and tabulated data).^25–41^ These polymerizations were not investigated in detail and predominantly exhibited moderately broad molecular weight distributions (categories I–IV), and the SEC data indicate both catalyst-transfer and step-growth polymerization mechanisms (*vide infra*). These studies apply specific conditions, which prevent their meaningful comparison but will serve to evaluate the results from the systematic studies (*vide infra*).

## 3 Methodological Approach

The previous section provides the background of Suzuki–Miyaura AB-type polycondensations. In this section, we embark on systematically analyzing a set of potential influencing parameters on the reported polymer parameters. If available, we compare the data from systematic studies among each other, followed by the analysis of the refined data set. In other words, the conclusions drawn from specific self-consistent studies were checked against a generalization to derive a unifying understanding as well as to identify potential discrepancies.

### 3.1 Parameter Value Collection

For this task, the SciFinder Database was searched for fluorene equipped with either chloride, bromide, or iodide in the 2-position and a BO_2_ fragment in the 7-position to mimic halides and boronic acid derivatives. The substances found were limited to the substance class “polymer”, and all references were collected. These references were then manually screened for the performed *homo*-polymerizations yielding poly­(fluorene)­s, and every polymerization experiment was included in this data set. Most of our data were manually extracted and composed of tables, text, and graphics found in the manuscripts and/or Supporting Information. It should be noted that progress in automated data extraction is expected to perform this demanding task in the future reliably. In particular, information such as reagents, stoichiometric ratios, reaction conditions, etc., is often not considered for discussion. Notably, all obtained polymer parameters originate from analytical size exclusion chromatography data (SEC) using PS calibration or multiangle laser light scattering (MALLS) in rare cases. Hence, the molar masses and dispersity values can be compared reliably and justify the categorization introduced in Section 2.3.

### 3.2 Parameter Value Calculation

The original data set is augmented by important calculated information for a unifying analysis, e.g., equivalents of reagents and stoichiometric ratios, concentrations, or the interconversion of molar mass and degree of polymerization. For this task, the degree of polymerization (DP) is useful to compare the obtained and expected molar masses for poly­(fluorene)­s irrespective of the different side groups R. DP can be derived from the reported molar mass (based on PS calibration) by division by the molar mass of the repeating unit to account for the different sizes of the side groups. It should be noted that for even more accurate treatment, the occupied molecular volume should be used, which is beyond the scope of this study. Because the majority of experiments differ only slightly in the side chain by two methylene groups, we adhere to the reported molar masses (*M*_n_) for the sake of simplicity. Assuming ideal SCTP behavior, the theoretical degree of polymerization (DP^theo^) is given by the number of monomer units per catalyst unit (monomer-to-catalyst ratio). In the limit of complete conversion, the ratio of *M*_n_/(monomer-to-catalyst ratio) should result in a constant value, which represents the molar mass per repeating unit based on PS calibration, because the experimental *M*_n_ values descend from SEC data. In the case of ideal SCTP behavior but incomplete conversion, the same value for molar mass per repeating unit should be obtained by scaling with the conversion—assuming that the conversion is adequately expressed by the chemical yield; e.g., product losses upon purification are not considered. Hence, a universal value for the ratio *M*_n_/((monomer-to-catalyst ratio)/yield) is indicative of SCTP fidelity.

### 3.3 Overview of Categorization

The categorization was introduced in Section 2.3 primarily to assign the dominant polymerization mechanism. For this task, the dispersity values help to distinguish chain-growth behavior (low dispersity) from step-growth behavior (high dispersity). The yield reflects the progress of the polymerization reaction toward completion and, thus, may provide information on what stage a departure from the SCTP behavior becomes sizable.

Figure 2 illustrates the four categories for each reported experiment (dots). Each category is represented by the center of gravity (CoG) of its datapoints to “condense” the scattered data and to easily assess the qualitative expectations from the categorization. Figure 3 displays the *M*_n_ values as a function of dispersity (*Đ*) and chemical yield according to the categorization (I to IV, see Table 1): A high dispersity (*Đ* ≥ 1.7, category IV, shown in red) correlates with a CoG at high molar masses (Figure 3a) and intermediate yield (62%, Figure 3b). A low dispersity (*Đ* ≤ 1.2, category I and II, shown in green and yellow) correlates with CoGs at lower molar masses (Figure 3a) and lower yields (II at 42% vs I at 82%, Figure 3b), respectively. As expected, intermediate dispersity values (1.2 < *Đ* < 1.7, category III, shown in orange) were reported for a broad intermediate region of molar masses (Figure 3a) and yields (Figure 3b).

**Figure 2:**
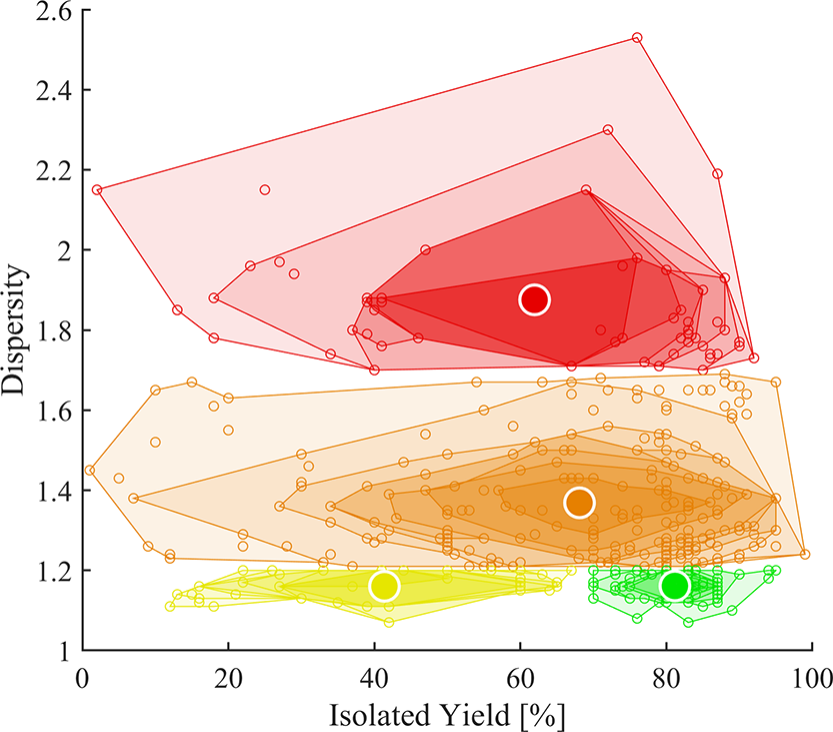
Categorization of all polymerization results according to their yield and dispersity *Đ* (see Table 1 for details). Center-of-gravity (CoG) of each category is represented by bold circles.

**Figure 3:**
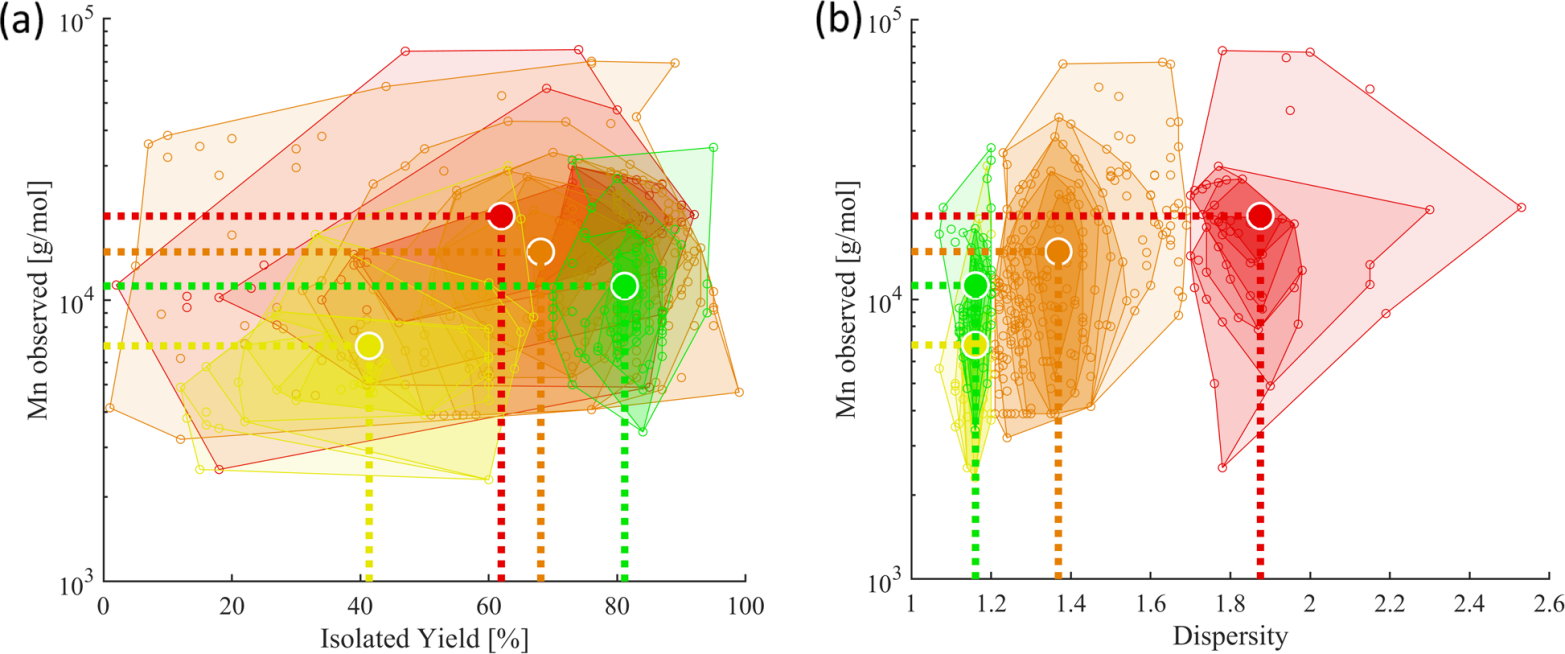
Overview of the obtained molar masses (*M*_n_) vs dispersity (a) and vs yield (b) illustrating the order of CoGs of *M*_n_ (category IV, in red) > *M*_n_ (category III, in orange) > *M*_n_ (category I, in green) > *M*_n_ (category II, in yellow). Center-of-gravity (CoG) of each category represented by bold circles.

### 3.4 Chain-Growth versus Step-Growth

A challenge for unifying analysis in the field of SCTP stems from the varying contributions of chain-growth and step-growth among the studies. In addition to the dispersity values, the observed molar masses and the chemical yields can be used to identify the transition from chain-growth behavior toward contributions of step-growth (see Section 3.2).

Many reports provide information to analyze the influence of the monomer-to-catalyst ratio. For most of these studies, a strict monotonic correlation of the monomer-to-catalyst ratio with the observed molar mass (*M*_n, exp_) and *Đ* is found. The corresponding data sets are displyed for monomer-to-catalyst ratios up to 50 and up to 500 to illustrate the two limiting regimes: **Below a monomer-to-catalyst ratio of 25**, a linear correlation with *M*_n, exp_ is observed (Figure 4a) that follows a unifying master curve. The averaging of datapoints leads to a slope of approximately 1.0 kg/mol per monomer-to-catalyst ratio unit, as calculated by Δ*M*_n_/Δ­(monomer-to-catalyst ratio) = 300 kg/mol/30 units. The majority of short-length polymers also feature low dispersity values (<1.3, Figure 4c), albeit a few polymers reveal unusually high dispersity values (*Đ* up to 1.8). Importantly, the linear correlation with *M*_n_ and low *Đ* clearly supports the assignment to an SCTP mechanism; i.e., the chain-growth mechanism is dominant. **Above a monomer-to-catalyst ratio of 25**, the correlation curves with *M*_n, exp_ become flatter (Figure 4a,b), and the corresponding dispersity values are typically higher (Figure 4c,d) except for the system studied by Kobayashi et al. (2020). This polymerization study is outstanding, as the linear correlation of the monomer-to-catalyst ratio with *M*_n_ was still maintained with low dispersity values above a monomer-to-catalyst ratio of 25.

The deviation from a linear correlation may also arise from incomplete conversion. The analysis of the corresponding reports (Dong et al. 2017, Zhang et al. 2015, and Ying et al. 2012) support this hypothesis: The nonmatching correlation reported by Dong et al. in 2017 (monomer-to-catalyst ratio = 50) can be explained by the low yield (27%) as compared to the yields at lower monomer-to-catalyst ratios (79 to 94%). For Zhang et al. 2015, the entire set of polymers was obtained in yields between 22 and 40%. The data by Ying et al. 2012 further support this assignment, while two datapoints (monomer-to-catalyst ratio = 13 and 20) reveal high dispersity values assigned to fluctuations in the polymerization procedure.

**Figure 4:**
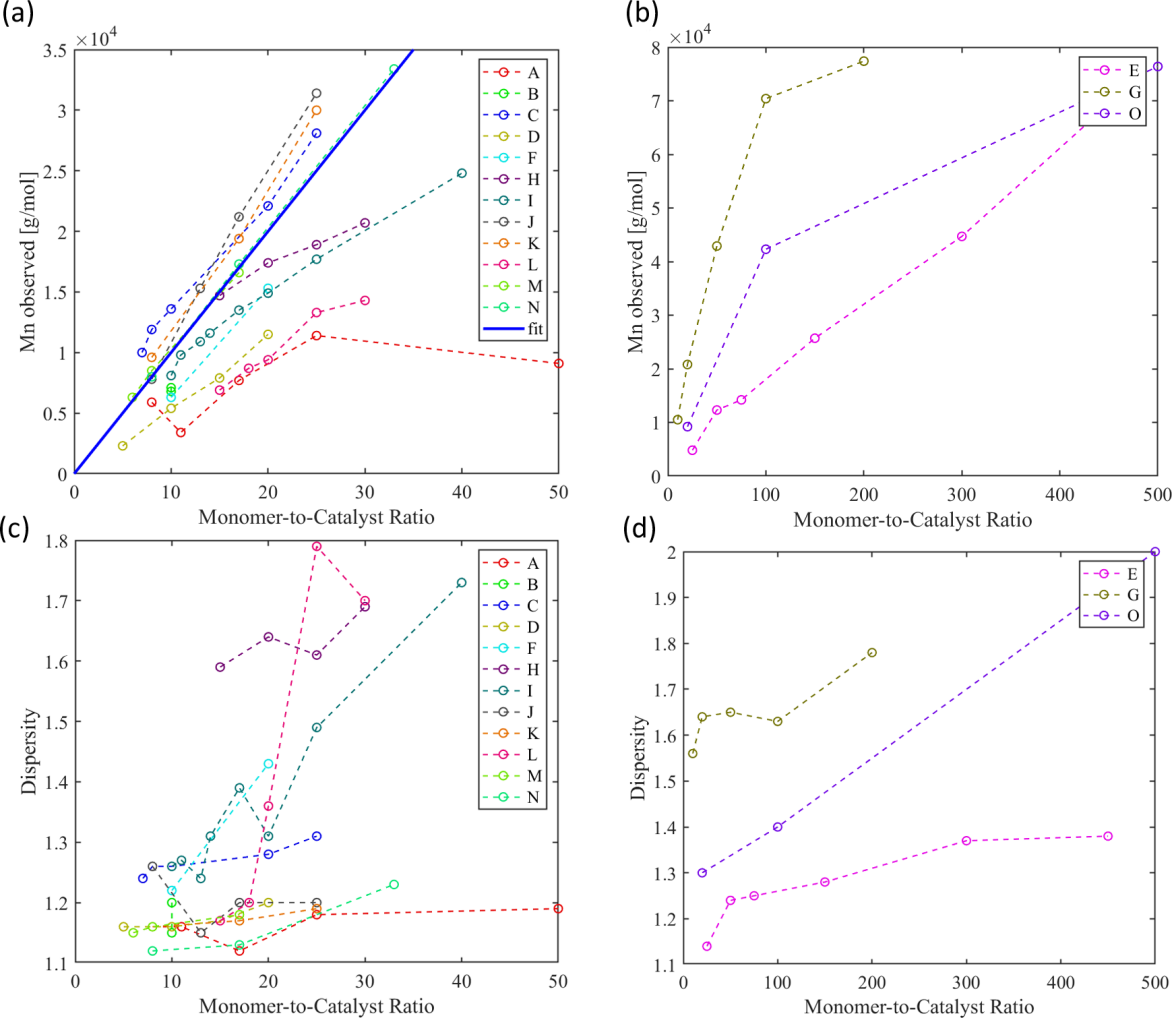
Correlation of monomer-to-catalyst ratio vs determined molar masses (a,b) and vs dispersity values (c,d) according to the reactions published in references testing up to a monomer-to-catalyst ratio = 50 (a,c) or in references including polymerizations above a monomer-to-catalyst ratio = 50 (c,d). The blue line in panel (a) represents the linear master curve with a slope of Δ*M*_n_/Δ­(monomer-to-catalyst ratio) of 1.0 kg/mol per monomer-to-catalyst ratio unit (see the text).

Next, the global data set of experiments was analyzed in terms of the categories by the center-of-gravity. Indeed, the category “I”—as defined by a low value for CoG_Đ_ and high value for CoG_Yield_— revealed an observed CoG_Mn_ of 11.2 kg/mol (Table 2, entry 1). In terms of the ideal SCTP mechanism, the expected molar mass is determined by the monomer-to-catalyst ratio and the monomer conversion. Hence, dividing the observed molar masses by the yield leads to a value that represents the projected molar mass upon full conversion. For example, a value for CoG_Mn/yield_ of 13.9 kg/mol is calculated for category “I”. Next, this scaled molar mass value is further scaled by the applied monomer-to-catalyst ratio, which should result in a constant value in case of ideal SCTP. For example, the ratio CoG_Mn/yield_/CoG_monomer‑to‑catalyst_ = 0.99 kg/mol was calculated for category “I”—in surprisingly good agreement with the slope of the master curve derived from systematic series (Figure 4a). Next, the category “II” represents incomplete conversion but low dispersity values (Table 2, entry 2). The calculated value for the CoG_Mn/yield_/CoG_monomer‑to‑catalyst ratio_ of 0.98 kg/mol per unit is again in excellent agreement with an SCTP mechanism. The analysis of category “III” (mixed mechanisms, Table 2, entry 3) provides a larger value of 1.18 kg/mol per unit, which means that more units were incorporated than expected for an ideal SCTP behavior. This is plausible in the case of the expected contribution of some chain–chain couplings in line with the broadened dispersity. In case of category “IV”, an even larger CoG_Mn/yield_/CoG_monomer‑to‑catalyst ratio_ value is expected. However, if chain–chain couplings occur to a larger extent, new chains are also started to a larger extent, which oppositely leads to a lowering of CoG_Mn/yield_/CoG_monomer‑to‑catalyst ratio_. Indeed, a value of 1.04 kg/mol per unit was calculated for category “IV” (Table 2, entry 4). The interpretation of the mean CoG values is further corroborated by the brief assessment of the distribution of datapoints, as illustrated by the shaded areas in the contour plots (Figure 5b). The variations increase in the order “I”, “II”, “III”, and “IV”. Despite the simplification, the calculated value of the CoG_Mn/yield_/CoG_monomer‑to‑catalyst ratio_ from experimental data can serve as a parameter to judge the SCTP fidelity.

**Table 2:**
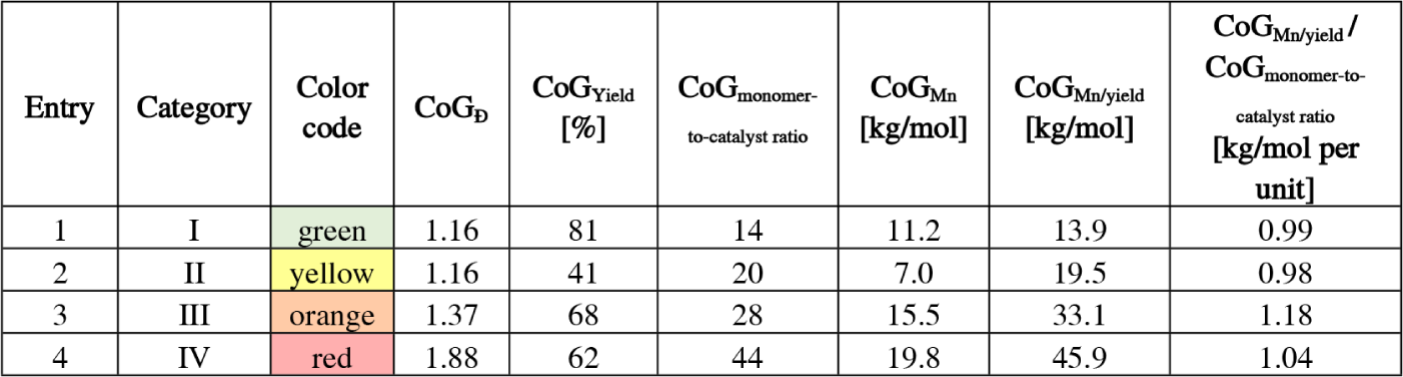
CoG Analysis of the Categories I–IV by Monomer-to-Catalyst Ratio vs Observed Molar Mass

**Figure 5:**
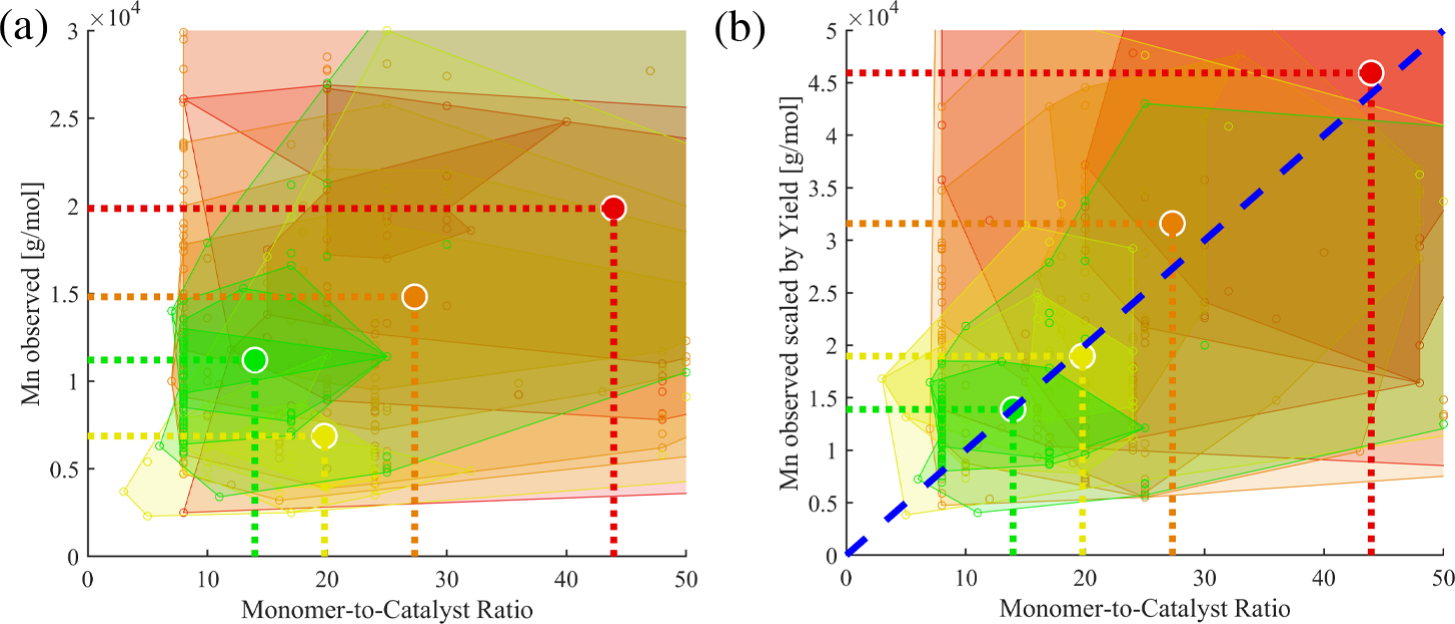
Correlation of (a) observed molar mass vs monomer-to-catalyst ratio and (b) molar mass scaled by yield vs monomer-to-catalyst ratio. The blue dashed line indicates the slope of 1.0 kg/mol per repeating unit (see text for details). Bold circles represent the center-of-gravity (CoG) of the categories I (green), II (yellow), III (orange), or IV (red).

### 3.5 Data Refinement by Monomer-to-Catalyst Ratio

We applied the findings for the effect of the monomer-to-catalyst ratio in view of the latter analysis in terms of SCTP (*vide infra*). Because low dispersity values were found (categories “I” and “II”) as well as the excellent correlation of *M*_n_, monomer-to-catalyst ratio, and yield was observed for values below 25, a dominant chain-growth mechanism is plausible in this regime. Hence, higher dispersity values for monomer-to-catalyst ratios ≤ 25 indicate a limiting role of one or many influencing parameters. Above a monomer-to-catalyst ratio of 25, lower *M*_n_ values than predicted were found consistently. This finding can be explained by the initiation of new chains, which will inevitably occur if catalyst dissociation sets in. Hence, the average molar masses decrease, although high yields are still obtained. In the meantime, Nesterov reported a similar effect termed “critical length”, which was assigned to approximately twelve fluorene-2,7-diyl units in case of a Br/B-MIDA-based monomer (MIDA is *N*-methyliminodiacetic acid).^23^ Hence, the analysis of further influencing parameters (*vide infra*) on the SCTP behavior with monomer-to-catalyst ratios > 25 should be taken with caution as the effect of the parameter of interest may be governed by the “critical length.” Hence, the global data set will be limited by the monomer-to-catalyst ratio ≤ 25 to result in a **refined data set** for interpretation in terms of a true SCTP mechanism.

### 3.6 Limiting vs Nonlimiting Parameter Effects

It is of high importance to realize that an apparent correlation of a parameter with the polymer categorization should be taken with caution and may be limited by another parameter. For example, Figure 6 illustrates that the two dominant types of side chains (*n*-hexyl and *n*-octyl) differ significantly in terms of categorization and, thus, the assigned dominant mechanism. Given the small difference of only two chemically *inert* distant −CH_2_– groups, this appears counterintuitive from a chemical perspective. Instead, it appears more plausible that an involved “catalyst parameter” or “reaction parameter”—which may differ markedly—causes the observed difference. In other words, the outcome of a polymerization reaction is influenced by the interplay of all parameters. Since a strict analysis of parameters requires that all other ones are kept identical or at least similar, an enormous number of experiments would be required. For example, five parameters with ten different values each would result in 100,000 experiments. Hence, a parameter should be regarded as not inherently limiting for SCTP if it leads to categories I and II. If different parameter values (e.g., the type of side chain) lead to diverse categorization but all other parameters are identical/comparable, a limiting effect might be assigned.

**Figure 6:**
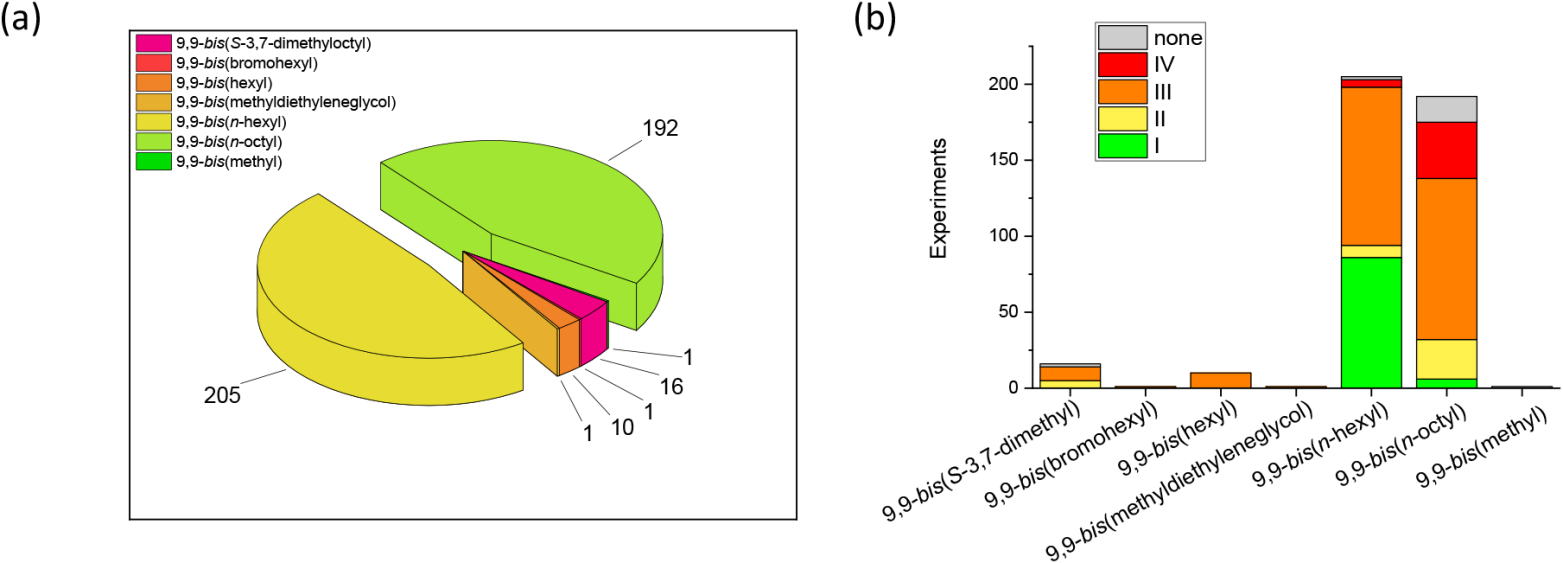
(a) Distribution of the monomer-related parameter “side chain”. (b) Correlation of the “side chain” with the categorization of the obtained polymer (see Table 1 for details). None denotes results with missing data (molar mass, dispersity, or yield) that are thus not in categories I to IV.

## 4 General Overview of AB-Type Polymerization

With the categorization in hand, this section provides a first overview of studies on poly­(fluorene)­s. The first part covers reports that focus on the properties and applications of the synthesized polymers and is kept concise as only a few polymerization reactions were performed. The second part provides an overview of the studies that explored systematically selected parameters on the polymerization results.

### 4.1 “Application-Oriented” Experiments

This section describes the work that focused on the usage and incorporation of synthesized poly­(fluorene) into more complex architectures, e.g., in polymer-analogous reactions, as macroinitiators or onto graft polymers. Hence, the performed polymerization reactions were typically not analyzed in depth and yielded mostly moderately broad distributions, as indicated by the available data for categorization (I to IV). Some results indicate the proposed SCTP mechanism, while others are assigned to a step-growth polymerization based on the analytical SEC data.^25–41^

Elmalem et al. (2011) synthesized a poly­(fluorene) to compare the polymer to a push–pull-type polymer based on fluorene-2,7-diyl as the donor and benzothiadiazole as the acceptor unit (Table 3, entry 1).^25^ Next, both Elmalem et al. (2012) and Pevzner et al. investigated the properties of the synthesized polymers (Table 3, entries 2 to 3).^26,27^ Furthermore, Elmalem et al. (2012) synthesized heterotelechelic polymers with α- and ω-chain ends with varying electronic characteristics. The influence of the chosen aryl halide and end-functionalization reagents on the charge- and energy-transfer properties was investigated. Pevzner et al. used different fluorinated side chains on the fluorene chromophore, examining the obtained optoelectronic properties. The Mecking group performed syntheses of poly­(fluorene)­s on the surface of cadmium-based quantum dots yielding hybrid particles, using a *grafting to* approach or a *grafting from* approach (Table 3, entries 4 and 5).^28,29^ Various reports detail the synthesis of *block* copolymers. Fischer et al. synthesized *block* copolymers with polyethylene glycol in a *grafting to* approach to form nanoparticles (Table 3, entry 6),^30^ while Zhou et al. have chosen a *grafting from* approach with 2-(dimethylamino)­ethyl methacrylate to form micellar structures (Table 3, entry 7).^31^ Gutacker et al. investigated copolymers in a *grafting from* approach (Table 3, entry 8) but used fluorene-based monomers in both blocks that vary only in their side chains. By postpolymerization modification, a neutral-*block*-ionic copolymer was synthesized and analyzed according to its aggregation behavior.^32^ Steverlynck et al. synthesized *graft* copolymers with poly­(fluorene) in the side chain and poly­(thiophene) in the main chain in a *grafting to* approach (Table 3, entry 9).^33^ In a similar approach, Tsuji et al. grafted poly­(fluorene) to a xanthene-based polymer backbone forming self-assembling polymer-layered structures (Table 3, entry 10).^34^ Jiang et al. (2020) (Table 3, entry 11) used poly­(fluorene)­s for luminescent nanofibers by blending conjugated rod-coil block copolymers with an inorganic perovskite-based sensitizer.^35^ Later, Jiang et al. (2021) extended the concept to rod-coil block copolymers with biobased materials to explore stretchable touch-responsive light-emitting diodes (Table 3, entry 12).^36^ Tokita et al. (2021) reported a single polymerization that was further exploited in the study (Table 3, entry 13).^37^ In 2022, Fujiki et al. applied the SCTP methodology to hybridized poly­(fluorene)­s supported by a dendrimer followed by reductive cleavage (Table 3, entry 14).^38^ Zou et al. (2023) prepared helical assemblies featuring circularly polarized luminescence by combining a poly­(fluorene)-based block with poly­(phenyl isocyanide)­s (Table 3, entry 15).^39^ He et al. (2023) functionalized paper with poly­(fluorene)­s as fluorescent sensors for screening of cell carcinoma (Table 3, entry 16).^40^ Nakamura et al. (2023) reported on single-chain force microscopy and fluorescence spectroscopy to elucidate intrachain aggregation coupling phenomena (Table 3, entry 17).^41^ The latter examples of block and grafted copolymers elegantly demonstrate the continuous high interest in using poly­(fluorene)­s as light- and electro-responsive materials.

**Table 3:**
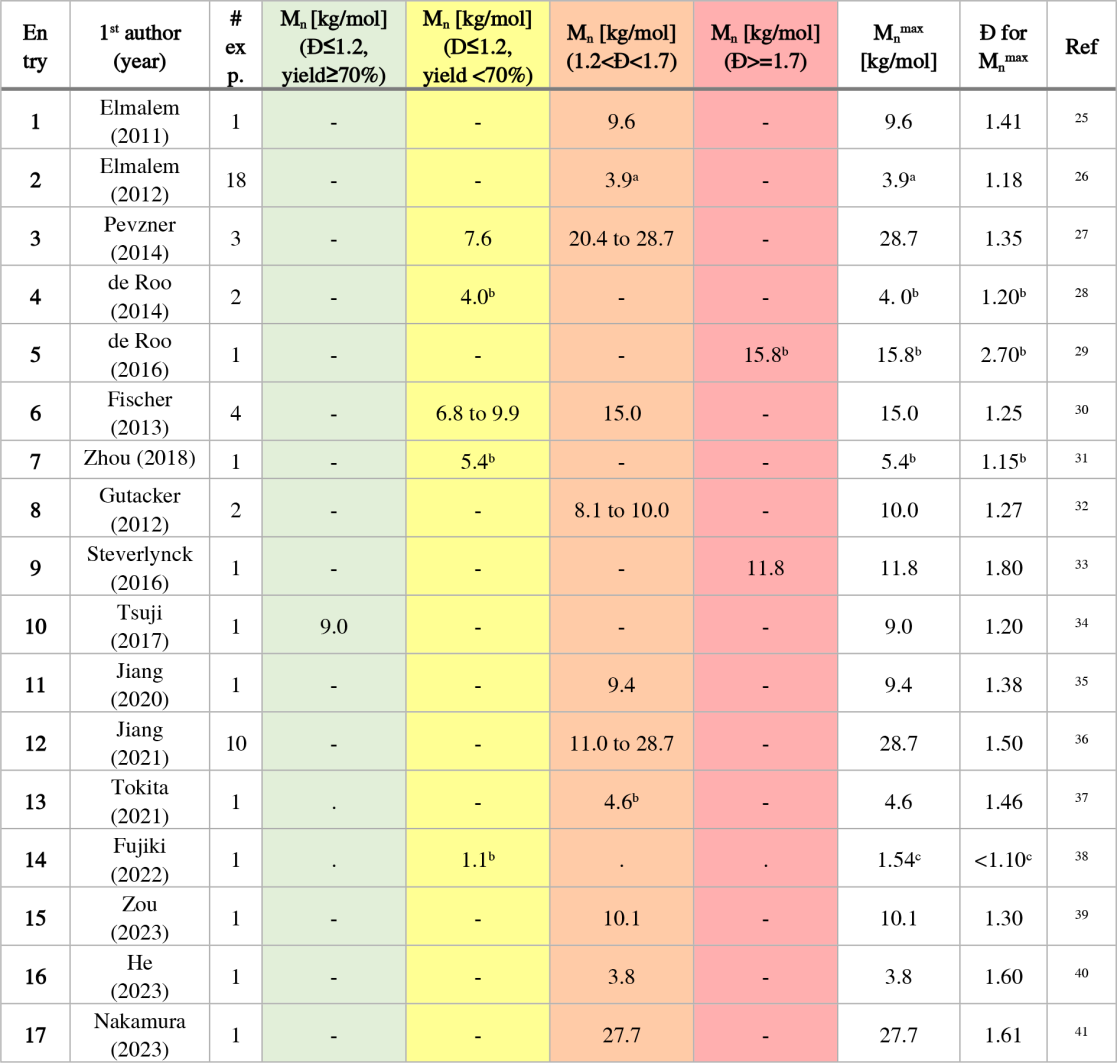
Application-Oriented Studies of Poly­(fluorenes) via AB-Type Suzuki Polycondensation

a *M*_n_ from averaged data by NMR spectroscopy.

b No yield was stated.

c Calculated from MALDI-MS data from Figure S11 in reference.^38^

### 4.2 Systematic Studies Addressing Selected Parameters

In the following, the approach and key findings of the systematic studies are introduced, including the polymerization conditions (Table S1). The main polymer’s characteristics in terms of molar mass range and dispersity values are provided in Table 4, complemented by the number of experiments per category (in parentheses). Four studies embarked from an inorganic Pd^2+^ salt (Table 4, entries 1 to 4, blue-shaded), which require the *in situ* activation step to generate the initial X–M_2_–Pd­(L)–X species. Fourteen studies report on initiating catalyst systems embarking from Ar–Pd­(L)–X’ (Table 4, entries 5 to 16) and varying specific parameters in a systematic fashion.

**Table 4:**
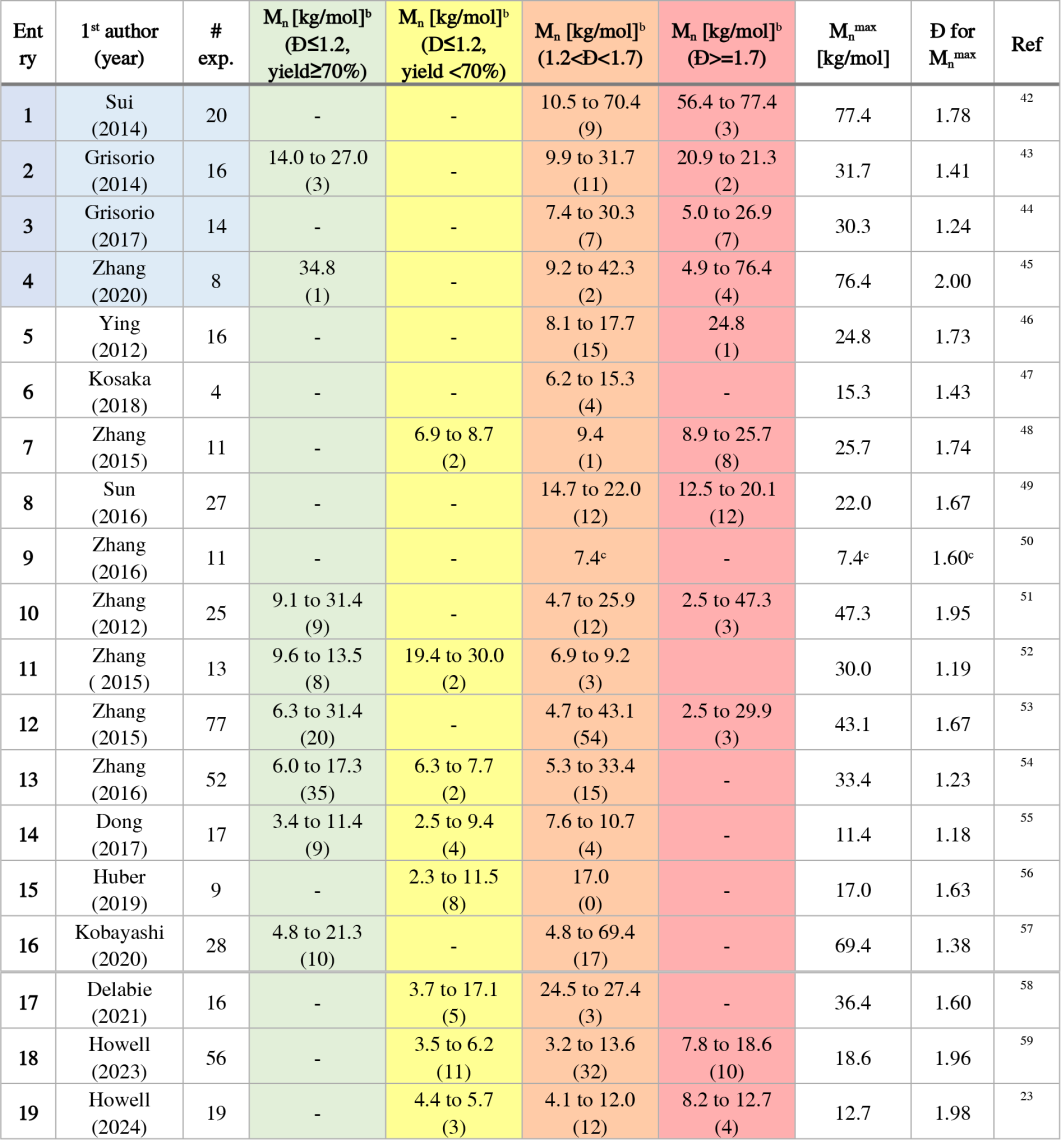
Overview of Optimization Studies on AB-Type Suzuki Polycondensation Reactions

a Blue-shaded: mainly Pd^II^ precatalysts. Color shadings of *M*_n_ indicate the categorization of results (see text for details).

b Number in parentheses represents the number of experiments per category. Note that the category “non-assigned” is omitted for brevity.

c Incomplete data (yields reported in the range from 81 to 88%, molar mass from MALDI analysis).

Sui et al. performed most of their 20 polymerization experiments using the carbene ligand “IPr” and palladium­(II) acetate as precatalyst and K_3_PO_4_ as base in aqueous THF at 20 °C for one hour (Table 4, entry 1).^42^ The authors compared the catalytic system to PEPPSI-IPr (IPr and 3-chloropyrid-1-yl coordinated to PdCl_2_) and to IPr with a separate *tris*(dibenzylideneacetone)­dipalladium­(0). In the study, different bases (K_3_PO_4_, K_2_CO_3_, Cs_2_CO_3_, CsF, LiOH, and Bu_4_NOH) were tested for a monomer-to-catalyst ratio ranging from ten to 200. The authors further examined different solvent mixtures, i.e., toluene/water, dioxane/water, and various THF/water ratios. Consequently, a wide range of molar masses was reached, ranging from 10 to 70 kg/mol.

The groups of Grisorio and Suranna covered Pd­(OAc)_2_-based catalysts (Table 4, entry 2)^43^ and later by Grisorio et al. the unusual sodium tetrachloropalladate in comparison to Pd­(PhCN)_2_Cl_2_ (Table 4, entry 3),^44^ employing tri­(*tert*-butyl)­phosphine (P­(^*t*^Bu)_3_) as the ligand or the PEPPSI-IPr system for a Br/Bpin-based monomer (bromide and boronic acid pinacol ester). The influences of the type of base (K_3_PO_4_, Na_2_CO_3_/K_2_CO_3_/Cs_2_CO_3_, and Et_4_NOH) and equivalents (10 or 20) were examined, as well as the mixing procedures of the palladium source, the base, and the ligand, the monomer-to-catalyst ratio (from 7 to 25), and experiments with additives (Bu_4_NCl/LiCl). The studies led to polymers with broad to narrow molar mass distributions ranging from 10 to 32 kg/mol.

Zhang et al. (2020) also used a Br/Bpin-based monomer and a Pd­(MeCN)_2_Cl_2_/P­(^*t*^Bu)_3_-based catalytic system (Table 4, entry 4).^45^ The performance of the catalytic system was compared to the widely used catalyst *tetrakis*-triphenylphosphine palladium(0) (Pd­(PPh_3_)_4_) and was evaluated for the bases sodium carbonate and tetraethylammonium hydroxide, temperatures between 20 and 110 °C, and reaction times from one to 24 h. The studied monomer-to-catalyst ratio varied from 20 to 500, and the solvent mixtures THF/H_2_O and toluene/H_2_O were compared. Importantly, the authors obtained molar masses of up to 76 kg/mol.

In the following, the reports that employ more defined conditions in terms of SCTP are summarized; i.e., the active catalysts were (externally) prepared from a Pd(0) source and a monofunctional aryl halide prior to the polymerization steps.

Ying et al. tested *ex situ* prepared Pd catalysts with phenyl bromide and P­(^*t*^Bu)_3_ to form Ph-Pd­(P­(^*t*^Bu)_3_)-Br for the polymerization of the octyl-decorated Br/Bpin monomer.^46^ By varying both the monomer-to-catalyst ratio (10 to 40) and the reaction times (0.5 to 60 minutes), polymers with molar masses ranging from 8 to 25 kg/mol were obtained, albeit the unusually high dispersity values indicate the occurrence of step-growth polymerization steps (Table 4, entry 5).

Kosaka et al. tested related *ex situ* prepared active Pd-catalysts, but with *o*Tol-Br as the aryl halide and AmPhos as the ligand, again with the octyl-decorated Br/Bpin-based monomer.^47^ The authors compared the catalysts to *in situ* prepared active catalysts from the aryl halide (4-iodobenzonitrile) and the Buchwald-type precatalyst AmPhos Pd G2, investigated the effect of different bases (CsF vs K_3_PO_4_), as well as different monomer-to-catalyst ratios (10 to 20). The synthesized polymers were comparable in molar mass (six to 15 kg/mol) with moderately narrow distributions, which is assigned to a combination of step- and chain-growth steps (Table 4, entry 6).

The groups of Zhang and Cheng again tested *ex situ* prepared active Pd-catalysts in three studies. Using either P­(Cy)_3_, P­(Xyl)_3_, or P­(*o*Tol)_3_ as ligands and a Br/BO_2_prop-based monomer equipped with octyl side chains, the groups tested various aryl halides (both bromides and iodides) based on triarylamine (Table 4, entry 7),^48^ perylene diimide (Table 4, entry 8),^49^ or functionalized benzenes (Table 4, entry 9).^50^ The studied temperature was varied (20 or 75 °C), and the monomer-to-catalyst ratio was between 15 and 30. The polymers revealed low to medium molar masses (7 to 26 kg/mol) with higher dispersity values indicative of mainly step-growth.

The group of Hu (and Hong) published a series of five reports, all based on the hexyl-decorated Br/Bpin monomer. In 2012, Zhang et al. used mainly *tris*(dibenzylideneacetone)­dipalladium mixed with tri­(*tert*-butyl)­phosphine and aryl halides to form an active Pd catalyst *in situ* (Table 4, entry 10).^51^ In this study, the catalyst system was compared to an *ex situ* prepared initiating catalyst (Ar-Pd­(P­(^*t*^Bu)_3_)-X) for which the aryl halide was varied (ArBr, ArI, or none), and different bases (K_3_PO_4_, K_2_CO_3_/Na_2_CO_3_, and K_2_HPO_4_) were compared at temperatures below room temperature (−10 to 20 °C). In 2015, Zhang et al. continued with the established catalyst system, now focusing on functionalized 4-phenyl halides bearing triflate (OTf), tosylate (OTs), or chloride (Cl) groups (Table 4, entry 11).^52^ The third study reevaluated the previously published results (Zhang et al. 2012) in comparison to additional parameter variations (Table 4, entry 12). The halide of the monomer (Br vs I) as well as the amount and type of aryl (pseudo)­halide (0.8 to 33 equivalents for Cl, up to 25 for OTf; various 4-*R*-phenyl halides) were screened.^53^ Next, Zhang et al. performed polymerizations using the Buchwald-type P­(^*t*^Bu)_3_ Pd G2 precatalytic system in combination with various aryl halides (Table 4, entry 13).^54^ Both Br/Bpin- and I/Bpin-based monomers were tested in addition to a range of bases (and base combinations: K_3_PO_4_, Na_2_CO_3_/K_2_CO_3_/Cs_2_CO_3_, and K_2_HPO_4_). The latest study in this series by Dong et al. established a palladacycle complex directly equipped with the aryl moiety (2-acetanilidyl) and the ligand (P­(Ad)_3_) (Table 4, entry 14).^55^ Both bromide- and iodide-based monomers were tested using various bases (K_2_CO_3_, K_3_PO_4_, Na_2_CO_3_/Cs_2_CO_3_) and monomer-to-catalyst ratios (eight to 50). In summary, the comprehensive work of this series demonstrated the possibility of obtaining molar masses from three to 31 kg/mol with low dispersity values, which indicates the dominating chain-growth pathway, and up to 47 kg/mol with higher dispersity values, which suggests the contribution of step-growth (*vide supra*).

Huber et al. also utilized the Buchwald-type precatalyst with the tri­(*tert*-butyl)­phosphine ligand (Table 4, entry 15).^56^ The authors added 4-phosphonatephenyl bromide and used an octyl-decorated Br/Bpin-based monomer. They compared the catalyst system to the *ex situ* prepared one and tested two bases (K_2_CO_3_ vs CsF) and varying monomer-to-catalyst ratios (five to 20). Despite moderate yields, low dispersity values are reached (assigned to chain-growth mechanism) in a range from two to twelve kg/mol, while higher molar masses (17 kg/mol) featured also higher dispersity values.

Kobayashi et al. performed the polymerizations with a hitherto unusual boronate salt monomer (Br/[B­(OCH_2_)_3_CCH_3_]^−^) in combination with Pd_2_(dba)_3_ precatalyst with P­(^*t*^Bu)_3_ and aryl halide (Table 4, entry 16).^57^ The authors compared the boronate salt to the more usual boronic acid pinacol ester and tested a range of aryl iodides (4-*R*-phenyl iodides), in parts based on polymers (up to four halides). Different equivalents of base (zero to two) and water (one to 600) were evaluated at four temperatures between −30 and 20 °C as well as monomer-to-catalyst ratios (20 to 450). They reached chain-growth assigned molar masses from five to 21 kg/mol with dispersity values below 1.2, but also a remarkably low dispersity for a very high molar mass of 69 kg/mol (1.38).

Delabie et al. 2021 studied two initiating aryl halides at various monomer-to-catalyst ratios from three to 30, utilizing the well-defined “P­(^*t*^Bu)_3_ Pd G2” catalyst system in aqueous THF and K_2_CO_3_ as the base at 20 °C for 16 hours (Table 4, entry 17).^58^ The authors found that the molar mass increased with the monomer-to-catalyst ratio but also led to higher dispersity values as high as 1.60.

The Nesterov group recently published two comprehensive reports on the use of MIDA-protected monomers and their activation. Howell et al. 2023 applied a RuPhos-based Ar–Pd­(L)–I initiating catalyst system and tested the role of Ag_2_SO_4_ to promote SCTP in detail under various conditions.^59^ It was found that longer reactions times up to three days were required. Later, Howell et al. 2024 further explored different aryl halides and catalyst systems for the initiation and polymerization stages.^23^

## 5 Global Analysis of Data Set Refined to Monomer-to-Catalyst Ratio below 25

In this section, a global analysis of the reported polymerization results is presented. The analysis is divided into the main stages of initiation, polymerization, and capping (Scheme 3). The specific parameters can be classified as *monomer* (e.g., type of halide, boron species or side chain), *catalyst* (e.g., precatalyst source, ligand, stoichiometry), and *reaction conditions* (e.g., temperature, time, concentration, solvent, base). Each of these parameters will be evaluated in terms of systematic series from single studies, followed by the analysis and generalization of the findings to the data set. The data set refined by monomer-to-catalyst ratios ≤ 25 will be used (see Section 3.5) to reveal the role of a parameter in terms of SCTP fidelity (see Section 3.4) and to assess whether a limiting effect seems plausible (see Section 3.6). Hence, the representative majority of experiments (403 of 489, 82%) will be covered in this section, while the remaining experiments (84 of 489, 18%) with monomer-to-catalyst ratios > 25 will be analyzed in Section 6.

**Scheme 3:**
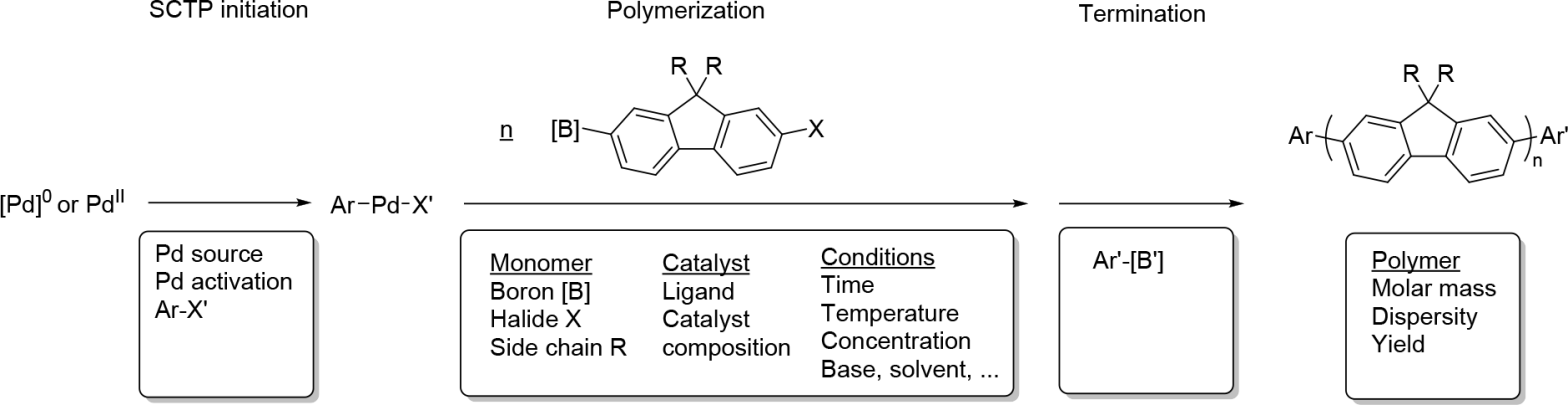
Schematic Representation of the Performed AB-Type Polymerizations toward Poly­(9,9-bis­(side chain)­fluorene-2,7-diyl)­s Following the Main Steps of SCTP Initiation, Polymerization, and Capping with Key Parameters; Parameters are Classified as Monomer, Catalyst, and Reaction Conditions for the Analysis of the Resulting Polymer Properties

### 5.1 Initiation Stage

In the initiation stage (see Section 2.1), a Pd source is converted to the active species for the subsequent Suzuki-Miyaura polycondensation. Frequently, a monofunctional Ar–X’ substrate is used prior to the addition of monomer ensuring the *in situ* formation of a precise α-terminus (Ar) through the corresponding intermediate Ar–Pd­(L)–X’. Alternatively, Ar–Pd­(L)–X’ can be prepared *ex situ* and added as a catalyst. In the absence of such defined initiation, the *in situ* catalyst activation proceeds *via* the monomer (M) and inevitably leads to difunctional Pd species, i.e., X’–M_2_–Pd–X’ (from Pd^II^ salts) or (RO)_2_B–M–Pd–X’ (from Pd^0^ sources). Both species may undergo additional coupling steps at the functionalized α-terminus (*vide supra*), in particular, at high monomer conversions or upon catalyst dissociation. Hence, a broadened polymer distribution and/or deviations from the theoretical molar masses may occur. In the following, the main parameters that affect the (precise) initiation toward Ar–Pd­(L)–X’ will be analyzed, i.e., the precatalyst metal source, the type of Ar–X’, and the corresponding stoichiometric ratios. In the following, the discussion of the catalytic system’s additional parameters on the outcome of the polymerization stage assumes a successful initiation stage (*vide infra*).

#### 5.1.1 Precatalyst Source

Figure 7a displays the systematic comparison in terms of the precatalyst metal source. Only two data series report on different precatalysts: Series A employed Na_2_[PdCl_4_] (#1), [Pd­(MeCN)_2_Cl_2_] (#3), and [Pd­(PhCN)_2_Cl_2_] (#4) as Pd^II^ salts, which lead in all cases to comparably high yields and molar masses but rather high dispersity values (>1.5). Series B employed the *ex situ* prepared dimeric form of Ar–Pd­(P­(*o*Tol)_3_)–X’. The two used complexes differ only in the *para*-substituent of the Ar group, i.e., methoxy (#6) vs tolyl (#7). In both cases, comparable molar masses and rather high dispersity values (>1.5) were found, albeit no isolated yield data is reported. The majority of the data (series C to G) reveal excellent high reproducibility employing the same type of precatalyst metal source (represented by the same abscissa label), as indicated by the comparable obtained molar masses (top), dispersity values (middle), and yields (bottom). Importantly, low dispersity values can be reached for the precatalysts P­(^*t*^Bu)_3_PdG2 (#2), Pd_2_(dba)_3_ (#5), and *o*Tol-Pd­(RuPhos)-I (#8).

**Figure 7:**
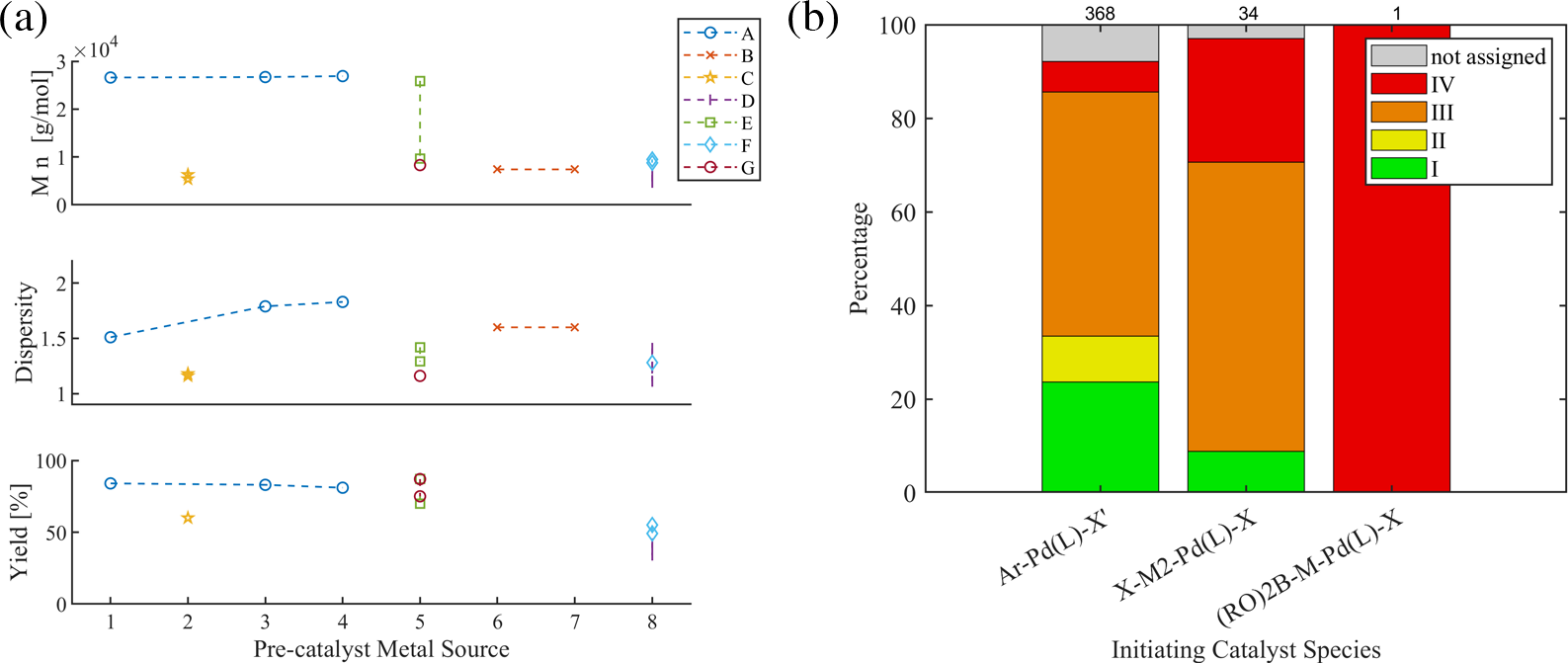
(a) Overview of systematic studies (≥2 datapoints/series, dashed lines applied to connect datapoints) on the effect of the precatalyst source. (b) Categorization of the refined data set by the formed initiating catalyst species.

Figure 7b summarizes the corresponding categorization of the refined data set. The majority of datapoints is based on the well-defined active Ar–Pd­(L)–X’ species (368 entries), while the less-defined initiation conditions seem to lead to less-defined SCTP fidelity according to the dominant categorization of “III” and “IV”. The role of Ar–X’ is studied in depth, because any functional groups at the Ar moiety enable postpolymerization coupling steps at the α-terminus, as well as diagnostic groups can be introduced, e.g., for UV–vis detection or mass spectrometry analysis. In the following, the SCTP outcome will be evaluated in terms of the Ar groups and type of X’.

#### 5.1.2 Ar–X’ Aryl Group

The effect of the Ar group is displayed in Figure 8a, for which series with ≥5 datapoints were selected to assist clarity. The selected six series (A-F) contain in total 74 entries with 36 different Ar groups. The detailed interpretation of the chemical structures of the Ar group is beyond the scope of this study, and thus, the groups are abbreviated by numerals at the abscissa. First, the series A-D reveal comparable molar masses, dispersity values, and yields, illustrating an inferior role of the Ar group in the respective applied reaction conditions. For example, series B revealed the lowest dispersity values (*Đ* ≤ 1.19). The series E and F show significantly more scattered values for the obtained molar masses, reported yields, and higher dispersity values (up to 1.67). Next, the potential limitations can be estimated upon comparison of the entries that belong to multiple series. Two representative selections are marked (Figure 8a, blue boxes). In the case of entries #18 to #20 (left box), a consistently more defined SCTP character can be seen for the same Ar groups in series B (red) vs series E (green), which must originate from other parameters. On the other hand, following series B also reveals entries with comparably low dispersity values (entries #26 to #28) suggesting the other parameters are not inherently limiting the SCTP, but the Ar group does have an impact. A similar scattered trend is observed for entries #24 to #34 (right box). At this stage, a full analysis is beyond the scope due to the limited data available.

**Figure 8:**
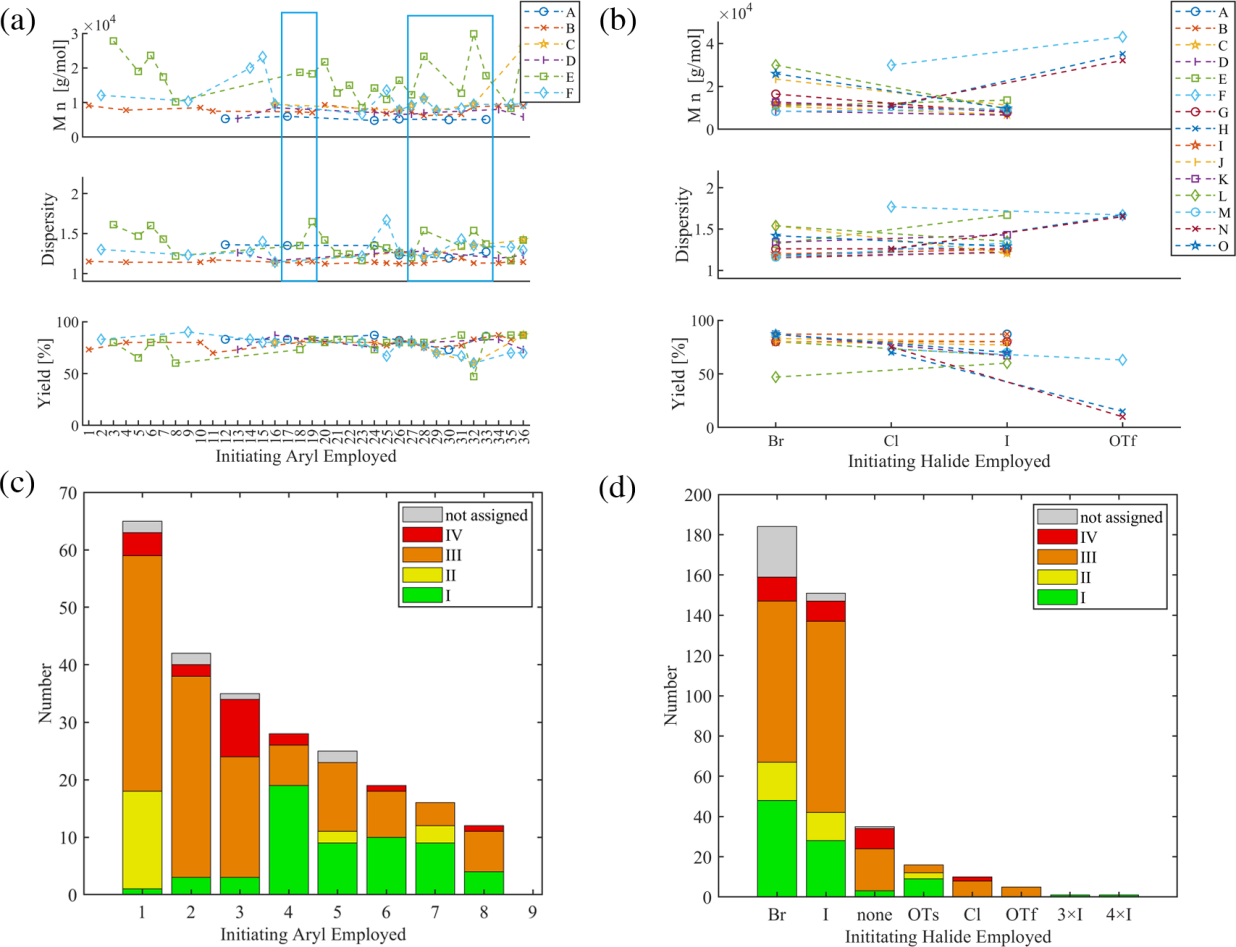
Overview of systematic studies on Ar–X’: (a) Effect of the Ar group (≥5 datapoints/series, dashed lines applied to connect datapoints). Blue boxes illustrate selections discussed in the text. (b) Effect of the X’ group (dashed lines applied to connect datapoints). (c) Metrics of the refined data set for the Ar group for the most often used Ar groups. (d) Metrics of the refined data set for the X’ group. See Supporting Information for the complete data.

#### 5.1.3 Ar–X’ Halide

The effect of the halide X’ is depicted in Figure 8b. Most series compared Br vs I pairwise, while only three (series F, H, N) contain data for Cl vs OTf. In the case of Br vs I, the obtained yield is comparable, but the molar masses and dispersity values were typically higher for Br than for I. Assuming identical polymerization behavior, the usage of Ar–I seems to be beneficial in terms of SCTP performance. In the case of Cl vs OTf, the situation is less clear. Lower yields and higher molar masses were obtained for OTf, and the dispersity values appear to be higher or at least comparable. Such behavior is expected if coupling steps contribute to the chain-growth polymerization and might also be induced by an incomplete initiation stage (*vide supra*).

#### 5.1.4 Stoichiometry of Ar–X’

The effect of stoichiometry of Ar–X’ vs catalyst was examined in one systematic report (Figure 9).^53^ The authors applied the catalyst system Pd_2_(dba)_3_/P­(^*t*^Bu)_3_ and varied the ratio for selected Ar–X’ substrates pairwise, with other identical conditions. In series A (Ar = 4-Br-Ph; X’ = I), the small ratio change from 0.83 to 1.67 led to comparable molar masses close to the expected value, low dispersity values, and high yields. Next, the less reactive halide X’ = Cl was studied for Ar = 4-CH_3_-Ph (series B) and electron-deficient Ar = 4-COOEt-Ph (series C). Applying the same ratio of 1.67 as that for series A revealed substantially higher molar masses and higher dispersity values, but the yields for series B were also unexpectedly low. These results illustrate the impact of an insufficiently effective intiation. At a higher ratio of 25, series B revealed lowered molar masses closer to the expected values and lower dispersity values comparable to series A, while series B remained at high molar mass and high dispersity but low yield (30%). To check if the Ar group is limiting, X’ = OTf was examined (series D), which reproduced a very similar behavior despite the higher yield at a ratio of 1.67. It thus appears that the electron-deficient effect of the ester substituent overlaps the effect of X’ or the stoichiometric ratio. Hence, the final series employed X’ = Cl for five stoichiometric ratios (series E). It is demonstrated that an increasing amount of Ar–X’ leads to a more defined SCTP behavior, i.e., molar masses close to the expected values, higher yields, and low dispersity values. The authors assign this finding to the enhanced initiation, and a separate investigation could confirm this plausible explanation. In addition, a higher stoichiometry of Ar–X’ would also be beneficial in the case of an incomplete initiation stage or catalyst dissociation, because the released Pd fragment can “re-initiate” also during the polymerization stage, leading to a higher SCTP fidelity. Hence, the initiation of new chains due to the excess of Ar–X’ would narrow the polymer ditribution as shorter chains are also expected to grow faster (*vide supra*).

**Figure 9:**
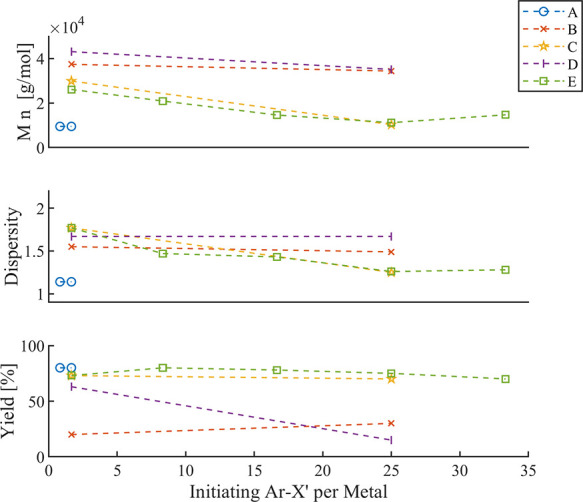
Overview of systematic studies on stoichiometric ratios of various Ar–X’ vs catalyst.

### 5.2 Polymerization Stage

The previous section revealed the sufficient initiation, if a Pd^0^ precatalyst is utilized in combination with Ar–X’ with X’ = Br or I, and Ar groups with inert substituents. In addition, an excess of Ar–X’ seems beneficial to ensure complete initiation. In this main chapter, the parameters that are associated with the monomer, the catalyst system, and the reaction conditions including stoichiometry will be analyzed for both systematic series and the complete refined data set (monomer-to-catalyst ratio ≤ 25).

#### 5.2.1 Monomer-Related Parameters

The monomer-related parameters were classified as the type of halide X, the boronic acid species [B], and the type of side chain R. Since the overall reaction proceeds and consumes many equivalents of the monomer, the type of released halide anion accumulates, the boronic acid species [B] is to be hydrolyzed but not deborated, and the side chain R shall not impede the ring-walking step. We hypothesize that a minor effect in each step will progressively contribute sizably toward the discontinuation of the idealized SCTP behavior.

##### 5.2.1.1 Monomer Halide X

The role of the halide X is typically assigned to the rate of oxidative addition and thus affects the ring-walking step in terms of the driving force. In addition, the released X^–^ might act as a competing ligand to form dormant Pd species, which is expected to slow down the conversion in particular at the late stage of the polymerization.

Four series by the groups of Hu and Hong were found that systematically examined the effect of bromide vs iodide (Figure 10).^53–55^ The eight reactions were performed under comparable conditions, i.e., at 0 °C for 0.5 to 1 hours in THF/water mixtures using aqueous K_2_CO_3_ or K_3_PO_4_ as the base. The well-performing Pd_2_(dba)_3_/P­(^*t*^Bu)_3_ catalyst system was used, differing only in the aryl halide for initiation (see Section 5.1).^53^ Series A (4-Br-Ph–I as Ar–X’) revealed identical yields (80%) but lower molar masses and a higher dispersity for X = I. Series C (4-Ph­(CO)-Ph–Br as Ar–X’) reports a lower yield, lower molar masses, but a comparable dispersity. In the case of series D, using a similar catalyst system P­(^*t*^Bu)_3_ Pd G2 and 4-COOEt-Ph–Br as Ar–X’, the polymerizations led to a lower yield, a comparably low dispersity, but slightly higher molar masses for X = I.^54^ Finally, series B explored a well-performing catalyst system based on the P­(Ad)_3_ ligand (see Section 5.2.2.1).^55^ The obtained dispersity values were comparably low, but the significantly lower molar masses and the low yield suggest an inhibited polymerization rate in the case of X = I. The limited data available for systematic monomer halide studies prevent a clear assignment of beneficial effects by using either bromide or iodide, despite the fact that iodide leads to (somewhat) broader distributions of lower yields. The lack of representative data is also found in the refined data set, which contains only five experiments using iodide. Hence, the categorization according to X is deliberately omitted because the systematic series were not fully consistent, and other parameters can have a higher contributing effect.

**Figure 10:**
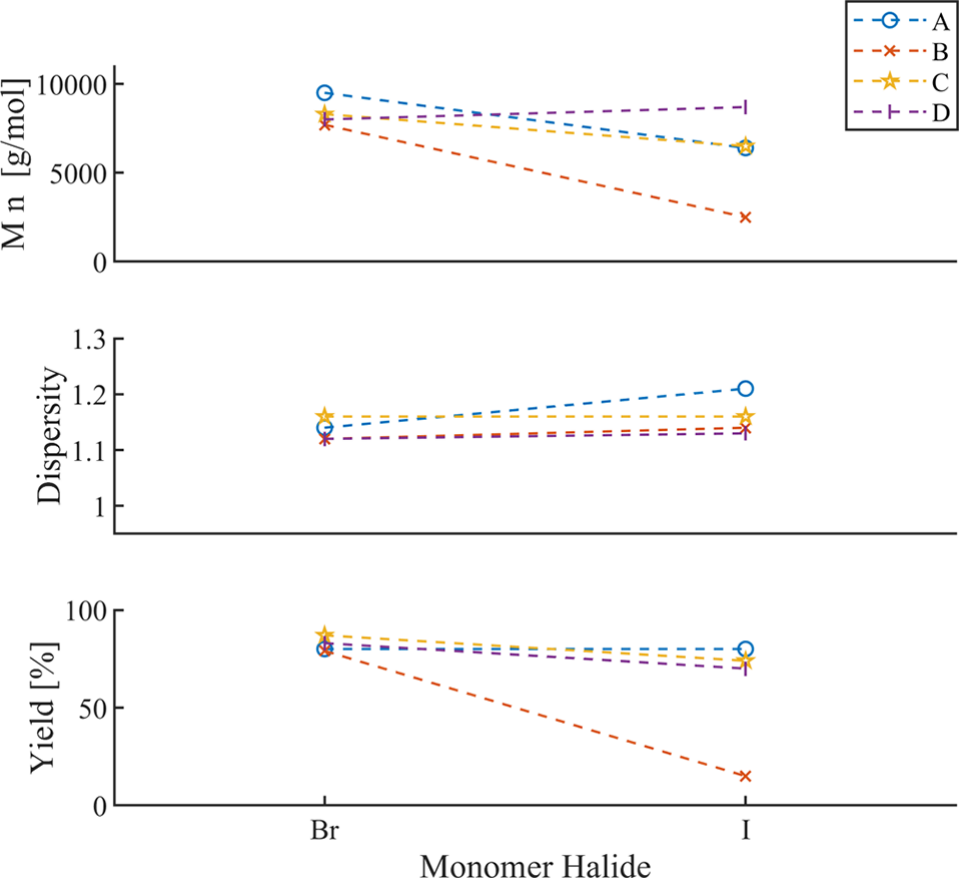
Overview of systematic studies on the effect of the monomer halide.^53–55^

##### 5.2.1.2 Monomer Boronic Acid Species [B]

The boronic acid derivative [B] participates in the transmetalation step. The monomer synthesis typically yields the often-commercialized pinacol boronic acid ester (Bpin) that undergoes hydrolysis under aqueous conditions, yet the free boronic acid may be used accordingly. Other [B] groups (Figure 11a) were also investigated, often applying specialized conditions aiming to tune the hydrolysis of the [B] group. Hence, the precise role of [B] is not compared in great depth in the given data set. Recently, one systematic study investigated the effect of the free boronic acid vs its MIDA-protected derivative (MIDA is *N*-methyliminodiacetic acid).^23^ Within the three series (Figure 11b), the same catalytic system and Ar–X’ were used, but the base and Ag_2_SO_4_ as an additive were varied. In the presence of Ag_2_SO_4_ and Cs_2_CO_3_ (series A), free boronic acid B­(OH)_2_ gave a low yield and a high dispersity value. Although this poor SCTP fidelity was improved using the MIDA derivative instead, the yield (39%) and dispersity value (1.41) are still not satisfying. Applying K_3_PO_4_ as the base (series B) improved the yields for B­(OH)_2_ significantly, but the dispersity remained high. In contrast, the MIDA derivative resulted in a remarkably improved low dispersity value (1.16), despite the low yield which might be enhanced in the future in other ways. The stability of the MIDA group under conventional reaction conditions using aqueous K_3_PO_4_ is evident from the negligible yields when compared to those using the free boronic acid (series C).

**Figure 11:**
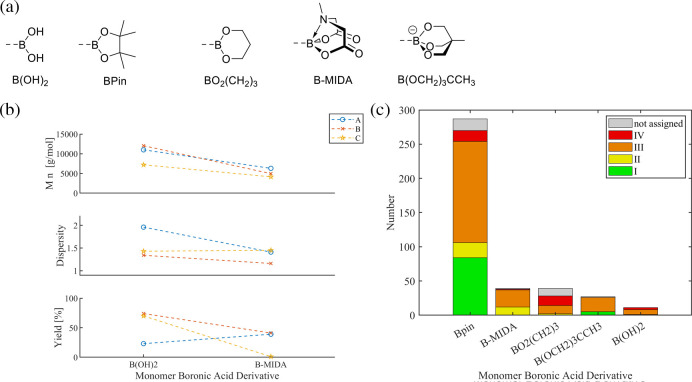
(a) Schematic representation of the chemical structures of typical boronic acid derivatives [B]. (b) Overview of systematic studies on the effect of the monomer boronic acid derivative. (c) Metrics of the refined data set for the [B] group.

The refined data set (Figure 11c) confirmed the dominant role of Bpin in literature (287 entries), but also a sizable number of experiments are reported that utilize B-MIDA (39 entries), propanediol ester BO_2_(CH_2_)_3_ (39 entries), the boronic acid trimethylol ethane ester salt [B­(OCH_2_)_3_CCH_3_]^−^ (27 entries), and the free boronic acid B­(OH)_2_ (11 entries). Generally, good SCTP fidelity was reported for all but the propanediol ester (categories I and II), albeit this apparent exception should be taken with care considering the different employed reaction conditions. The categorization of the data reveals that the best results (category I) were achieved using Bpin, B­(OH)_2_, and [B­(OCH_2_)_3_CCH_3_]^−^. Notably, the boronic acid ester salt (trimethylol ethane) displays an excellent SCTP behavior even at significantly higher monomer-to-catalyst ratios, up to 450; i.e., a linear increase of the molar mass was found, and the dispersity values did not exceed 1.38. Comparing the available data for Bpin and [B­(OCH_2_)_3_CCH_3_]^−^, the boronic acid ester salt outperforms the benchmark boronic acid pinacol ester, which was not tested for high monomer-to-catalyst ratios when employing an aryl halide. Note that polymerizations using the boronic acid ester salt were performed at −10 °C in contrast to boronic acid pinacol ester, for which 0 °C was revealed to be superior to −10 °C (see also Chapter 5.3.2).

##### 5.2.1.3 Monomer Side Chain Group R

The side chain group of the monomer affects the steric demand at the repeating unit. In contrast to the halide and boronic acid groups, the side chain group remains in the latter polymer to ensure solubility or processability and, thus, is typically selected to ensure the desired properties. As a consequence, systematic variation of the side chain in terms of polymerization behavior is rare. Nonetheless, the steric bulk may affect or even block the ring-walking step. Figure 12a displays the systematic studies. All studies (A to G) compared pairwise *n*-hexyl (2) vs *n*-octyl (3) under various conditions, whereas series C also included methyldiethyleneglycol (1). In terms of the reported high yields, no effect of the side chain could be deduced. The studies indicate slightly higher dispersity values and higher molar masses for the larger *n*-octyl group (3). The analysis of the refined data set (Figure 12b) parallels the trend of a a higher SCTP fidelity for the *n*-hexyl group (1) vs *n*-octyl (2), although the effect is likely dominated by other parameters.

**Figure 12:**
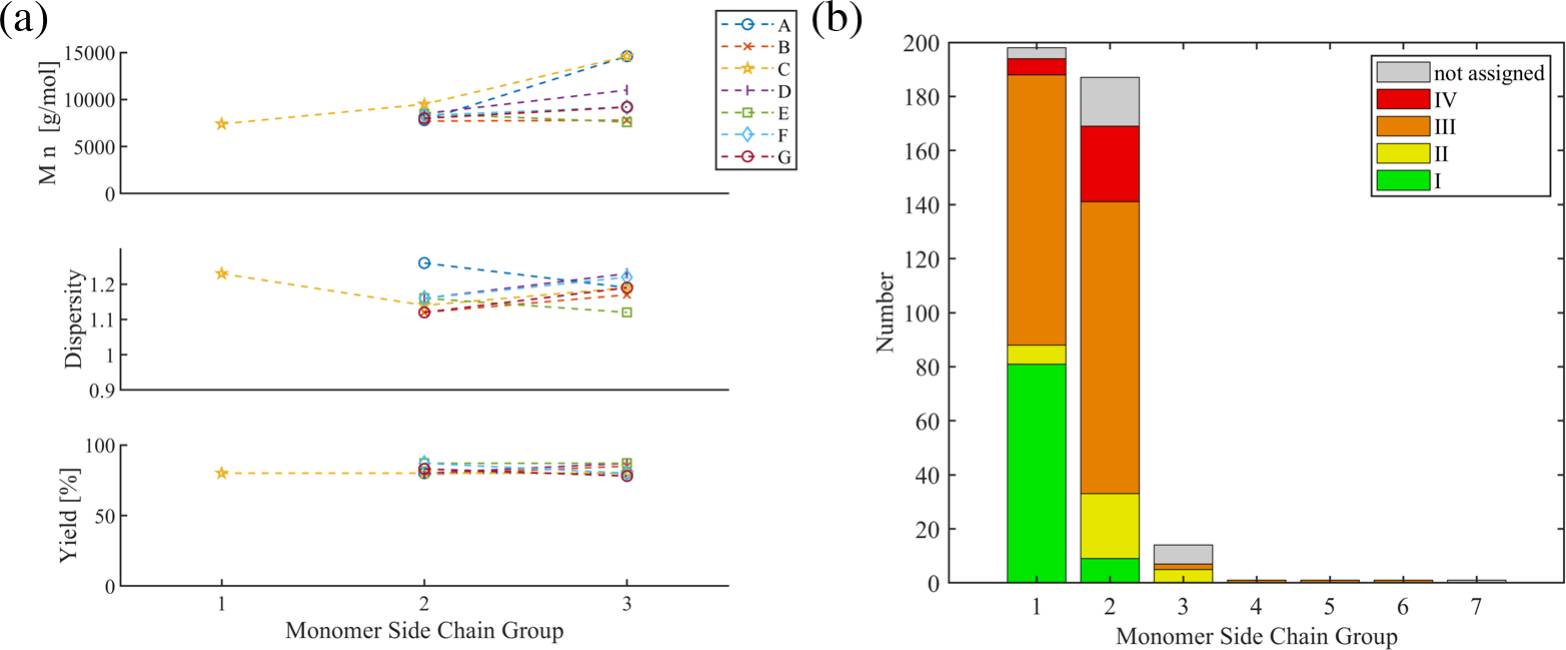
(a) Overview of systematic studies on the effect of monomer side chain R. (b) Metrics of the refined data set for the side chain R.

#### 5.2.2 Catalytic System

The importance of the initiation stage is described in Section 5.1, i.e., the source of the precatalyst as well as the nature and stoichiometry of activation by monoaryl halides. Although additional parameters will contribute in that early stage, any effect will accumulate during the (multiple) SCTP cycles in the polymerization stage. Hence, this section analyzes the impact of the nature and stoichiometry of the Pd ligands, the employed bases, and further additives. The interplay of the catalyst and monomer as well as general reaction conditions will be discussed afterward.

##### 5.2.2.1 Type of Ligand

The ligand of the Pd center is one major parameter to tune the reactivity and stability of the catalytic system via electronic and steric contributions. Figure 13 displays the chemical formulas of reported ligands. Surprisingly, no systematic study was found that compares different ligands.

**Figure 13:**
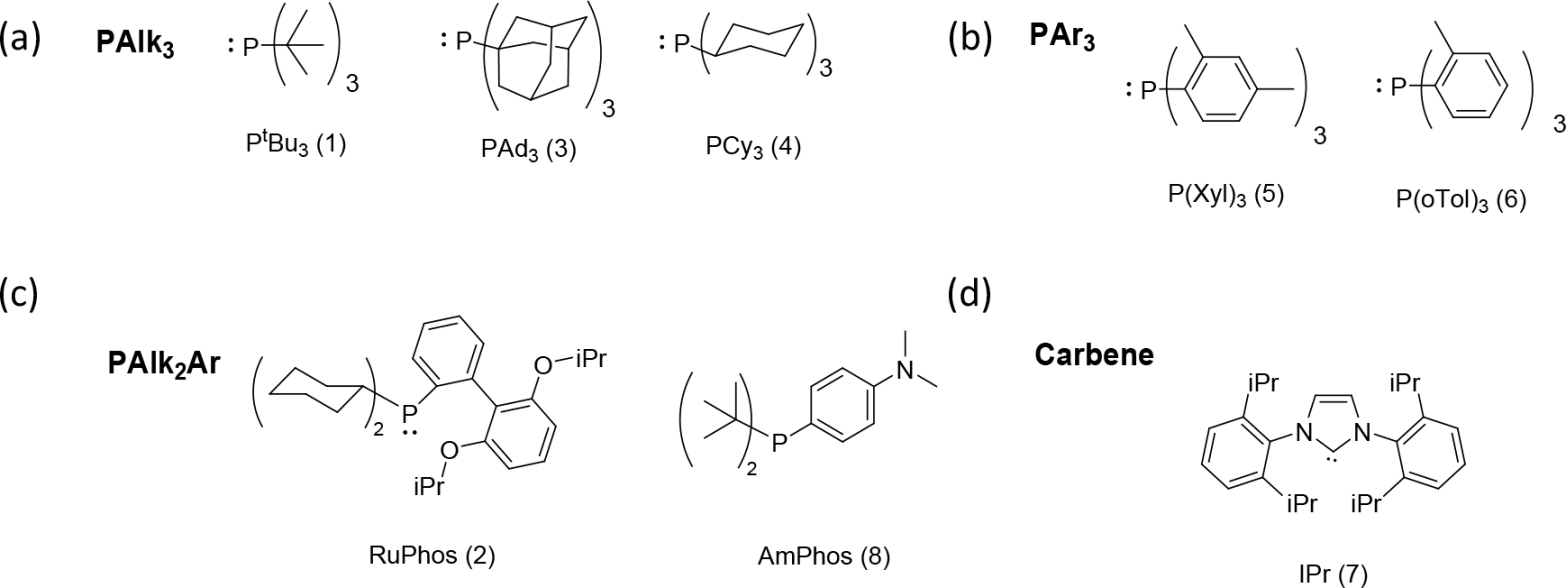
Schematic representation of the chemical structures of the Pd ligands classified by types: (a) tris-alkyl phosphines, (b) tris-aryl phosphines, (c) mixed bis-alkyl-aryl phosphines, and (d) carbenes.

The categorization of the employed ligands depicted in Figure 14 revealed first that P­(^*t*^Bu)_3_ (1) is the most frequently utilized ligand, followed by RuPhos (2), whereas the remaining *tris*-alkyl and *tris*-aryl phosphines as well as carbenes play an inferior role. The highest SCTP fidelity was reported for *tris*-alkyl phosphines bearing bulky *tert*-butyl or adamantyl groups. Low dispersity values (*Đ* ≤ 1.20) together with high yields (≥70%) are found only for *tris*-*tert*-butylphosphine and *tris*(1-adamantyl)­phosphine. Further low dispersity values were reached for tricyclohexylphosphine, but yields are low (<30%). Both triarylphosphines (P­(Xyl)_3_ and P­(*o*Tol)_3_) yield broad to moderately broad distributions in reasonable molar mass ranges. The polymerizations using AmPhos resulted in moderate dispersity values within a reasonable molar mass range.

**Figure 14:**
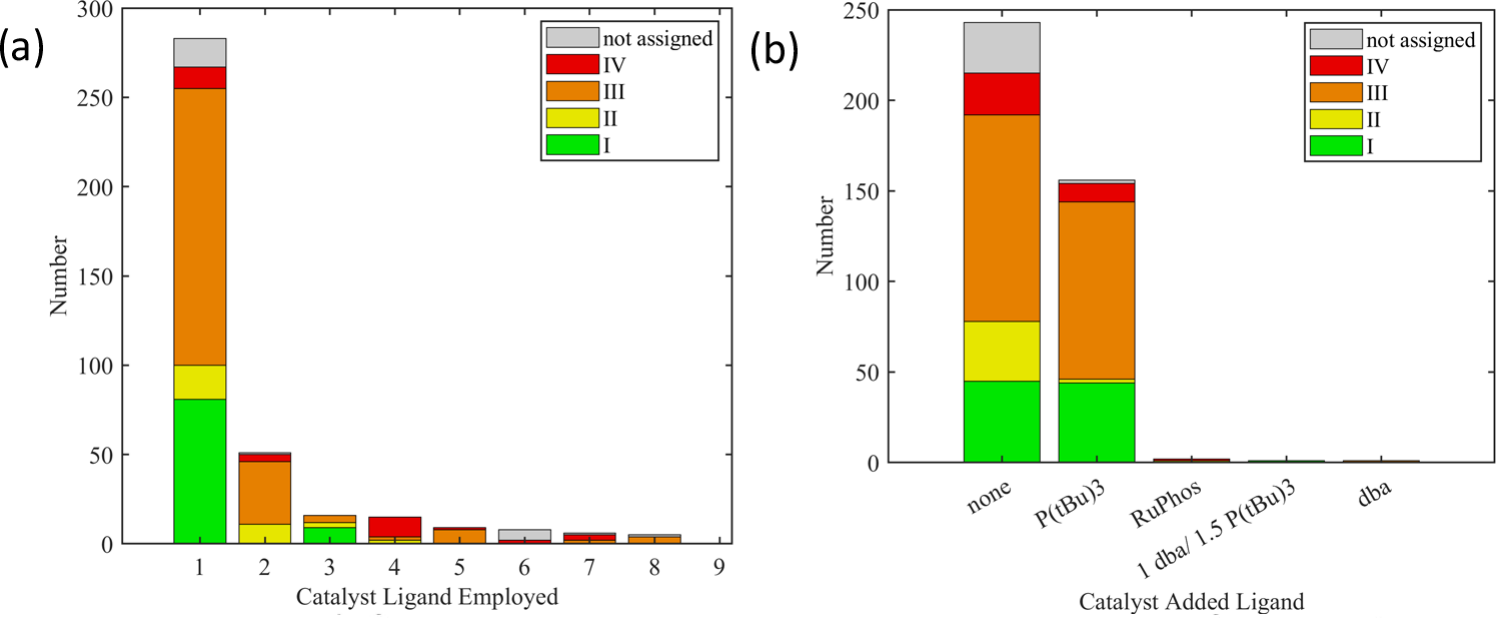
Categorization of the refined data set sorted by the most-employed ligands: (1) P­(^t^Bu)_3_, (2) RuPhos, (3) P­(Ad)_3_, (4) P­(Cy)_3_, (5) P­(Xyl)_3_, (6) P­(oTol)_3_, (7) IPr, and (8) AmPhos.

This behavior is in line with the high Tolman angle or likewise the steric demand at the alpha-carbon.^50^ It can be concluded that tri-*tert*-butylphosphine and P­(Ad)_3_ support the SCTP mechanism, while P­(Cy)_3_ does not. However, P­(Cy)_3_ was utilized using a different boronic acid ester moiety, and thus, the steric impact may be hidden by the type of boronic acid ester. Likewise, the only few aromatic phosphines (tri-*ortho-*tolylphosphine P­(*o*Tol)_3_, tri-(2,4-dimethylphenyl)­phosphine P­(Xyl)_3_) also utilize different boronic acid moieties, and the same argumentation may hold for P­(Cy)_3_ (see Section 5.2.1.2). In the case of AmPhos, the limited data prevent a definite assignment.

##### 5.2.2.2 Stoichiometry of Ligand per Pd

In addition to the type of employed ligand, the number of equivalents per Pd center (expressed as the ligand-to-metal ratio) may also influence the overall polymerization results. Although additional ligands may stabilize the active catalyst by formation of coordination-saturated dormant species, this can also lead to the formation of dormant species in analogy to the buildup of halide X at high conversions (see Section 5.2.1.1). If so, the blocking of vacant coordination sites at Pd needed during the crucial ring-walking step via weak π-interactions is expected to have a profound effect.

Grisorio et al. 2014 reported three series (Figure 15a, series A to C) which utilized a Pd^2+^-based precatalyst and P­(^*t*^Bu)_3_ as ligand without a monoaryl halide for initiation. The difference arises from the order of mixing of Pd, ligand, and K_3_PO_4_ as a base in aqueous THF at 20 °C for 5 to 10 minutes. The obtained molar masses were reproducible, and the yields were comparably high (±5%), but the varying dispersity values of 1.28 to 2.53 revealed little control in terms of SCTP fidelity. The best result (*Đ* = 1.28) was found for two equiv belonging to category III. It seems that the formation of the catalyst system during initiation governs the outcome of the reaction (see section 5.1). The results reported by Zhang et al. in 2012 (series D) and 2015 (series E) are based on the more defined initiating catalyst species Ar–Pd­(L)–X’ as well as milder reaction condition (*vide supra*) and low monomer-to-catalyst ratios. We interpret the identical values for molar masses, dispersity values, and yields reported in both series to originate from the same experiments. Nonetheless, the SCTP performance was best for a ligand-to-metal ratio of 2:1 which parallels the observation by Grisorio.

**Figure 15:**
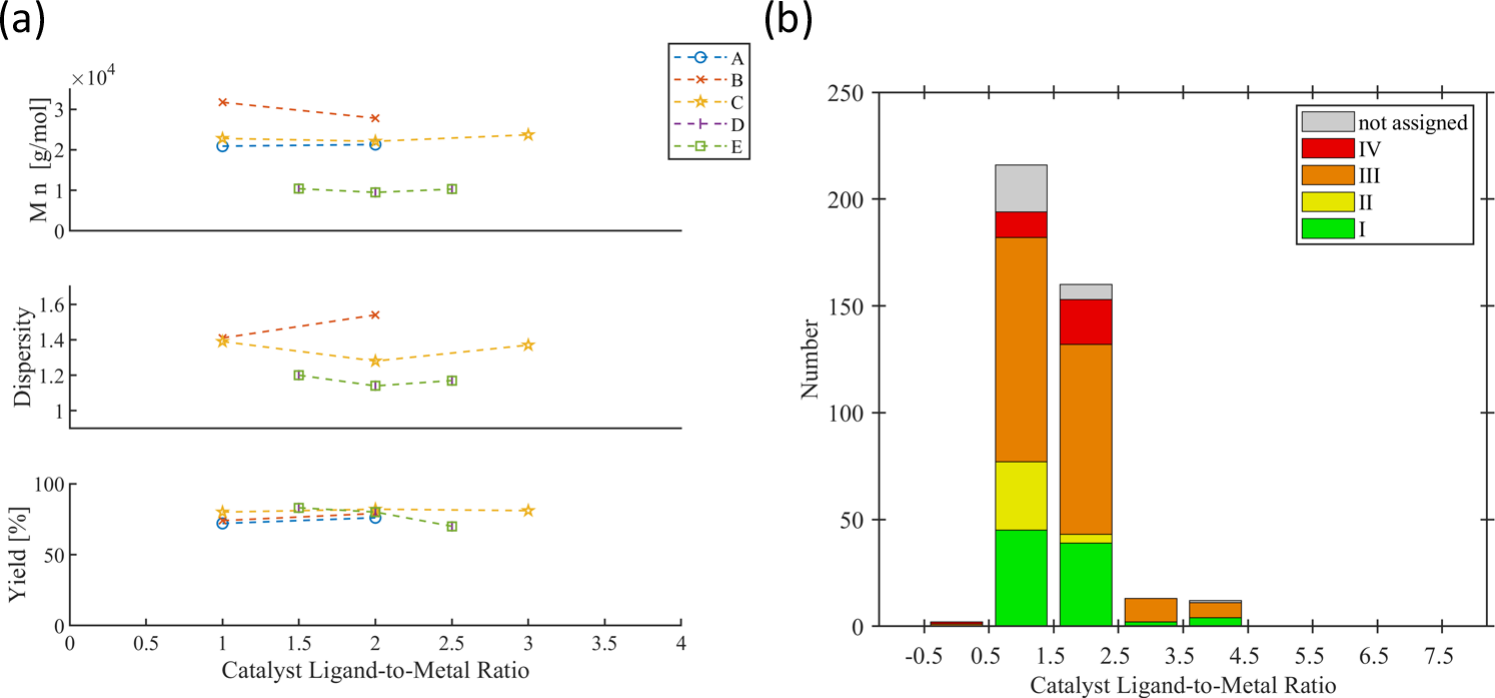
(a) Overview of systematic studies on the effect of the ligand-to-metal ratio reported by Grisorio et al. 2014 (A, B, and C), Zhang et al. 2012 (D), and Zhang et al. 2015. (b) Categorization of the refined data set binned by the ligand-to-metal ratio.

The binned categorization data displayed in Figure 15b revealed that most experiments used ligand-to-metal ratios between 0.5 and 2.5, but the relative contributions of the categories indicate no major impact. Hence, the large impact of the ligand-to-metal ratio on the dispersity values found by Grisorio et al. (series A-C) is likely to originate from the variation in the formation of the initiating catalyst species. This is plausible in view of the typical conditions that already embark from such defined Pd species and, thus, are not heavily affected by the ligand-to-metal ratio.

### 5.3 Reaction Conditions

In the preceding sections, the monomer- and catalyst-related parameters were discussed and classified in terms of their chemical nature and stoichiometry. Further influence is expected from the reaction conditions, e.g., reaction time, temperature, solvents, bases, or concentration.

#### 5.3.1 Polymerization Time

The polymerization time is one parameter that controls the monomer conversion of the reaction. A high initial monomer availability favors the SCTP mechanism at early times. Once high conversion is reached, the chain propagation slows down, and potential unimolecular side reactions may start to play a major role, e.g., *retro*-oxidative addition to release catalytically active species similar to inefficient ring-walking (see Section 2.1).

For poly­(fluorene)­s, many studies compare the effects of reaction times. Because the rate of reaction also depends on other reaction conditions, the comparison of values between series should be taken with care and varies considerably from minutes (Figure 16a) to days (Figure 16b). Two series detail the course of the reaction within one hour. Ying et al. (series A) applied an *ex situ* prepared Ph–Pd­(P­(^*t*^Bu)_3_)–Br initiating catalyst species in aqueous THF with Na_2_CO_3_ as the base at 25 °C, using a monomer concentration of 17 mmol/L and a monomer-to-catalyst ratio of 20 (series A).^46^ After only two minutes, 86% yield was already obtained. For the five minute experiment, a small increase to 92% was observed. The corresponding molar masses increased monotonously, and the dispersity values were low throughout the series, around 1.3. The obtained molar masses around 15 kg/mol agree reasonably well with the expected value of 1 kg/mol per incorporated repeating unit given by the initial monomer-to-catalyst ratio (20) and the conversion (92%). Zhang et al. (series E) studied an *in situ* (P­(^*t*^Bu)_3_) Pd G2 with a 4-Tol-Br initiating catalyst system in aqueous THF with K_3_PO_4_ as the base at 0 °C, also using a monomer concentration of 17 mmol/L but with a lower monomer-to-catalyst ratio of 8.^54^ Within the studied time ranging from 20 to 60 minutes, the reaction was already complete as indicated by the comparably high yields (>80%) and low dispersity values (<1.3). Surprisingly, the consistent molar masses (around 12 kg/mol) were somewhat higher than expected, as the monomer-to-catalyst ratio was only eight and conversion reached 83%. Two more series exemplarily compared short reaction times. In the case of series B, a significant improvement from 0.5 to one hour was observed in terms of yield, while the dispersity value remained as low as 1.12. In the case of series I, no major difference was observed in the very narrow time range. The remaining series generally studied longer reaction times. The Nesterov group studied the MIDA-derivative under various conditions (series C, F, G, and H), which is prone to the slow release of the boronic acid required for the transmetalation step. Higher yields and higher molar masses were indeed obtained at long reaction times, but the dispersity value increased to approximately 1.5.

**Figure 16:**
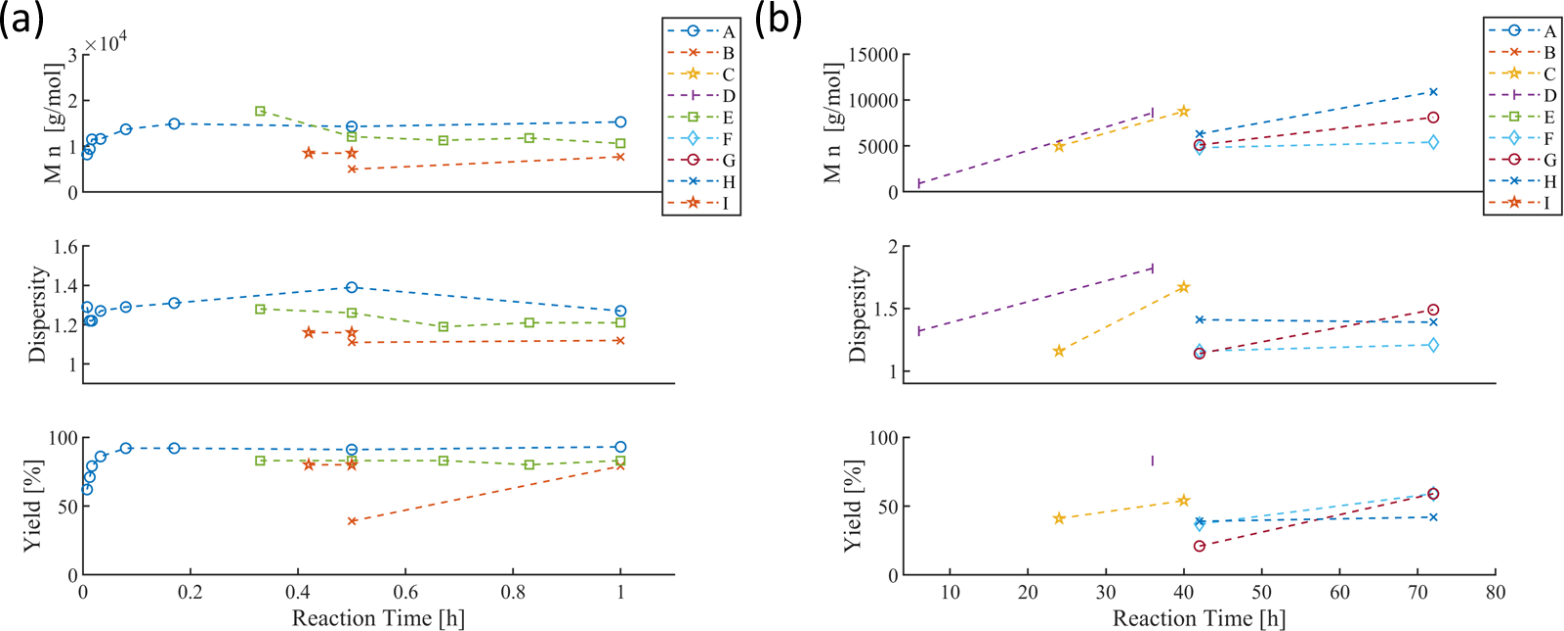
Overview of systematic studies on the effect of the reaction time: (a) Fast reactions within one hour and (b) long reactions up to three days.

The categorization of the reaction times (Figure 17) shows that most studies were performed below two hours leading to a high SCTP fidelity as expressed by category I (green). At longer reaction times, fewer experiments were conducted, and lower SCTP fidelity was obtained, which is assigned to concomitant side reactions at longer time scales.

**Figure 17:**
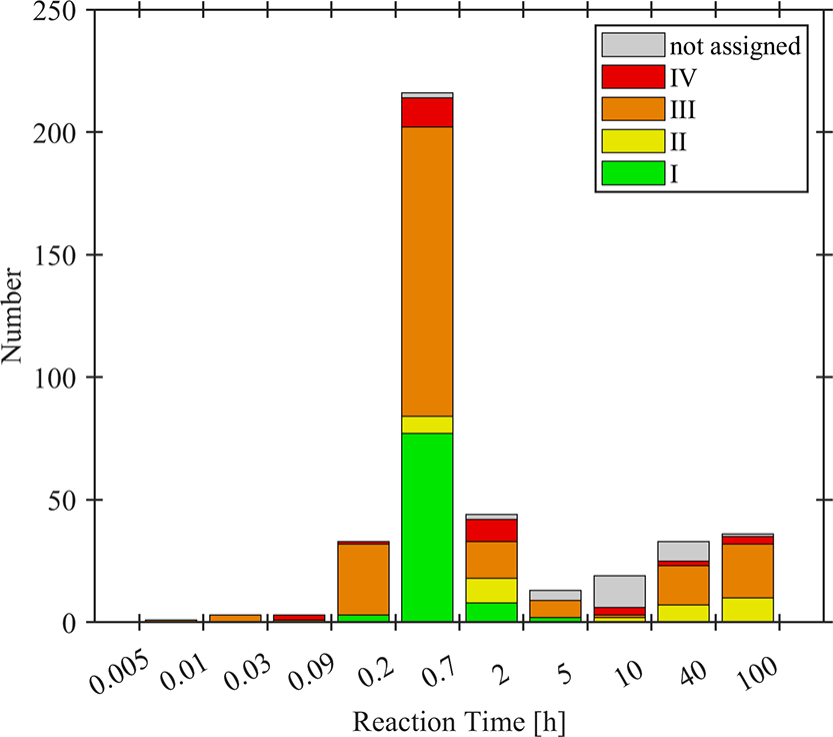
Categorization of the refined data set binned by the reaction time.

#### 5.3.2 Temperature

The reaction temperature also influences the steps during the polymerization, e.g., setting the subtle balances between the temperature-dependent diffusion coefficients of bimolecular reactions vs unimolecular (side) reactions and providing the thermal energy to pass activation barriers—in particular the weak association during the ring-walking steps. Within the refined data set, five series were found that reported on the polymerization temperature between −30 and 20 °C (Figure 18a). Two studies reported identical values (series D^51^ and E^53^) and will be treated as the same result. For some datapoints, data on molar mass, dispersity, or yield are missing, and two more series also report on elevated temperatures but applied a higher monomer-to-catalyst ratio (not shown).^45,59^ Hence, the following analysis is based on the three series A, C, and D.

**Figure 18:**
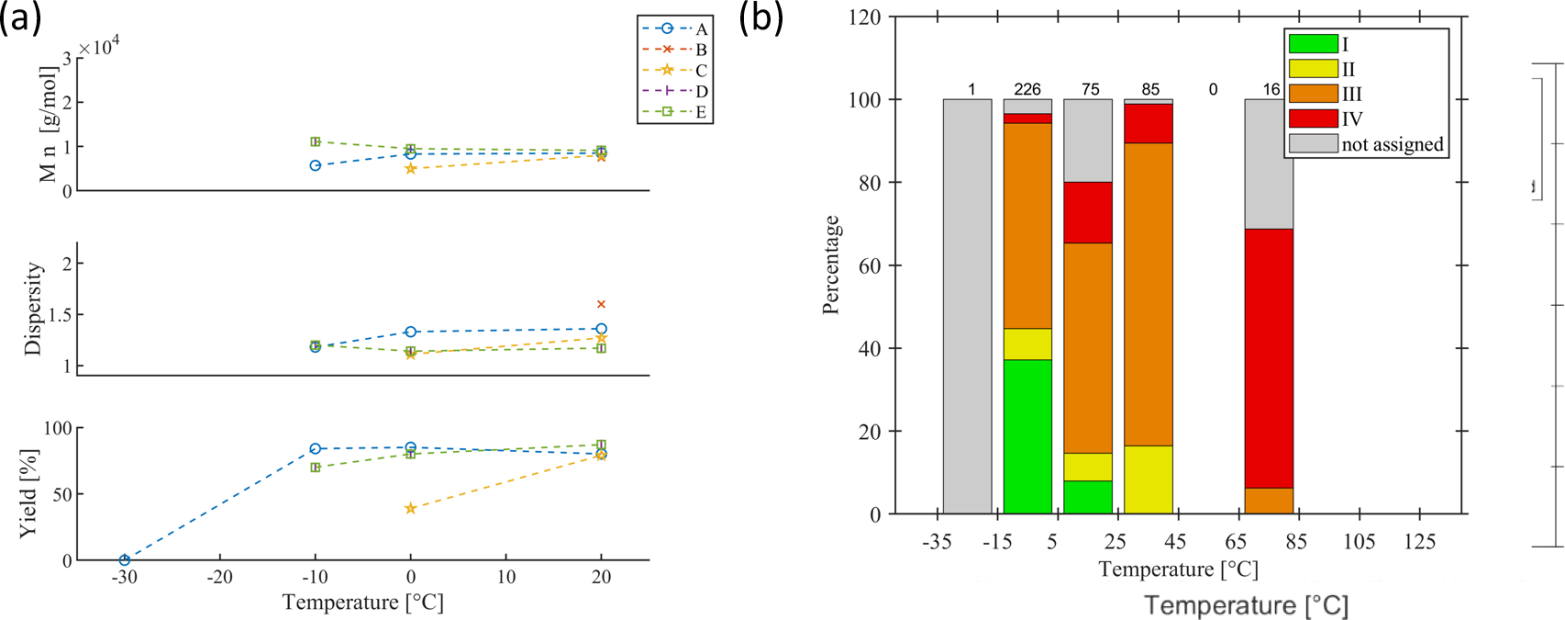
(a) Overview of systematic studies on the effect of the reaction temperature. (b) Categorization of the refined data set binned by the reaction temperature.

Kobayashi et al. explored the polymerization of the exceptional boronate-based monomer under well-defined conditions, namely starting from a P­(^*t*^Bu)_3_-based Ar–Pd­(L)–X’ initiating catalyst system (series A).^57^ Within the short polymerization time of 15 minutes, no polymer could be isolated at the lowest temperature (−30 °C), but good yields (80 to 85%) were obtained above −10 °C. The corresponding dispersity values increased at higher temperatures; i.e., the lowest *Đ* was found at −10 °C (1.18), whereas the increased values at 0 and 20 °C did not differ significantly. In line with this observation, the obtained molar masses were lower at −10 °C (5.7 kg/mol) and comparable at 0 °C and 20 °C (8.3 kg/mol and 8.5 kg/mol, respectively). Dong et al. (series C) applied the P­(Ad)_3_-based reported Ar–Pd­(L)–X’ initiating catalyst system for the polymerization of the standard Bpin-based monomer within 30 minutes. It was found that lower temperatures (0 °C vs 20 °C) led to lower dispersity values (1.11 vs 1.27), lower molar masses (5.0 kg/mol vs 8.0 kg/mol), and lower yield (39% vs 79%). Zhang et al. compared the P­(^*t*^Bu)_3_-based Ar–Pd­(L)–X’ initiating catalyst system (series C) for the polymerization of the Bpin-based monomer for 30 minutes. The yields increased with temperature in the order −10 °C (70%), 0 °C (80%), and 20 °C (87%), whereas comparable results were obtained for the dispersity (1.14 to 1.20) and molar masses (9.1 to 11.1 kg/mol). The three studies indicate that lower dispersity values are achieved at lower temperatures, while higher yields are obtained at higher temperatures.

The general benefit of lower temperatures for the ideal SCTP behavior is also found upon categorization of the refined data set (Figure 18b). The majority of experiments were performed below 5 °C (227 out of 403) and revealed a high relative contribution of categories I (green, 37%) and II (yellow, 7%). With increasing temperature, their corresponding contribution decreased and shifted toward less controlled polymerizations: Up to 25 °C, 8% belong to category I and 7% to II, whereas up to 45 °C, no results were reported for category I, and in the range of 65 to 85 °C, no results were reported for either category I or II.

#### 5.3.3 The Role of “Hydroxide”

It is generally accepted that the transmetalation step in the Suzuki–Miyaura reaction requires hydroxide ions, i.e., to activate the boronic acid derivatives *via* boronate formation or to form the more reactive Pd hydroxy complex upon halide exchange. Because the polymerization is typically performed in organic/aqueous solvent mixtures (see Chapter 5.3.4), any basic reagent can form equilibria to provide the required hydroxide ions.

##### 5.3.3.1 Type of Base

Inorganic bases can be conveniently utilized to form equilibria due to their basicity, ranging from weak bases (e.g., CsF, K_2_HPO_4_), to moderately basic bases (e.g., Cs_2_CO_3_, K_3_PO_4_) to strongly basic reagents (e.g., LiOH, Bu_4_NOH). In addition, the partition between organic and aqueous phases affects the actual available concentration and can be enhanced by more organo-soluble cations (e.g., Cs^+^ or *tetra*-alkyl ammonium salts vs Na^+^ or K^+^).

The refined data set contains six systematic series (Figure 19a). Dong et al. (series A) compared different carbonates (sodium, potassium, and cesium) and the more basic potassium phosphate applying an *ex situ* prepared initiating catalyst system at 20 °C.^55^ Within the carbonate series, comparable molar masses (7.6 to 10.7 kg/mol) and yields (68 to 95%) were reported but no clear trend with respect to the dispersity values (1.27 to 1.47). The best results were obtained for K_2_CO_3_ (*Đ* = 1.27), which were significantly better compared to the more basic K_3_PO_4_ (*Đ* = 1.67). Huber et al. (series B) compared cesium fluoride to potassium carbonate for a well-defined P­(^*t*^Bu_3_)-based catalyst system at 0 °C.^56^ The usage of CsF revealed only limited SCTP control in terms of the high dispersity (*Đ* = 1.63) and additionally little conversion expressed by a low yield (20%). In contrast, potassium carbonate led to a good yield (60%) and a low dispersity (*Đ* = 1.18).

**Figure 19:**
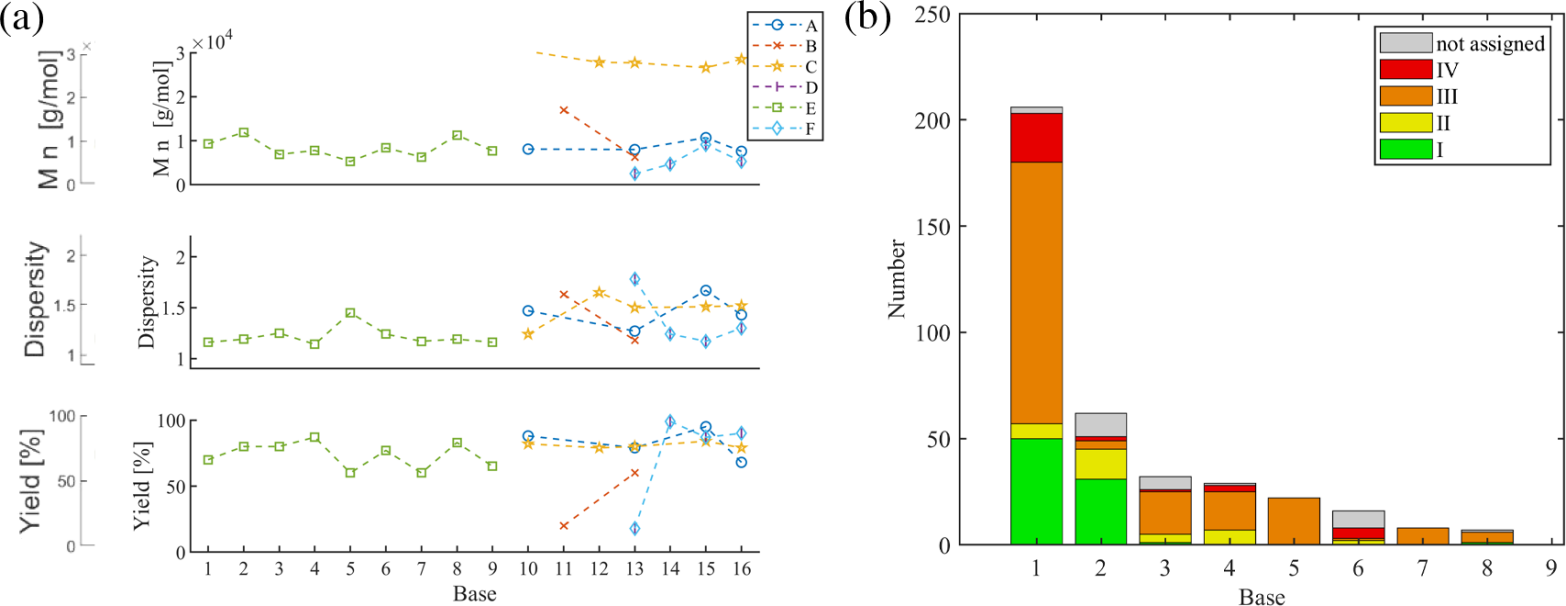
(a) Overview of systematic studies on the effect of the type of base. (b) Categorization of the refined data set by the type of base.

Grisorio et al. (series C) investigated different carbonates (sodium, potassium, and cesium), potassium phosphate, and tetra-ethylammonium hydroxide.^44^ The results were very reproducible in terms of obtained molar masses (26.6 to 30.3 kg/mol) and high yields (79 to 84%). In comparison to the related carbonate-series A, the same reaction temperature (20 °C), same reaction time (30 minutes), and a similar monomer-to-catalyst ratio (20 vs 17) were applied, but it differed markedly in the Pd^2+^ precatalyst that leads to “XM_2_Pd­(L)­X” as the initiating catalyst system. Assuming chain dissociation in the late stage of polymerization, it seems plausible that chain coupling at each α-terminus could occur, explaining the higher obtained molar masses but comparable yields. This hypothesis is corroborated by the high dispersity values (*Đ* ≥ 1.50) except for Cs_2_CO_3_, which was found to be significantly lower (1.20). Zhang et al. reported in 2012 and 2015 identical values for the comparison of dipotassium hydrogen phosphate, sodium and potassium carbonate, and potassium phosphate (series D and F), which are treated as the same result in the following.^51,53^ In comparison to those in the previous series, the results are less clear: For example, the experiment with K_2_CO_3_ revealed unusually low yields (20%) and low molar masses with an exceptionally high dispersity (1.78), whereas Na_2_CO_3_ led to a high yield (90%) and a low dispersity (1.30). Similar polymer characteristics were found for K_2_HPO_4_ (*Đ* = 1.24) and the stronger base K_3_PO_4_ (*Đ* = 1.17). This unusual behavior likely prompted the authors to reproduce and study the interplay of various combinations of Na_2_CO_3_, Cs_2_CO_3_, K_2_CO3, K_3_PO_4_, or K_2_HPO_4_ during the initiation and polymerization stages (series E). Except for one experiment (entry #5), the dispersity values (1.14 to 1.25) and the obtained yields (60 to 87%) were reasonably comparable, confirming the nature of the cation or anion is not inherently limiting for SCTP, neither in the initiation nor polymerization stage. The global analysis (Figure 19b) revealed the extensive use of potassium as the base’s cation (K_3_PO_4_ (1) and K_2_CO_3_ (2)), which also led to the best results in terms of the SCTP classification.

##### 5.3.3.2 Base Equivalents per Monomer

In combination with the type of base, the number of base equivalents, with respect to the monomer, may affect the outcome of the polymerization.

Regarding the systematic series of base equivalents (Figure 20a), Dong et al. tested the range from eight to twenty base equivalents under well-controlled conditions, i.e., applying the P­(Ad)_3_-based Ar–Pd­(L)–X’ initiating catalyst system at 0 °C for a polymerization time of one hour (series A). Using eight equivalents led to lower yield (64%) and molar mass (5.7 kg/mol) than for ≥ ten equivalents (79 to 83%, 6.9 to 8.2 kg/mol), and the dispersity increased slightly using 15 and 20 equivalents (1.16 and 1.20, respectively). However, the trend is only weak. Grisorio et al. compared ten vs twenty equivalents in their Pd^2+^-based precatalyst system (series B). Although the yields were comparable (80% and 81%) and the molar masses were different, somewhat lower dispersity was obtained for ten equiv (*Đ* = 1.19, 27.0 kg/mol) vs twenty equiv (*Đ* = 1.25, 19.9 kg/mol).

**Figure 20:**
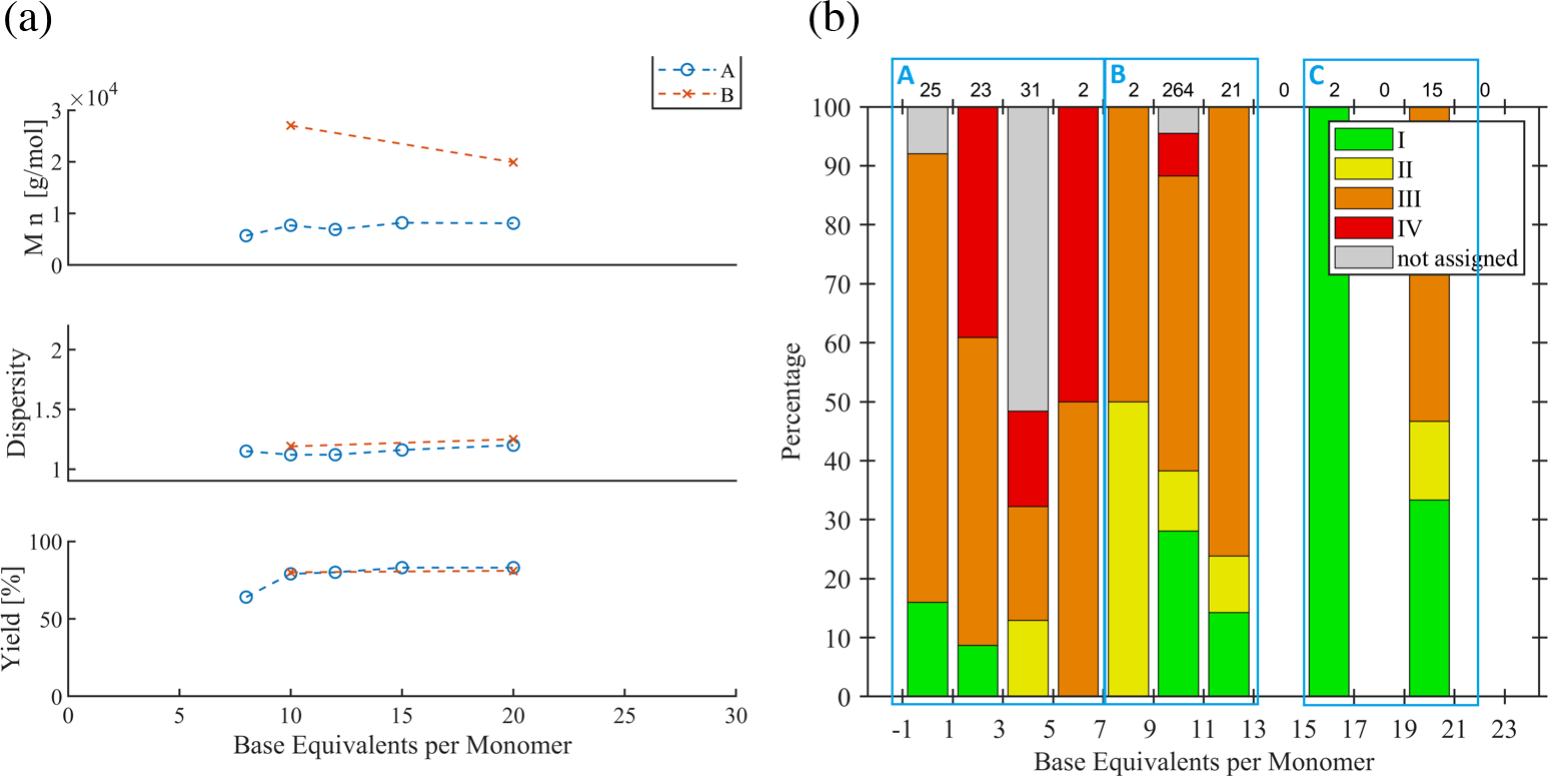
(a) Overview of systematic studies on the effect of the number of equivalents of base per monomer. (b) Categorization of the refined data set by the number of equivalents of base per monomer binned in region A (below 7), B (from 7 to 13), and C (above 15).

The categorization of the refined data set by the number of equivalents of base per monomer is presented in Figure 20b. The relative contributions are binned into specific regions A (below 7), B (7 to 13), and C (above 13), in order to account for the uneven distribution of datapoints. Region A (below 7 equiv) contains in total 81 entries, which reveals limited SCTP fidelity as judged from the sizable contribution of categories III (orange), IV (red), and “not assigned” (gray). The six entries of category I with 0 or 2 equivalents belong to the exceptional series of boronate monomers,^57^ which in fact resemble an already activated substrate for transmetalation. The dominant region B (287 entries) between 7 and 13 equiv visualizes the potential to gain control, i.e., SCTP character, by the enhanced relative contribution of categories I and II, which was also found in region C (17 entries) above 15 equivalents. This finding parallels the results of the two series and suggests that an excess of base is generally beneficial but not required for boronate-based monomers.

##### 5.3.3.3 Water Content

Closely associated with the type and amount of base, the amount of water determines the extent of hydroxide formation for the transmetalation step as well as the hydrolysis of boronic acid derivatives. In the former case, aqueous solutions of inorganic bases are typically used to sustain the required equilibria. A further role of water is to hydrolyze the boronic acid derivative. While Bpin is widely accepted to hydrolyze *in situ* in sufficient amounts, stronger protective groups (e.g., MIDA) may require (more) water for activation, whereas the activated boronate monomers may not need water at all.

Ying et al. reported in 2012 the reproduction with high yields (series A). Two later reports contain data on the systematic variation of the water content within the refined data set. Grisorio et al. tested different volumes of water that account for 277, 555, or 1110 equivalents of water per monomer, applying a Pd^2+^ precatalyst with the P­(^*t*^Bu_3_) ligand and a Br/Bpin-based monomer.^43^ The three series differed only in the monomer-to-catalyst ratio of seven (series B), ten (series C), or twenty (series D). In the case of 227 and 554 equiv, all three series revealed reproducibly high yields (80 to 85%). Furthermore, lower dispersity values (1.16 to 1.19) were observed in the case of 554 equiv, and the molar masses increased with the monomer-to-catalyst ratio as expected. Interestingly, the only datapoint at 1110 equiv showed lower yield (51%) and higher dispersity (1.41).

Howell et al. reported two series. The first series explores the preactivated boronate monomer in the absence of any added base comparing nonaqueous conditions vs the presence of 294 equiv of water (series E). The yield was comparably high (57% and 60%), and the nonaqueous conditions led to smaller dispersity values (1.22 vs 1.35) as well as the related lower molar masses (5.7 kg/mol vs 7.5 kg/mol). The last systematic series investigated the role of water for the MIDA-protected monomer in the range of 88 to 441 equivalents of water (series F), applying the unusual combination of an *ex situ* prepared Ar–Pd­(L)–X’ initiating catalyst system, Cs_2_CO_3_ as base, and Ag_2_SO_4_ as an additive. Nonetheless, the necessity of water for the polymerization within 42 hours was apparent from the increased yields and corresponding molar masses, although the dispersity values were larger at higher yields, illustrating the loss of control toward higher conversion.

The categorization of the refined data set revealed the necessity of water for SCTP (Figure 21b), because the entries belonging to class “I” below 200 equiv were obtained for the boronate species. The majority of the experiments were performed in the range of 200 to 400 equivalents, while a large excess of >600 equivalents is not crucial.

**Figure 21:**
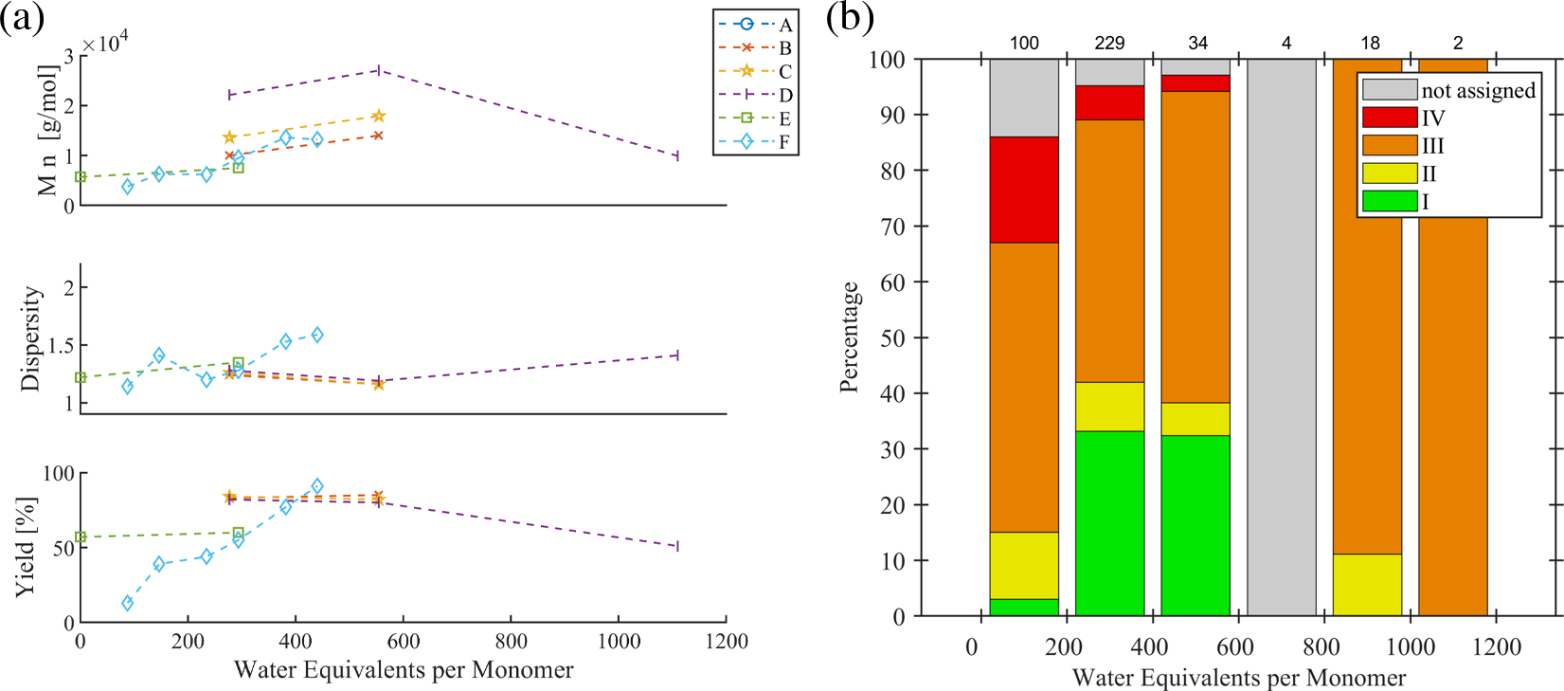
(a) Overview of systematic studies on the effect of the number of equivalents of water per monomer. (b) Categorization of the refined data set by the number of equivalents of water per monomer.

#### 5.3.4 Type of Solvent

In extension of the previous analysis of available hydroxide ions—primarily determined by the type of base, the number of equivalents of base, and the water content—the type of solvent may also affect the outcome of the polymerization. Although the solvent is generally expected to be inert and to govern the available temperature range, sufficient heat and mass transfer as well as solubility of all species should be ensured. Only one series was found in the global data set. Sui et al. utilized a Pd^2+^-based precatalyst and a monomer-to-catalyst ratio of 100 at 20 °C for one hour and compared aqueous THF, aqueous dioxane, and aqueous toluene.^42^ Only in the case of aqueous THF, polymer could be isolated with a yield of 76%, an observed molar mass of 70.4 kg/mol, and a dispersity of 1.63. The high dispersity is expected from the high monomer-to-catalyst ratio (≫25) as well as the Pd^2+^ precatalyst source (*vide supra*). The obtained molar mass agrees surprisingly well with the expected value of 76 kg/mol assuming a dominant SCTP mechanism (see Section 3.4), i.e., as calculated from the reported yield (76%), the empirically found molar mass per repeating unit (1.0 kg/mol), and the applied monomer-to-catalyst ratio (100).

In contrast to related classical Suzuki–Miyaura cross-coupling reactions of monofunctional Ar–X and Ar’–B­(OR)_2_ that successfully utilize various solvent systems, the majority of experiments (>95%) for SCTP were carried out in aqueous THF. Hence, a meaningful analysis of the effect of solvent is precluded.

#### 5.3.5 Concentration of Monomer

The concentration of the reactants is a simple possibility to tune the associated bimolecular rates and, thus, the polymerization outcome. The maximum concentration of monomer is limited by the solubility, but since the polymer solubility is typically lower, the type of side chain (see Section 5.2.1.3), type of solvent (see Section 5.3.4), and/or applied temperature (see Section 5.3.2) impose further constraints on the practicable limits of the concentration regime. A low concentration slows the reaction rate of any bimolecular step. However, no systematic series was found which is plausible as the concentration is often as high as possible and often arises from empirically derived values. The categorization displayed in Figure 22 reveals that standard protocols are frequently applied at a comparable order of magnitude regarding the monomer concentration (up to 180 mmol L^–1^). The majority of experiments were performed below 30 mmol L^–1^, while in the range from 30 to 60 mmol L^–1^, a better SCTP fidelity might be anticipated as judged from the relative contribution of the categories. Still, above 30 mmol L^–1^, only a few experiments are reported, and many results lack characterization data, but the SCTP fidelity seems to decrease. It should be noted that a higher concentration also leads to a higher concentration of terminal products, either the polymer itself or the accumulated halide, which may impact the solubility of reagents, etc. Although of general interest, further analysis is precluded to date due to the lack of data.

**Figure 22:**
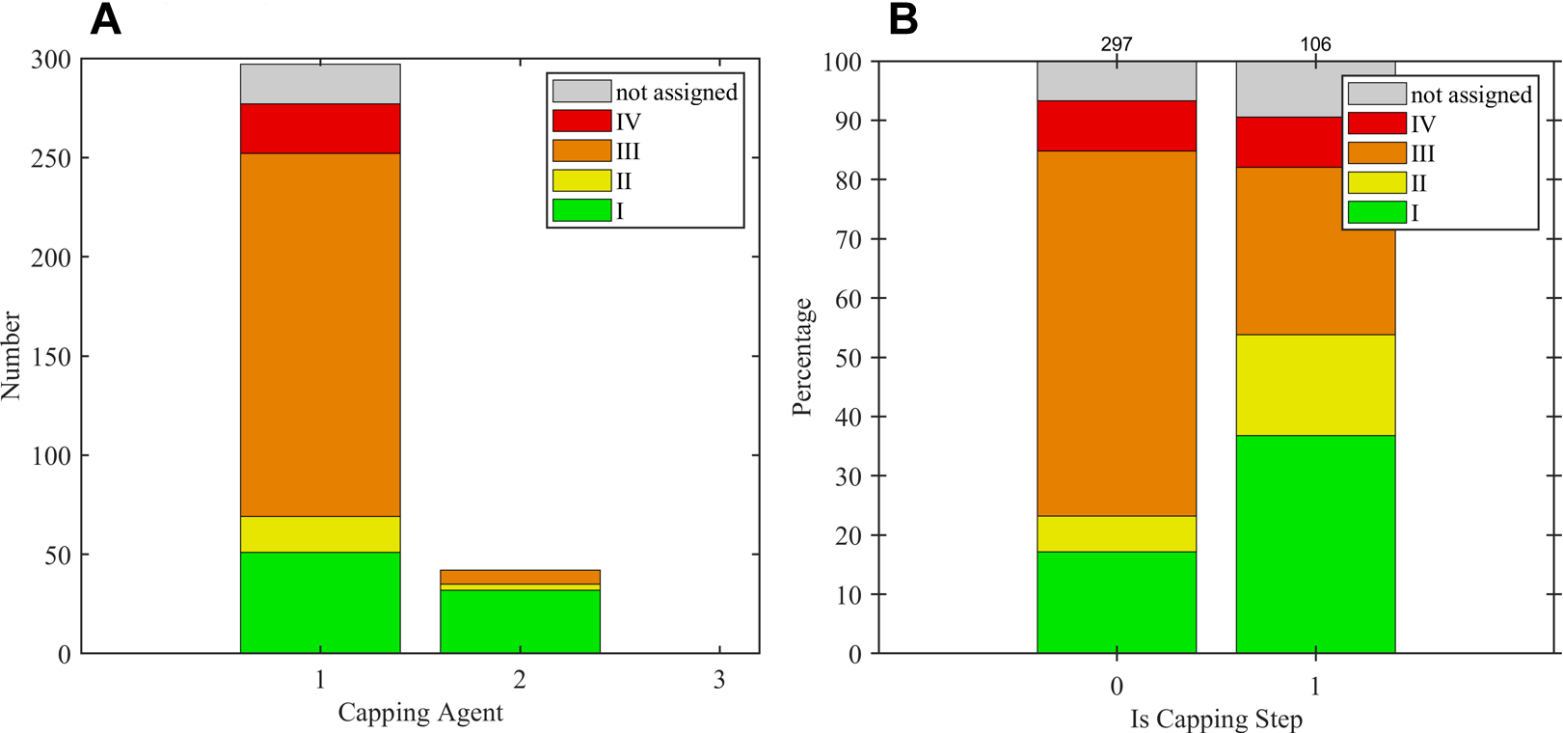
Categorization of the refined data set binned by the monomer concentration.

### 5.4 Capping Stage

The preparation of heterotechelic poly­(fluorene)­s via SCTP relies on the introduction of specific functional end groups. While the α-terminus is controlled in the initiation stage to attach the Ar group, the capping stage enables the introduction of an ω-group using a monofunctional boronic acid (ester) derivative Ar’–B­(OR)_2_. Because the polymerization is typically completed at that stage, the effect of capping is expected to be low on the yield, the obtained molar masses, and the dispersity values. However, if side reactions are severe during the polymerization, sizable amounts of X/[B]-decorated chains may be formed (Section 2.1) that may undergo chain–chain couplings as well as termination by the capping agent. Hence, the following brief analysis may serve to interpret the extent of such side reactions, in particular comparing the absence and the presence of a capping agent.

#### 5.4.1 Capping Agent

The analysis of the refined data set revealed nine systematic series that compared different monoaryl boronic acid derivatives (Ar’–X). As anticipated, the obtained molar masses, the dispersity values, and the yield were comparable and consistent within each series (Figure 23a), also in the absence of a capping agent. The categorization revealed that the majority of experiments (297 of 403) were performed without any terminating reaction (Figure 23b). The usage of a capping agent for the remaining experiments (106) shows a large contribution of categories I and II, which confirms that capping does not inherently limit the SCTP fidelity. It should be noted that the apparent difference with or without capping is likely due to the different conditions because the systematic series show no major effect. In this regard, mass spectrometry provides insights into the capping efficiency, but such a discussion is beyond the scope of this work.

**Figure 23:**
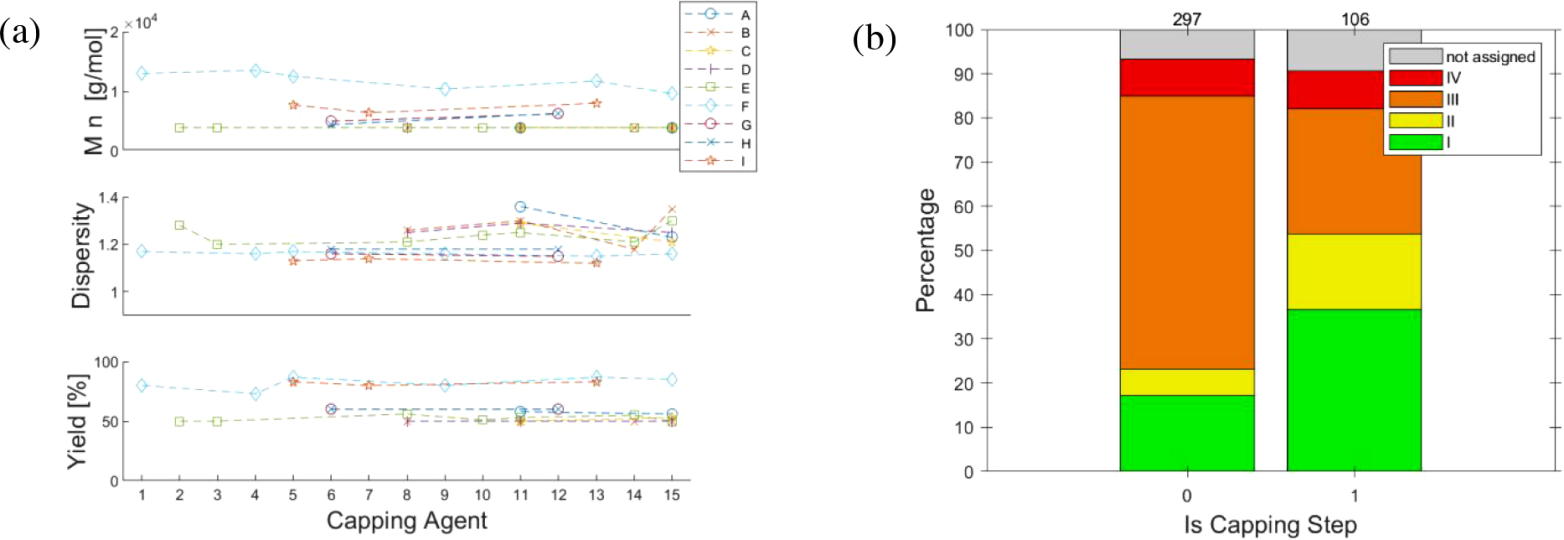
(a) Overview of systematic studies on the effect of the capping agent. (b) Categorization of the refined data set according to the absence (0) or presence (1) of capping agent.

## 6 Global Analysis at Monomer-to-Catalyst Ratio above 25

In the previous section, the analysis was based on the refined data set for experiments using a monomer-to-catalyst ratio below 25. This threshold was obtained from the SCTP categorization of the global data set (see Section 3.3), which ensures that the parameter analysis is not inherently superimposed by experiments with a too high monomer-to-catalyst ratio. Consequently, 84 of 489 experiments were not included in the previous analysis, and the main similarities and differences will be summarized in the following. For this task, monomer-to-catalyst ratios above 25 were applied, as well as a threshold of more than three experiments per reference, in order to prevent too scattered data by the refinement. As a result, five key studies were identified (Table 5).

**Table 5:**
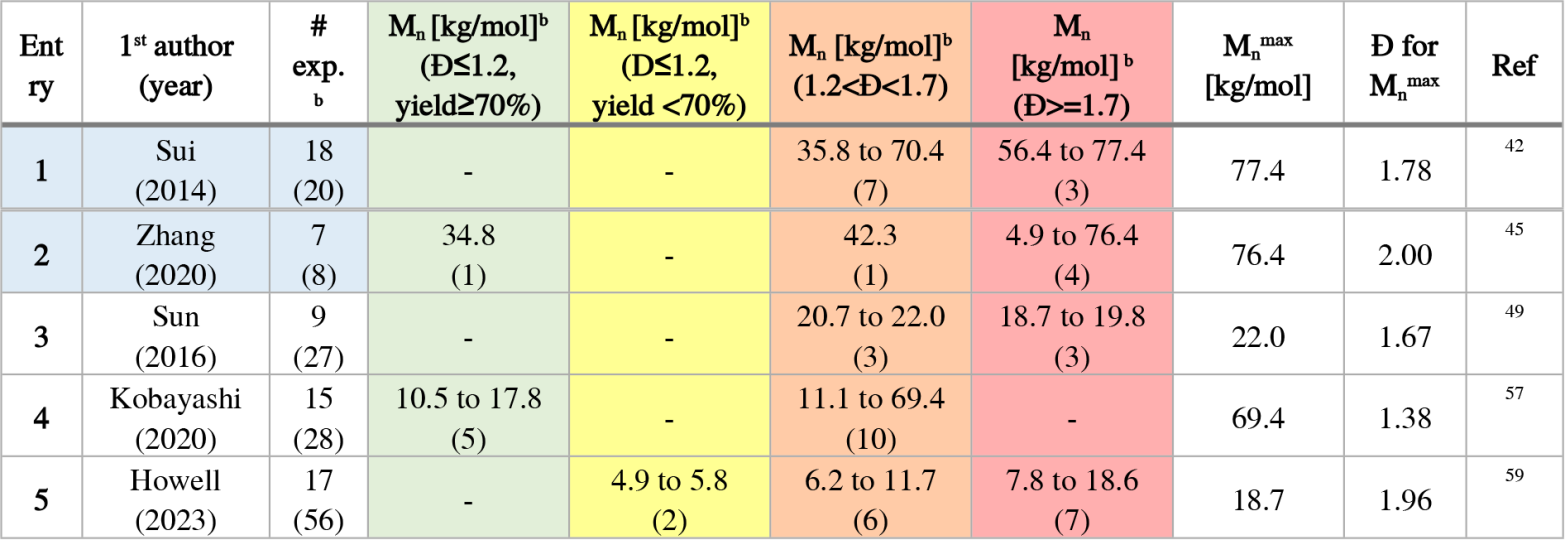
Overview of Studies on AB-Type Suzuki Polycondensation Using a Monomer-to-Catalyst Ratio > 25^a^ ^b^

a Reports with more than three experiments according to their dispersity (Đ) and values for *M*_n_ in kg/mol. Blue-shaded: mainly Pd^II^ precatalysts. Color shadings of *M*_n_ indicate the categorization of results (see text for details).

b Number in parentheses represents the number of experiments belonging to the selection, see Table 4 for full details.

Sui et al. 2014 investigated various Pd^2+^ precatalysts and bases as well as monomer-to-catalyst ratios from 10 to 200.^42^ One systematic series is presented for a carbene-based Pd^2+^ precatalyst using K_3_PO_4_ in aqueous THF at 20 °C for one hour reaction time. With an increasing monomer-to-catalyst ratio, the molar masses increased. For low monomer-to-catalyst ratios, the yield and dispersity were found to be somewhat lower than for the rest of the series, which may arise from the removal of oligomers upon precipitation in methanol. Up to a monomer-to-catalyst ratio of 50, the obtained molar masses increased linearly but leveled off at higher monomer-to-catalyst ratios, implying the formation of new shorter chains, in line with the pathways upon catalyst dissociation.

Zhang et al. 2020 explored Pd­(MeCN)_2_Cl_2_ as the Pd precatalyst in aqueous THF between 20 and 110 °C applying a monomer-to-catalyst ratio of 100. Not unexpectedly, the obtained polymers revealed high molar masses but also high dispersity values of up to 2.00.^45^

Sun et al. reported the variation of the monomer-to-catalyst ratios in the range from 15 to 30 for six systematic series. The authors explored three different *para*-phenylene-based Ar groups for which two *ex situ* prepared Ar–Pd­(L)–Br initiating systems based on the phosphine ligands P­(Xyl)_3_ at 25 °C vs P­(Cy)_3_ at 75 °C were tested in aqueous THF. The polymers were consistently obtained in high yields (80 to 90%), and the dispersity values were found around 1.7 with a slight trend toward higher values at higher monomer-to-catalyst ratios. In each series, the reported molar masses correlated with the monomer-to-catalyst ratio but not as much as expected. For example, the 2-fold monomer-to-catalyst ratio (15 vs 30) resulted in a significant deviation of the obtained (maximum + 40%) vs the expected molar masses, considering the comparable yields and dispersity values that suggest a similar polymerization behavior. The variations of the temperature were assigned to the role of the precatalysts, which were reported as unimer or dimer complexes, but no major difference was found in the obtained polymer properties.

Kobayashi et al. explored the exceptional boronate-based monomer in monomer-to-catalyst ratios from 25 to 450 using an Ar–Pd­(L)–Br initiating system based on P­(^*t*^Bu)_3_ at −10 °C for 15 minutes. The results were outstanding, because the yields were consistently high (83 to 89%), the dispersity values were low and increased steadily from 1.14 to only 1.38, and the obtained molar masses revealed a linear growth throughout the series without leveling as previously observed by Sui et al. The major difference stems from the negatively charged boronate-monomer, which reduces the effect of starting new chains by oxidative addition into the monomer due to their electron-donating effect. This is in line with the well accepted effect that implies electron-donating groups reduce the rate constant of oxidative addition, while electron-withdrawing ones enhance the rate constant.

Howell et al. 2023 recently examined the role of the MIDA-protected monomer and its potential for SCTP employing nonconventional polymerization conditions, i.e., an Ar–Pd­(L)–I initiating catalyst system based on the RuPhos phosphine, Ag_2_SO_4_ as an additive to activate the MIDA group, and Cs_2_CO_3_ as a base in aqueous THF at 30 °C. Long reaction times were applied, i.e., two days and three days, but the obtained polymer properties revealed no clear trend. The yields were in the range of 2 to 74%, and the dispersity values ranged from as low as 1.11 up to 1.96 for the same monomer-to-catalyst ratio of 32.

The recent studies by Kobayashi et al. and Howell et al. represent modern approaches to extend the scope of SCTP beyond small monomer-to-catalyst ratios by targeting the reactivity of the monomer (boronate- and MIDA-protected derivatives). These initial results demonstrate the necessity of further exploring promising catalytic conditions, in particular, the role of the catalytic system. Notably, high molar masses can be reached with significantly lower dispersity values at high isolated yields, which illustrates the potential to pursue promising future materials with enhanced properties.

## 7 Summary and Outlook

The literature-reported data on the AB-type Suzuki polycondensation of 2,7-substituted fluorene monomers were retrieved and analyzed in detail in order to evaluate the catalyst-transfer fidelity (SCTP) and the impact of various polymerization parameters.

Table 6 assigns each parameter to four condensed qualitative families, according to the derived strength of correlation with the *M*_n_, *Đ*, and yield from the systematic series, and their agreement within the global data. For this task, the reported yields, molar masses, and dispersity values of the poly­(fluorene)­s were used for categorization in terms of the extent of underlying chain-growth or step-growth contributions. Efficient chain-growth is expected for true SCTP and leads to low dispersity values and predictable molar masses, i.e., defined as category “I” with high reported yields and category “II” upon incomplete conversion. It was found that this categorization correlated with the monomer-to-catalyst ratio and the choice of active initiating catalytic species as the two main parameters. Notably, the majority of categories I and II were observed for a monomer-to-catalyst ratio ≤ 25, similar to a recent finding by the Nesterov group for the B-MIDA-based monomer (MIDA is *N*-methyliminodiacetic acid).^23^ Monomer-to-catalyst ratios above 25 were less frequently reported for standard monomers based on bromide and boronic acid pinacol ester. The analysis is based on (i) the comparison of systematic series, in which only the parameter of interest varies, while all others remain identical, and (ii) the analysis of the categorization of the parameter comprising all experiments to draw more generalized conclusions. All reported and calculated data are provided in Supporting Information to enable and to encourage reinspection, machine learning, and future AI-based analyses.

**Table 6:** Impact of Various Parameters on the SCTP Fidelity of Poly­(fluorenes) Achieved by AB-Type Suzuki Polycondensation

High influence	Medium influence	Low influence	Inferior influence
•Monomer-to-catalyst ratio	•Monomer halide	•Way of active catalyst formation	•Base equivalents
•Initiating catalytic species	•Monomer boronic acid derivative		•L/Pd ratio
	•Ligand		•Monomer side chain
	•Initiator halide	•Concentration	•H_2_O amount
	•Reaction time	•Initiator-to-catalyst ratio	
	•Temperature		
	•Base		

The initiating catalytic system is typically formed by a precatalyst and a monoaryl halide in given stoichiometries. Most of the studies applied such conditions to achieve high SCTP fidelity. If not, side reactions at the initiating stage may allow for later step-growth reactions. The polymerization stage is governed by the monomer, the nature of the active catalyst, and the reaction conditions. The choice of monomeric substructures (halide and boron moieties), ligand, base, temperature, and time revealed their effect on the polymerization behavior to some extent. The capping stage leads only to marginal differences in terms of molar masses, dispersity values, and yields but is important to fully exploit the potential of SCTP in terms of heterotelechelic building blocks. The way of initiator formation, the concentration, and the aryl halide-to-catalyst ratio revealed a moderate influence on the polymerization, while the amount of base and ligand, the chosen solubilizing chain, and the amount of water (if present at all) are found to play an inferior role.

The key finding of this study revealed the isolated yet exceptional results to extend the SCTP fidelity beyond the ubiquitously found limit of monomer-to-catalyst ratios of 25. This was found for boronic acid trimethylol ethane ester salt ([B­(OCH_2_)_3_CCH_3_]^−^), which demonstrated high control over the polymerization for monomer-to-catalyst ratios up to 450 (*M*_n_ = 69.4 kg/mol, *Đ* = 1.38, 89%).^57^ This work is expected to fuel rapid progress in view of the optimization of other parameters and also for other polymer families.

During the analysis, it became clear that the multiparameter interdependencies would exceed the scope of this work, though the methodology deserves an adequate and reproducible presentation. Hence, a general methodological approach to assess and, to the extent possible, systematically work on SCTP is provided. The relevant data for reinspection are tabulated in Supporting Information for the application-oriented as well as the ground-laying work. In addition, the methodology herein is currently being extended to other AB-type monomers for SCTP to gain more general insights into the scope and limitations of the current protocols. On the basis of the collected FAIR data from SEC-retrieved polymer information in this work, the easy access to self-evaluate the specific tabulated data is expected to fuel a controversial and creative development of SCTP.

## Supplementary Material





## Data Availability

The supplementary results are available in Supporting Information. Data supporting this study will be provided upon request.

## Notes

Tabulated data of the global data set (as xlsx-files); all graphics (png-files) and tabulated data (xlsx-files) are provided in a readable archive file, i.e., named by the “series” or “categorization” followed by the name of the parameter (ZIP)
Essentials of Suzuki–Miyaura cross-coupling towards polymerization; application-oriented experiments (PDF)

## #

Fraunhofer Institute for Applied Polymer Research IAP, Research Division Polymeric Materials and Composites PYCO, Schmiedestraße 5, 15745 Wildau, Germany
