# Supplementary figures and images for: Global Analysis of Polyfluorene via AB-Type Suzuki–Miyaura Polymerization: Empirical and Mechanistic Rationalization of Structural and Reaction Parameters on Molar Mass, Dispersity, and Yield
Source: ACS Polym Au. 2025 Dec 22;6(1):6–32. doi: 10.1021/acspolymersau.5c00121 (PMC12903433; doi:10.1021/acspolymersau.5c00121)

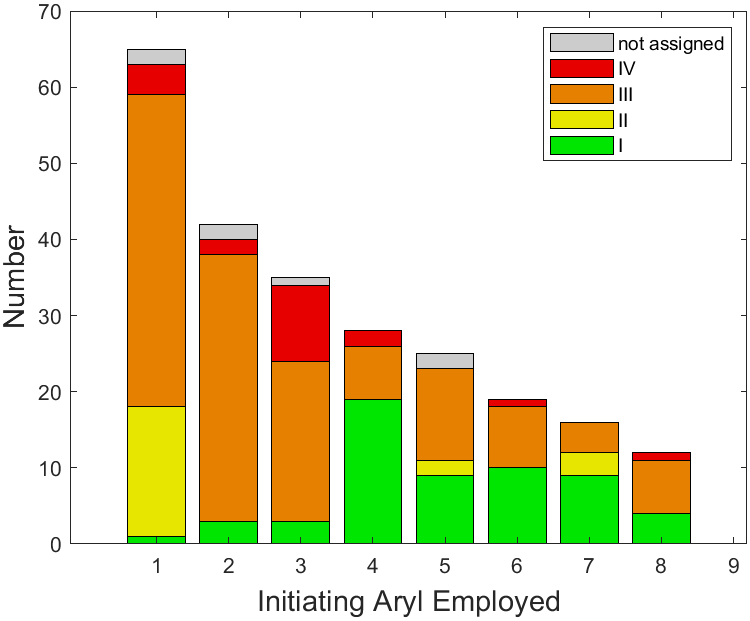

Supplement: Supplementary file 1 [file lg5c00121_si_001.zip › 03_categorization_Initiating Aryl Employed.png]

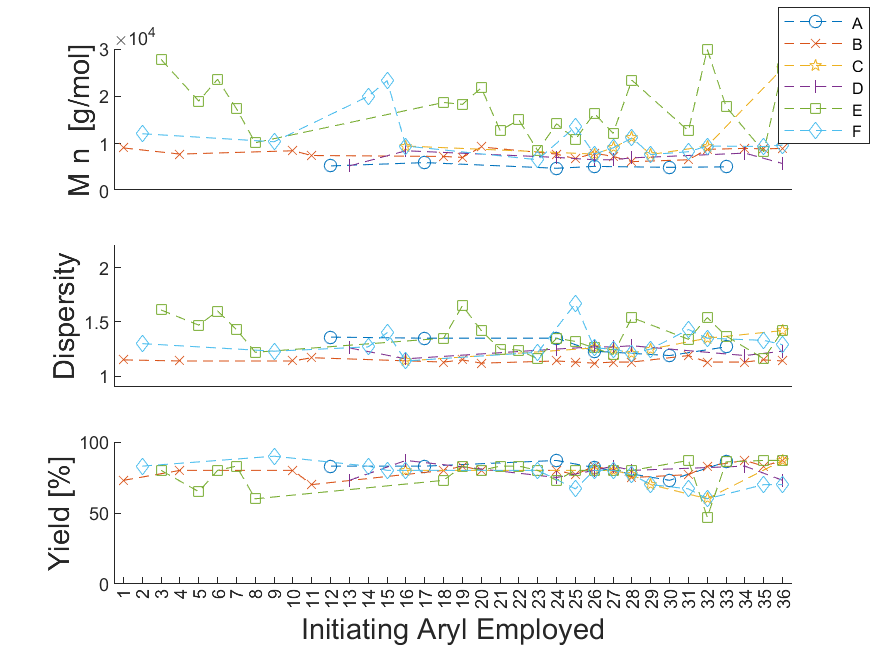

Supplement: Supplementary file 1 [file lg5c00121_si_001.zip › 03_series_Initiating Aryl Employed.png]

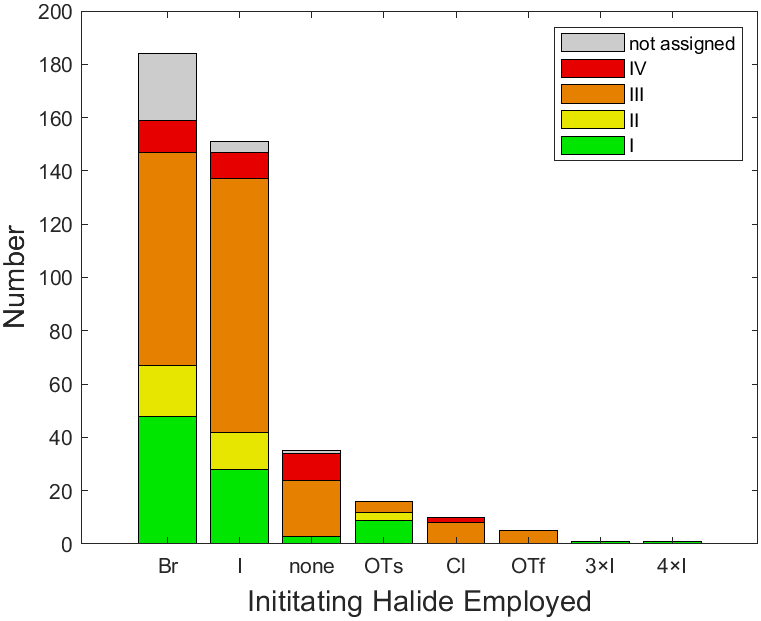

Supplement: Supplementary file 1 [file lg5c00121_si_001.zip › 04_categorization_Inititating Halide Employed.png]

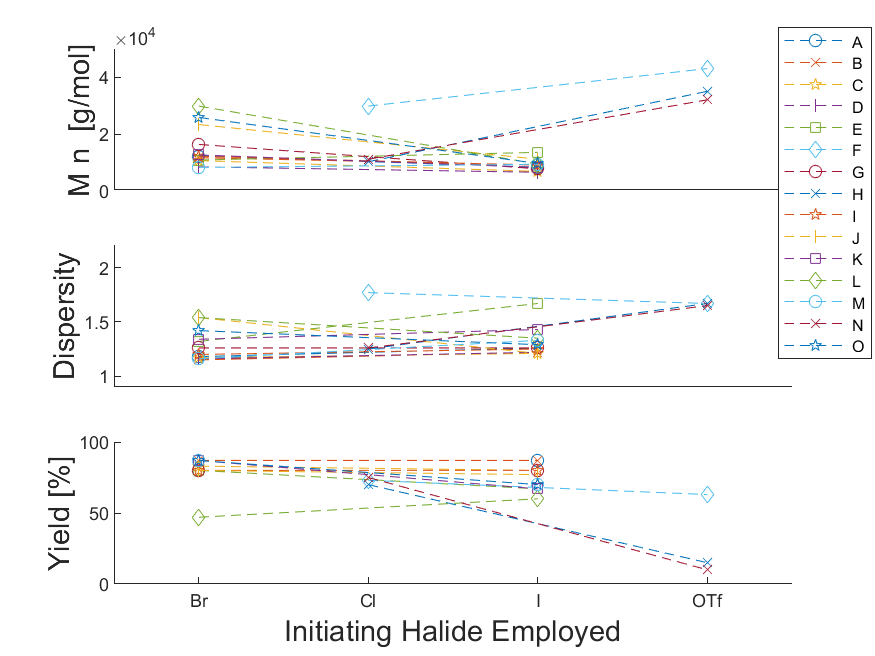

Supplement: Supplementary file 1 [file lg5c00121_si_001.zip › 04_series_Initiating Halide Employed.png]

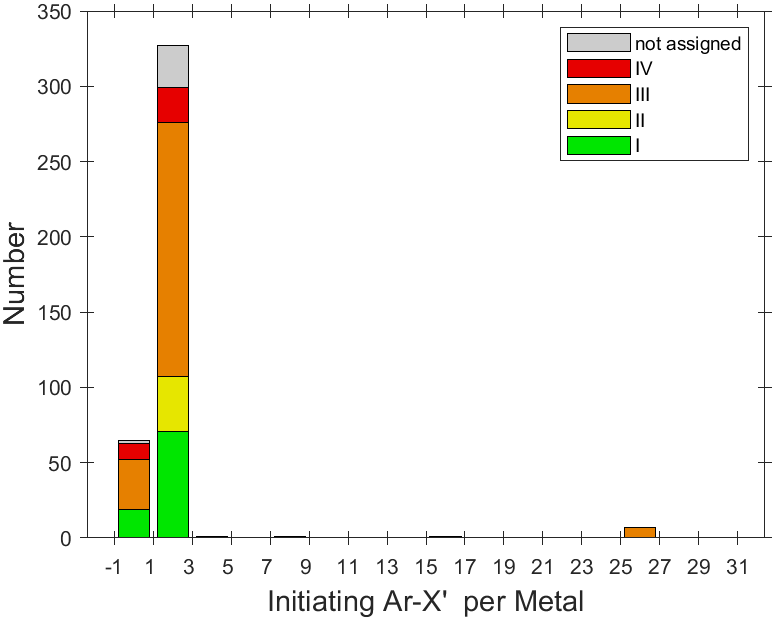

Supplement: Supplementary file 1 [file lg5c00121_si_001.zip › 05_categorization_Initiating Ar-X' Per Metal.png]

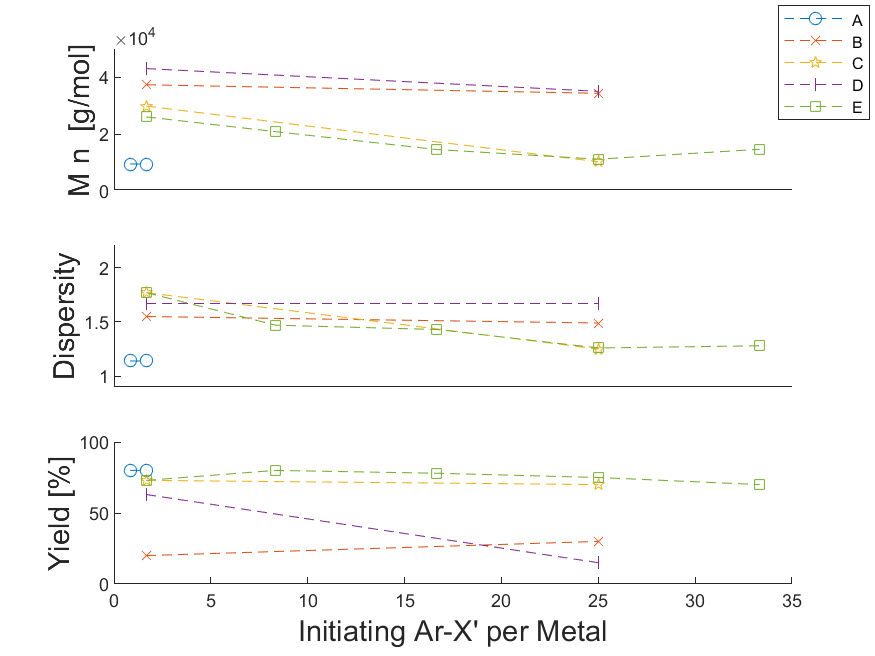

Supplement: Supplementary file 1 [file lg5c00121_si_001.zip › 05_series_Initiating Ar-X' Per Metal.png]

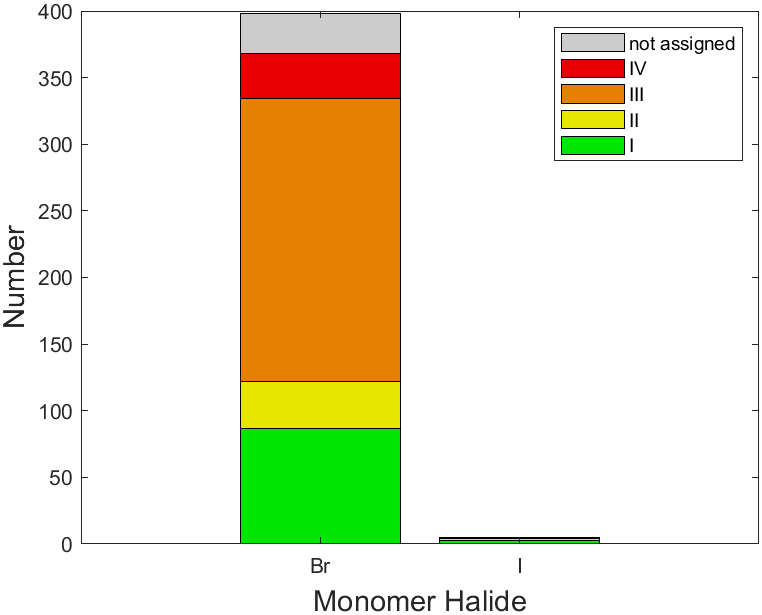

Supplement: Supplementary file 1 [file lg5c00121_si_001.zip › 06_categorization_Monomer Halide.png]

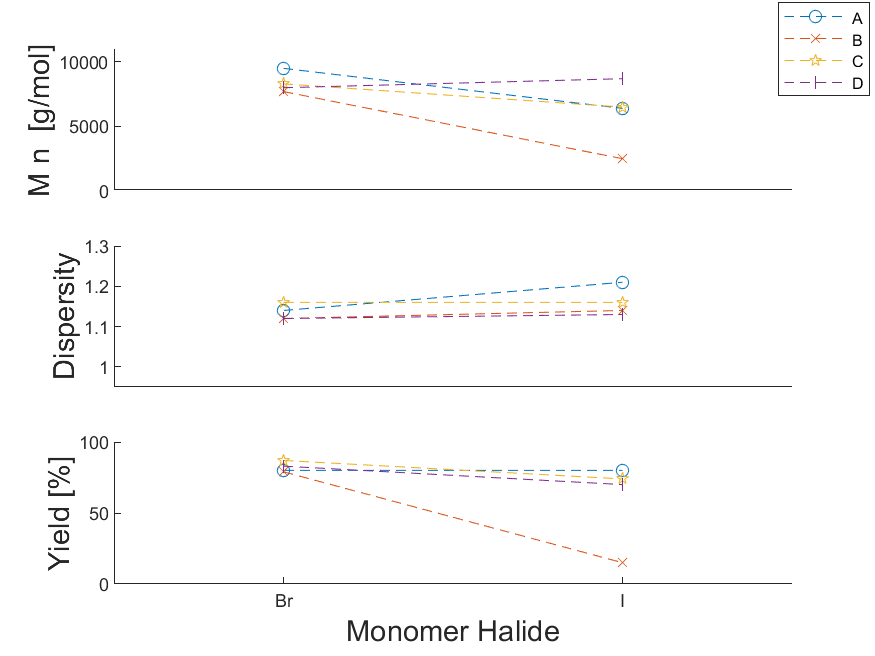

Supplement: Supplementary file 1 [file lg5c00121_si_001.zip › 06_series_Monomer Halide.png]

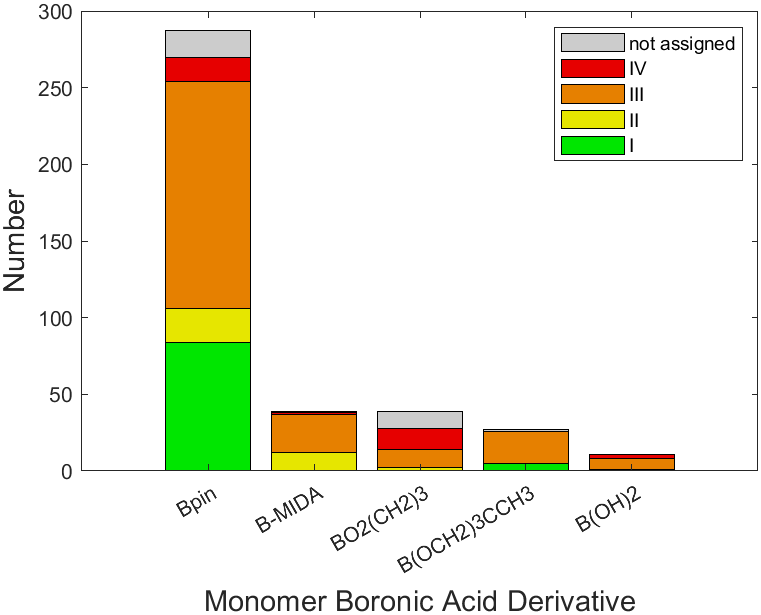

Supplement: Supplementary file 1 [file lg5c00121_si_001.zip › 07_categorization_Monomer Boronic Acid Derivative.png]

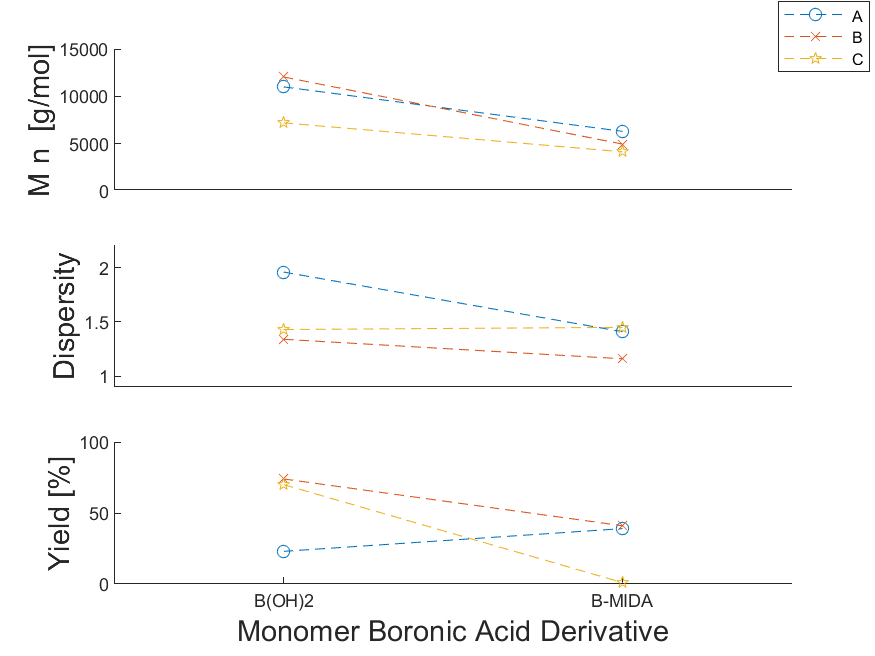

Supplement: Supplementary file 1 [file lg5c00121_si_001.zip › 07_series_Monomer Boronic Acid Derivative.png]

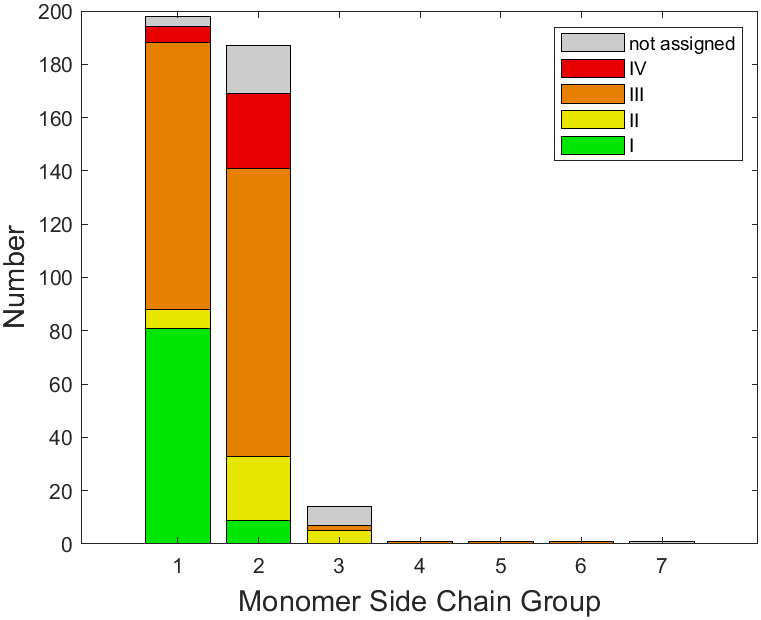

Supplement: Supplementary file 1 [file lg5c00121_si_001.zip › 08_categorization_Monomer Side Chain Group.png]

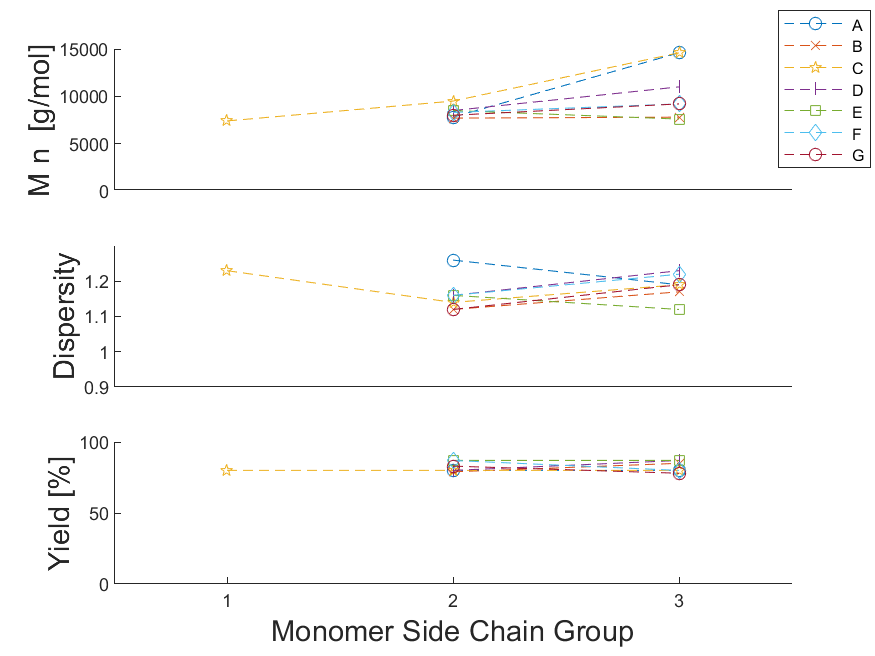

Supplement: Supplementary file 1 [file lg5c00121_si_001.zip › 08_series_Monomer Side Chain Group.png]

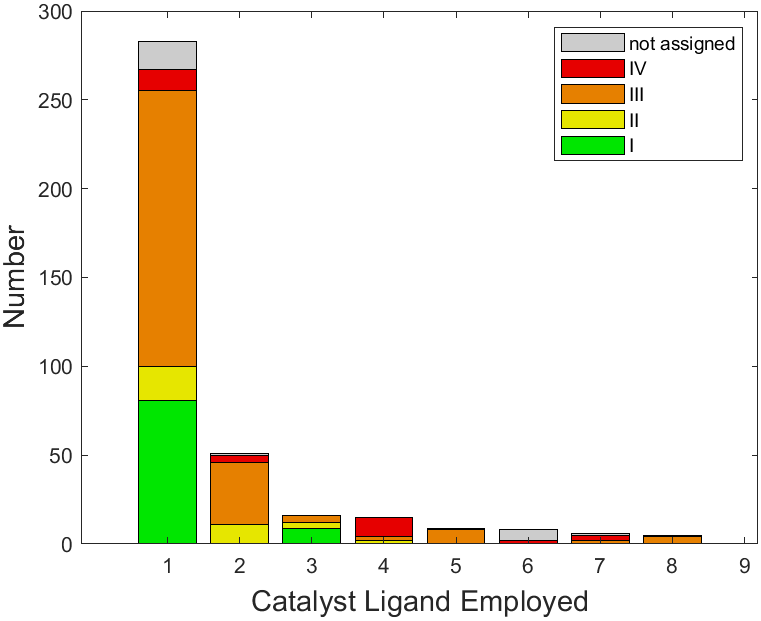

Supplement: Supplementary file 1 [file lg5c00121_si_001.zip › 09_categorization_Catalyst Ligand Employed.png]

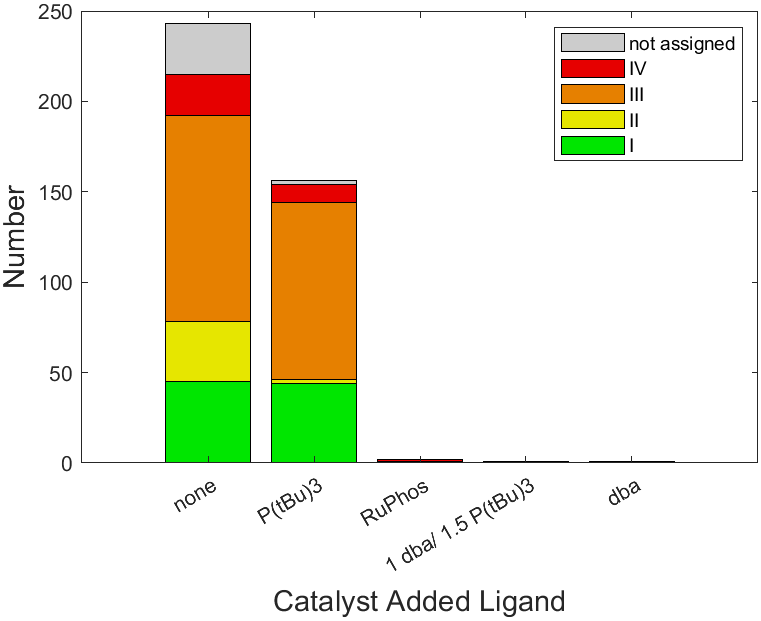

Supplement: Supplementary file 1 [file lg5c00121_si_001.zip › 10_categorization_Catalyst Added Ligand.png]

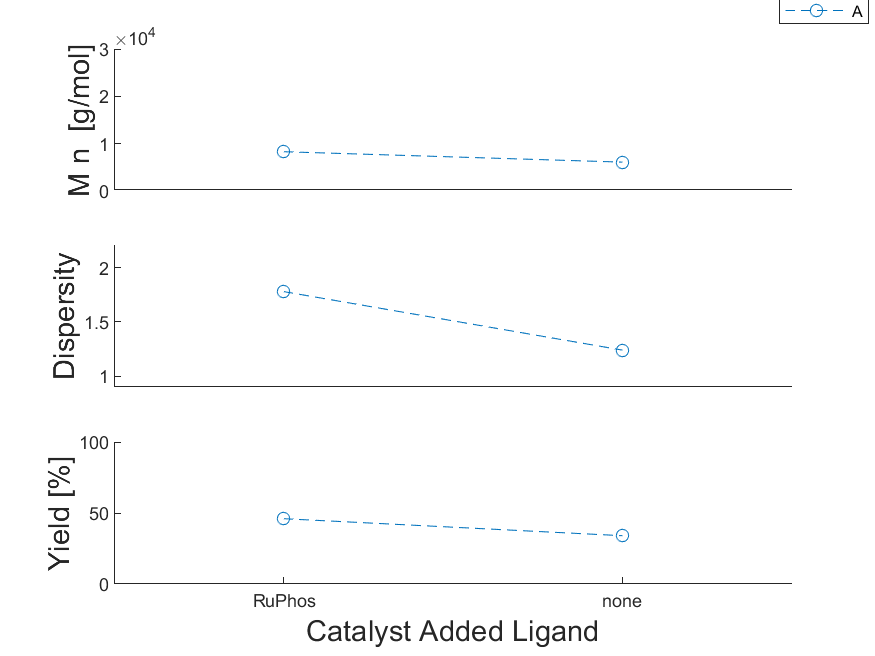

Supplement: Supplementary file 1 [file lg5c00121_si_001.zip › 10_series_Catalyst Added Ligand.png]

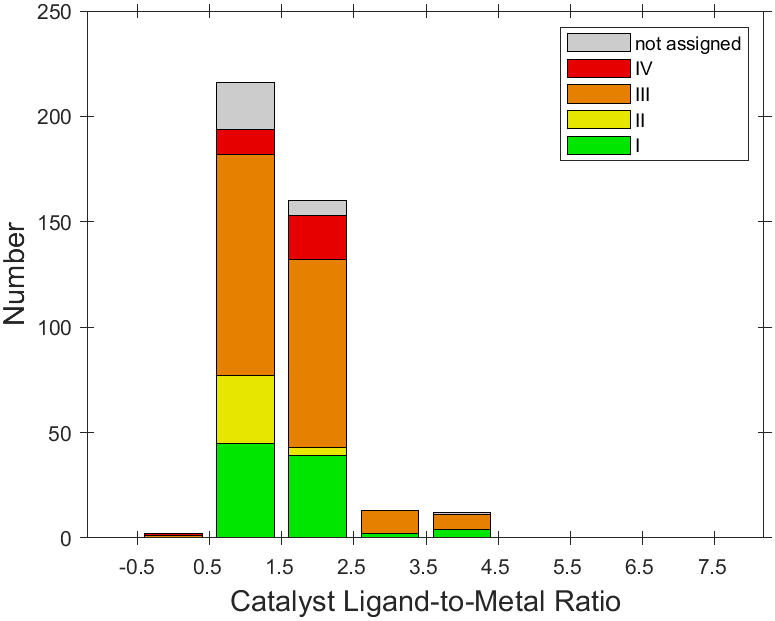

Supplement: Supplementary file 1 [file lg5c00121_si_001.zip › 11_categorization_Catalyst Ligand-to-Metal Ratio.png]

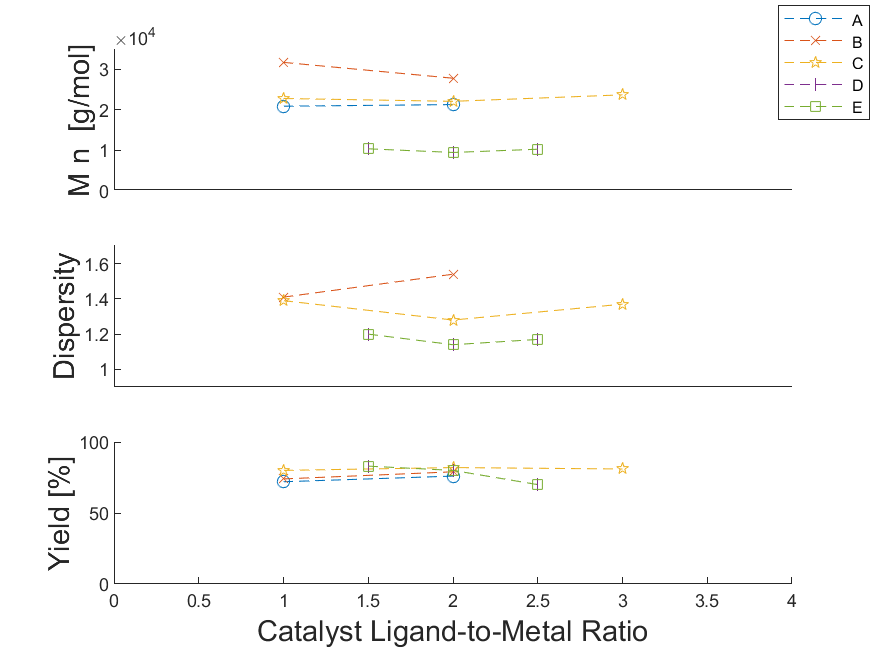

Supplement: Supplementary file 1 [file lg5c00121_si_001.zip › 11_series_Catalyst Ligand-to-Metal Ratio.png]

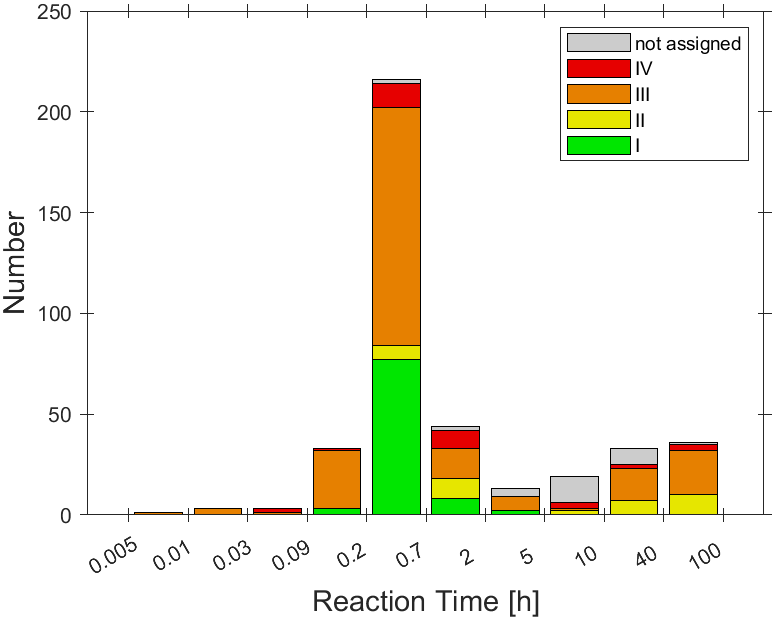

Supplement: Supplementary file 1 [file lg5c00121_si_001.zip › 12_categorization_Reaction Time.png]

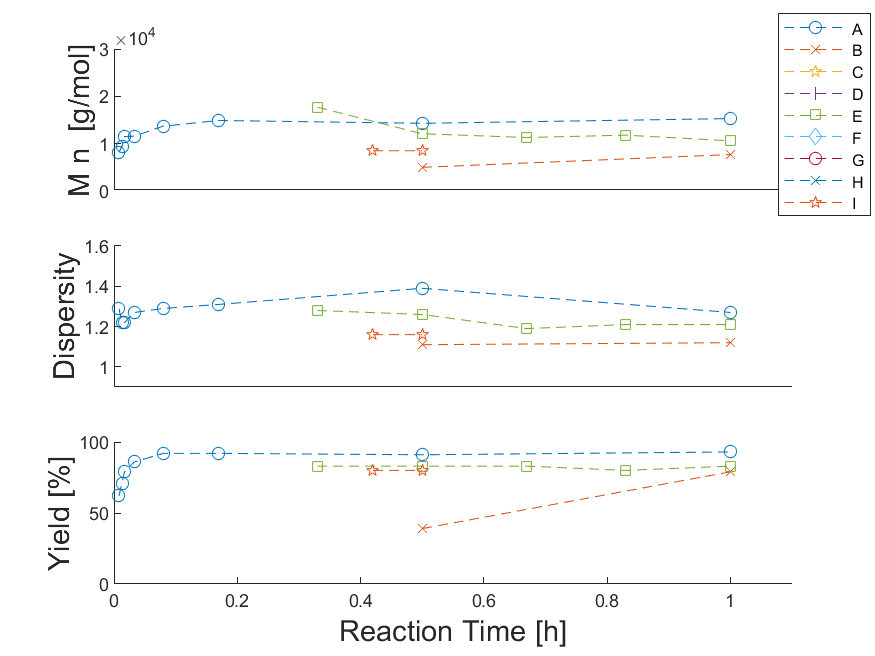

Supplement: Supplementary file 1 [file lg5c00121_si_001.zip › 12_series_Reaction Time [h].png]

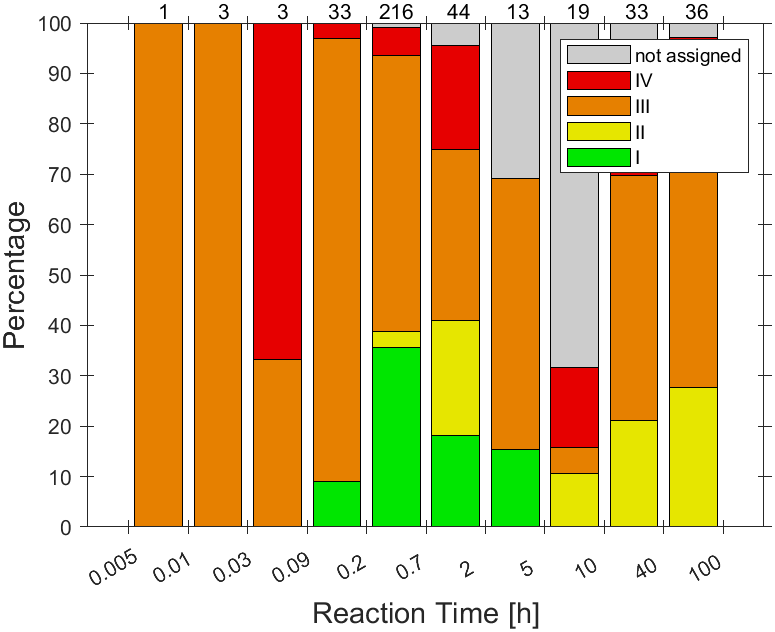

Supplement: Supplementary file 1 [file lg5c00121_si_001.zip › 13_categorization_Reaction Time.png]

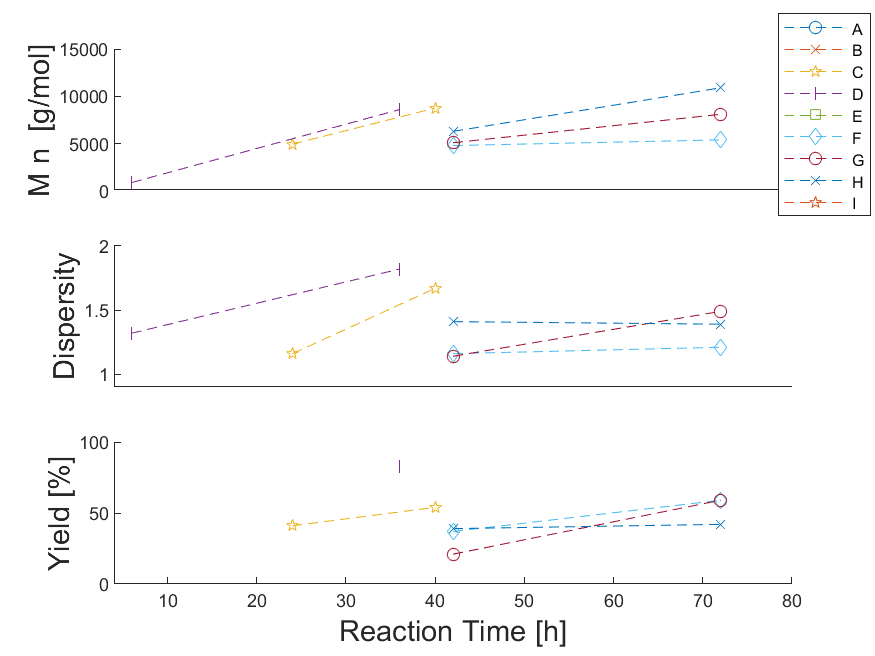

Supplement: Supplementary file 1 [file lg5c00121_si_001.zip › 13_series_Reaction Time [h].png]

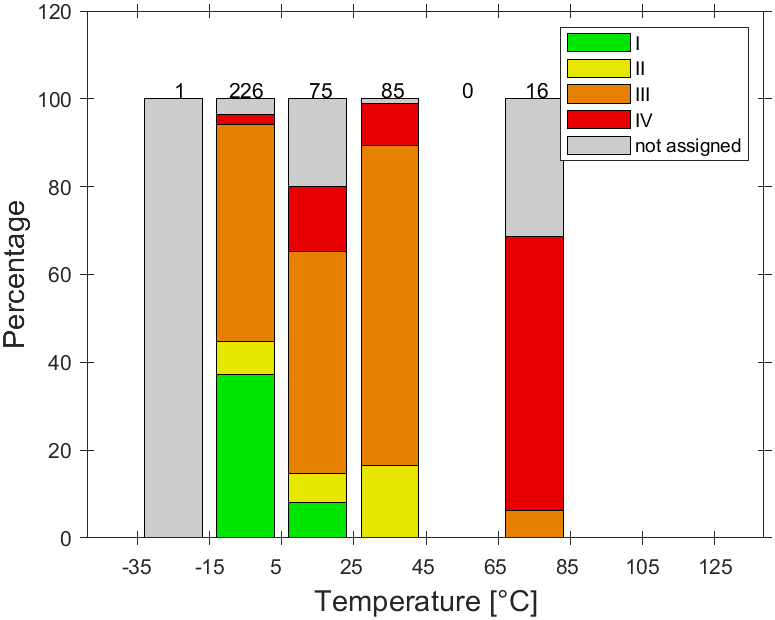

Supplement: Supplementary file 1 [file lg5c00121_si_001.zip › 14_categorization_Temperature.png]

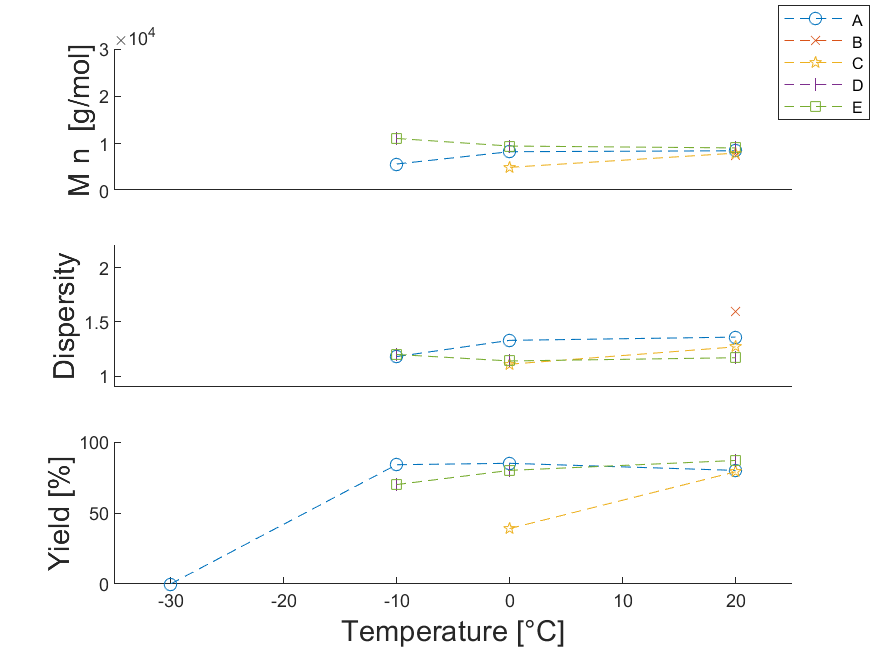

Supplement: Supplementary file 1 [file lg5c00121_si_001.zip › 14_series_Temperature [°C].png]

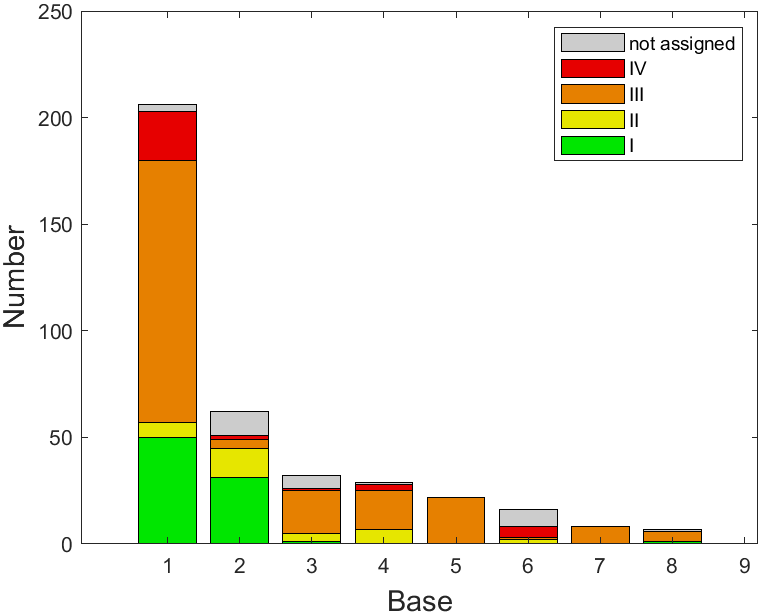

Supplement: Supplementary file 1 [file lg5c00121_si_001.zip › 15_categorization_Base.png]

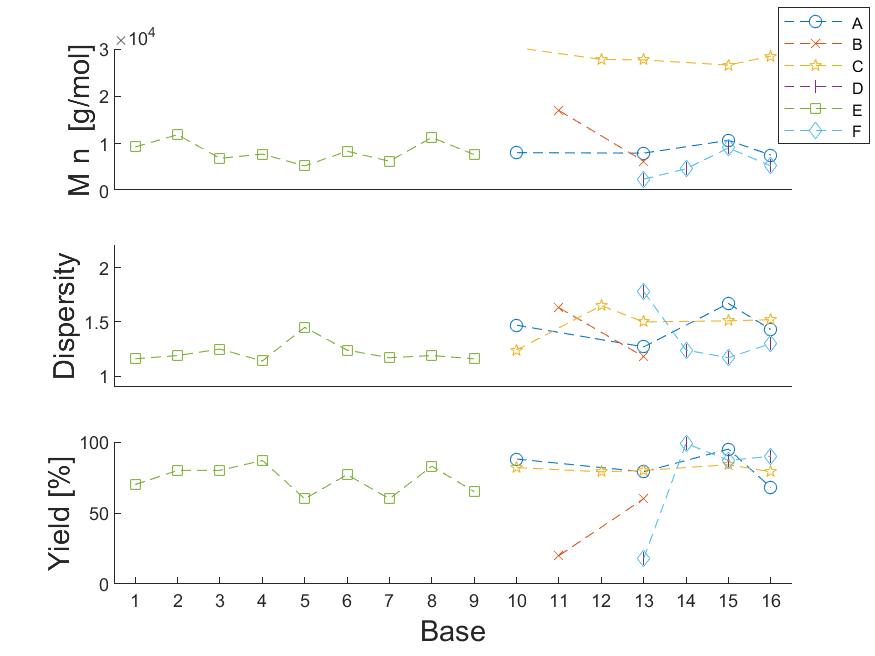

Supplement: Supplementary file 1 [file lg5c00121_si_001.zip › 15_series_Base.png]

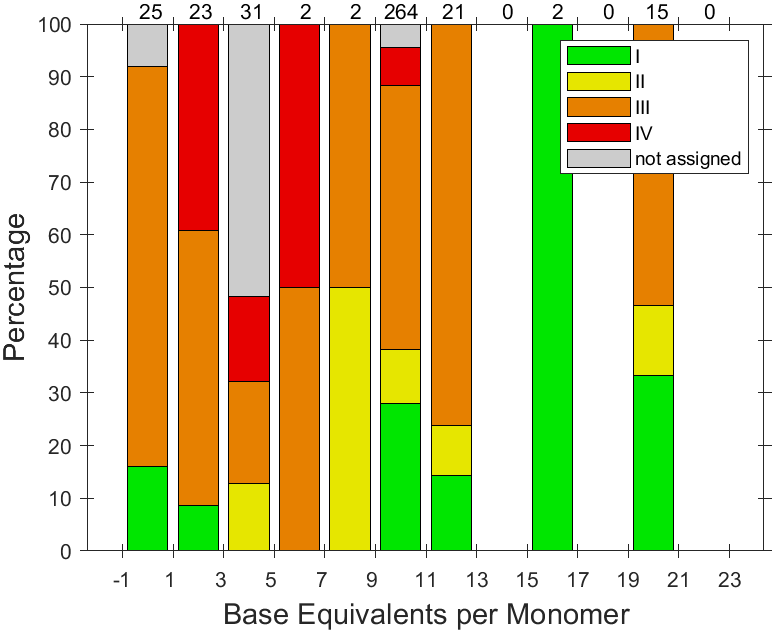

Supplement: Supplementary file 1 [file lg5c00121_si_001.zip › 16_categorization_Base Equivalents Per Monomer.png]

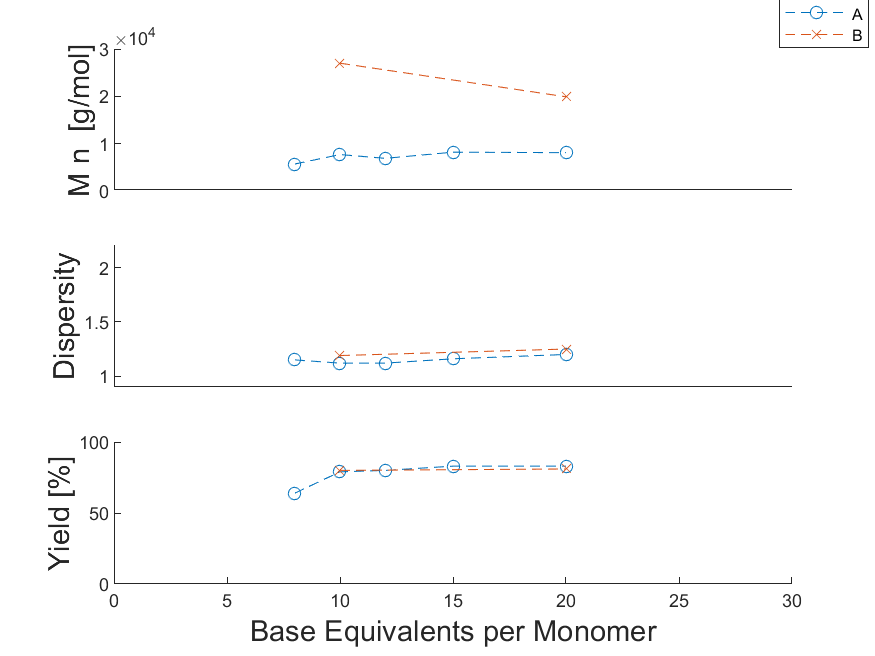

Supplement: Supplementary file 1 [file lg5c00121_si_001.zip › 16_series_Base Equivalents Per Monomer.png]

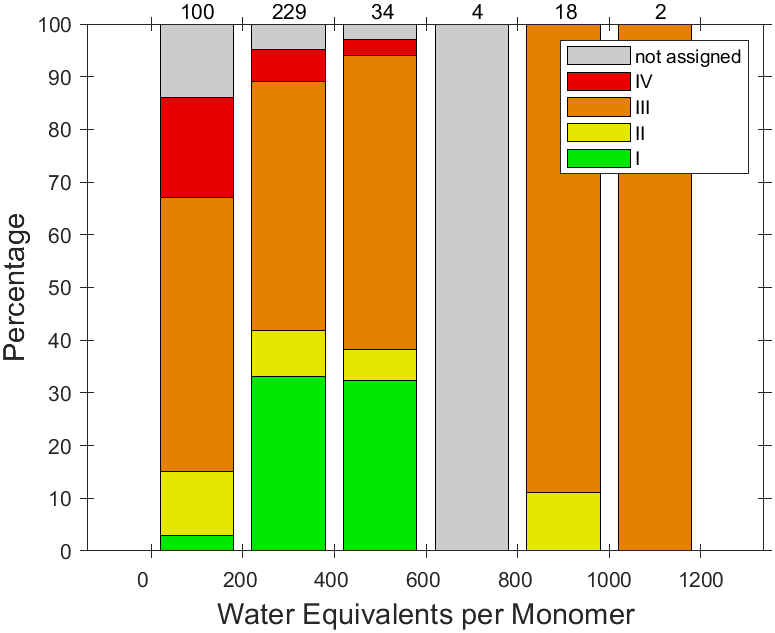

Supplement: Supplementary file 1 [file lg5c00121_si_001.zip › 17_categorization_Water Equivalents Per Monomer.png]

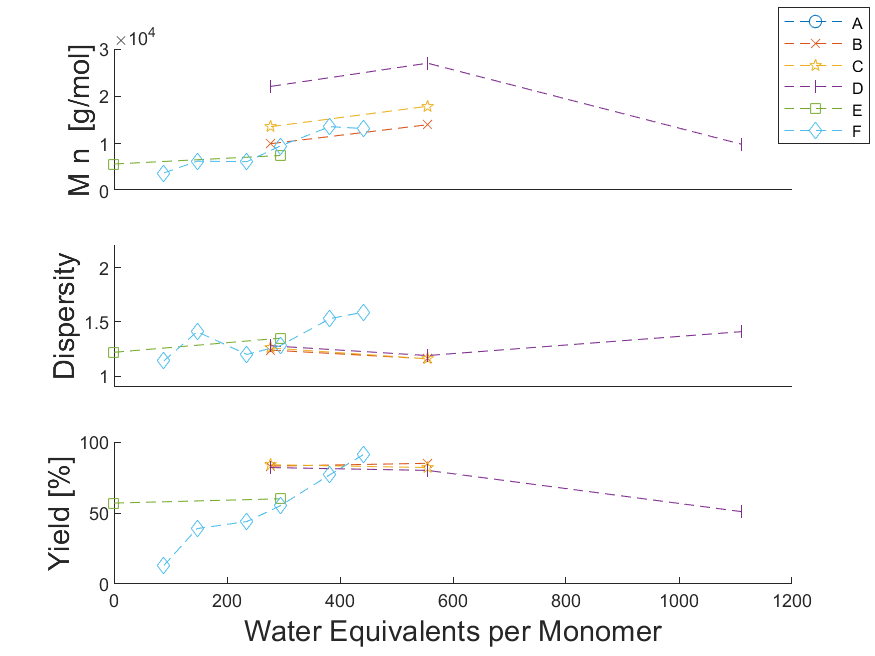

Supplement: Supplementary file 1 [file lg5c00121_si_001.zip › 17_series_Water Equivalents Per Monomer.png]

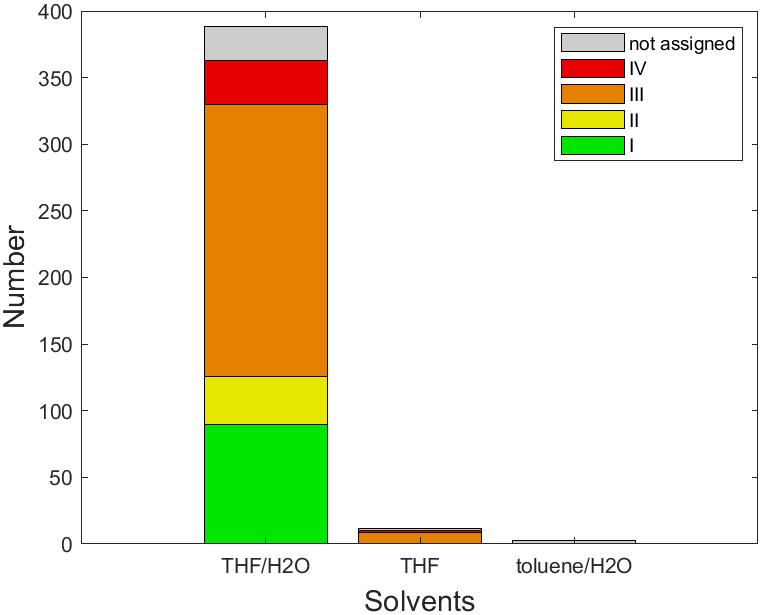

Supplement: Supplementary file 1 [file lg5c00121_si_001.zip › 18_categorization_Solvents.png]

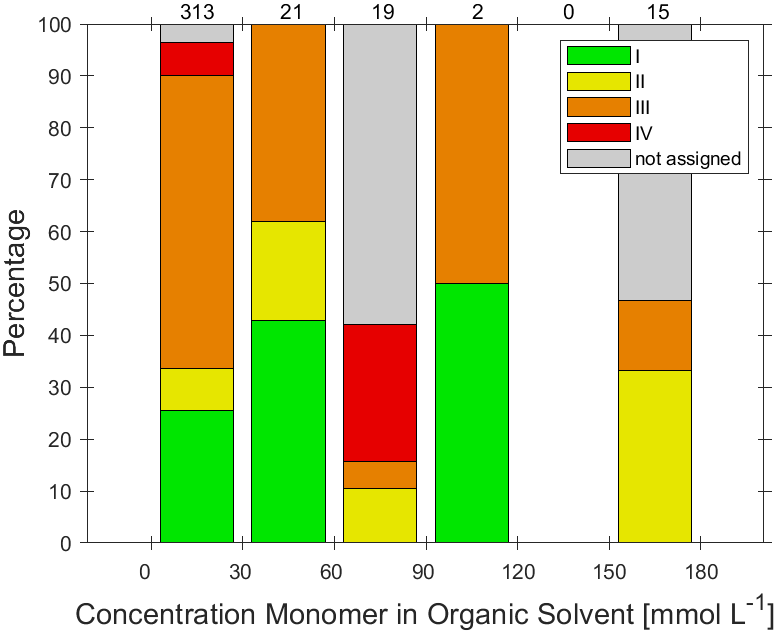

Supplement: Supplementary file 1 [file lg5c00121_si_001.zip › 19_categorization_Concentration Monomer In Organic Solvent.png]

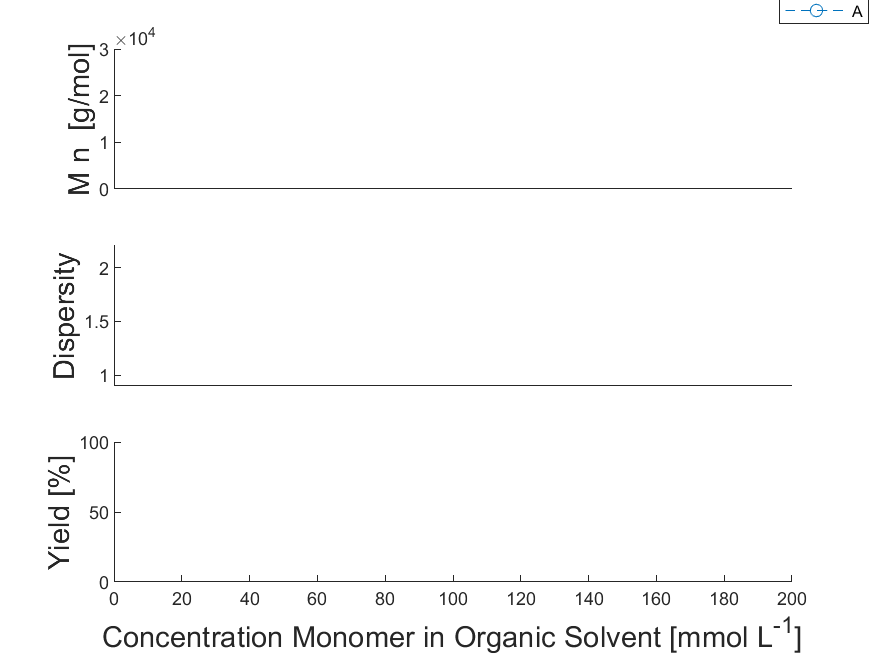

Supplement: Supplementary file 1 [file lg5c00121_si_001.zip › 19_series_Concentration Monomer In Organic Solvent [mmol L^{-1}].png]

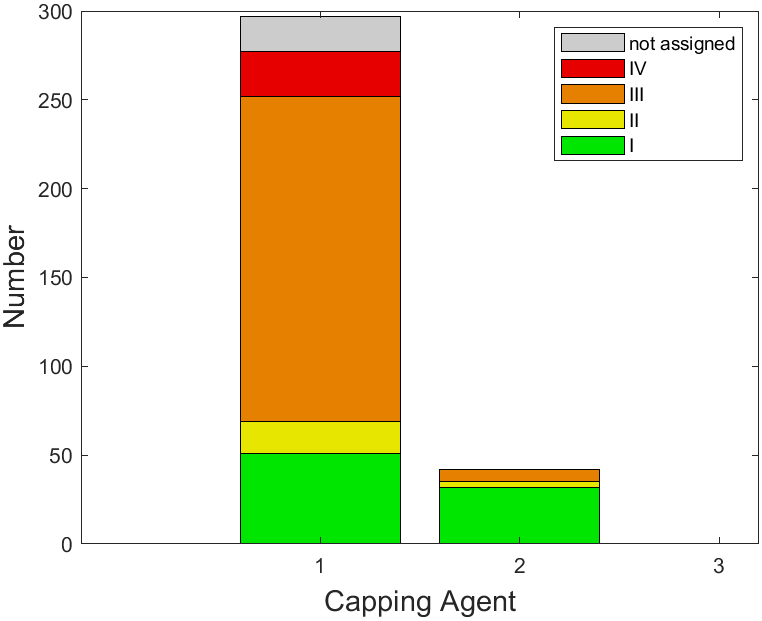

Supplement: Supplementary file 1 [file lg5c00121_si_001.zip › 20_categorization_Capping Agent.png]

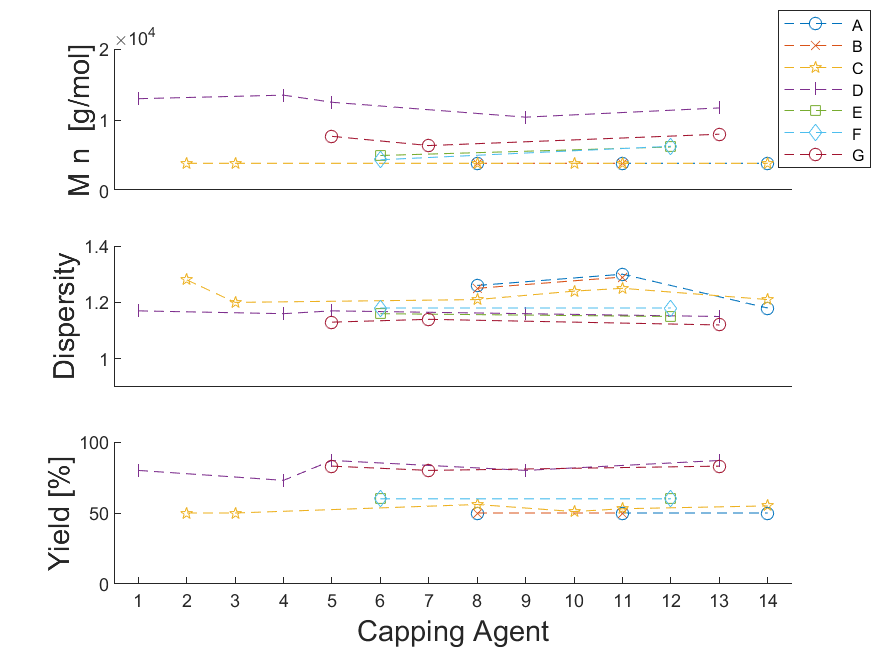

Supplement: Supplementary file 1 [file lg5c00121_si_001.zip › 20_series_Capping Agent.png]

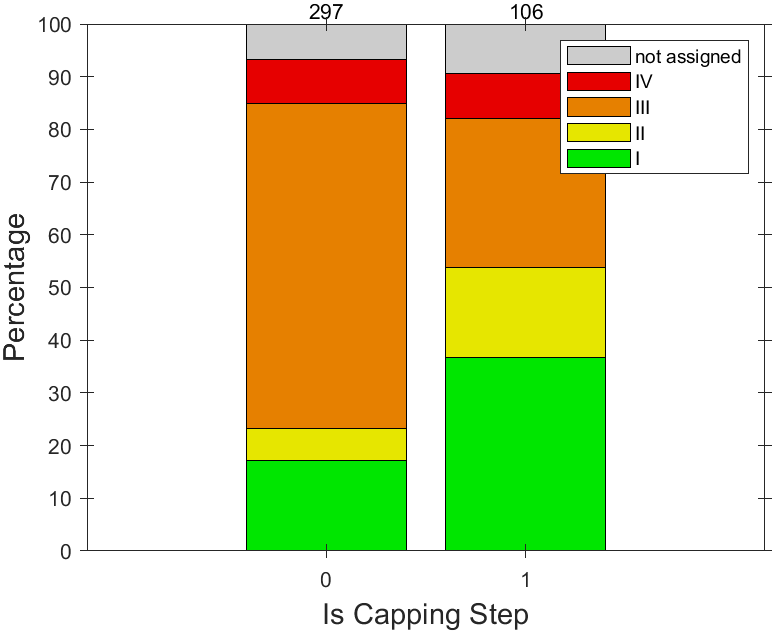

Supplement: Supplementary file 1 [file lg5c00121_si_001.zip › 21_categorization_Is Capping Step.png]

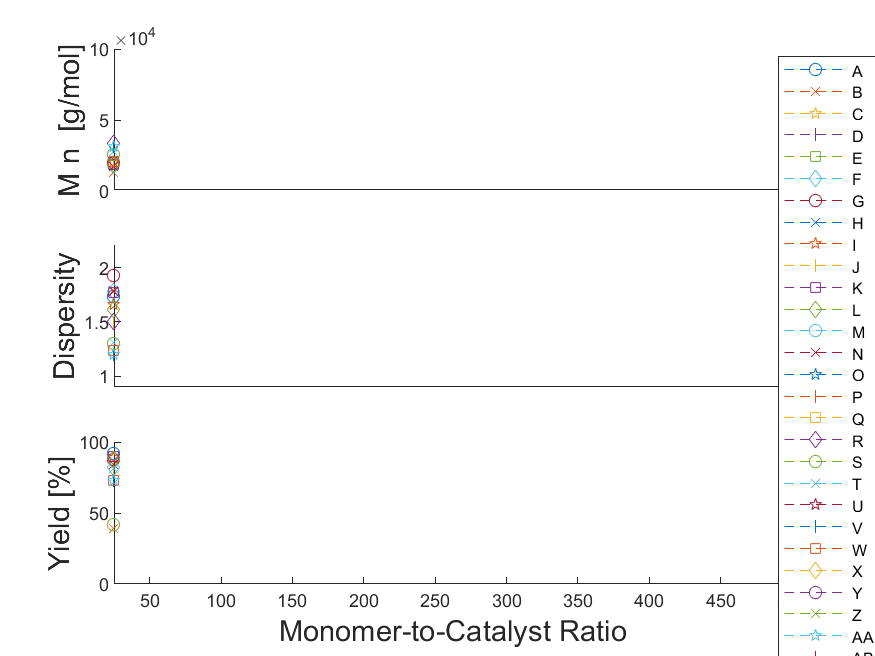

Supplement: Supplementary file 1 [file lg5c00121_si_001.zip › 21_series_Monomer-to-Catalyst Ratio.png]

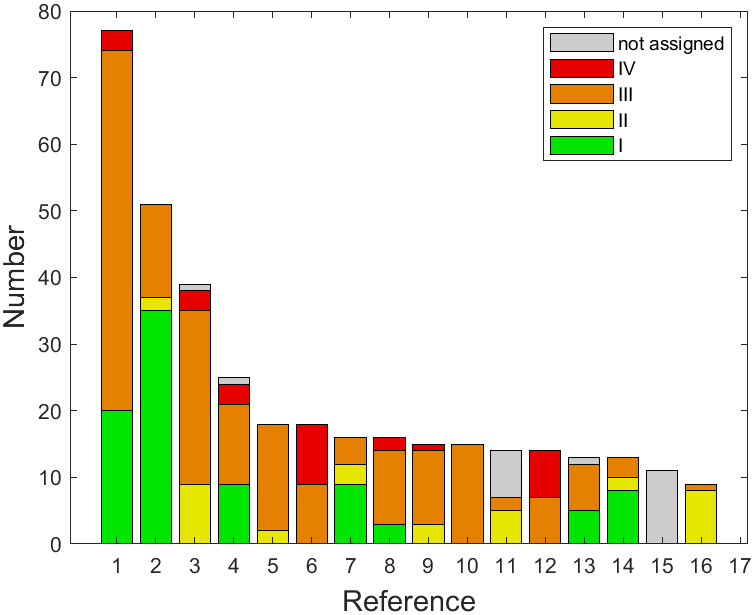

Supplement: Supplementary file 1 [file lg5c00121_si_001.zip › 22_categorization_Reference.png]

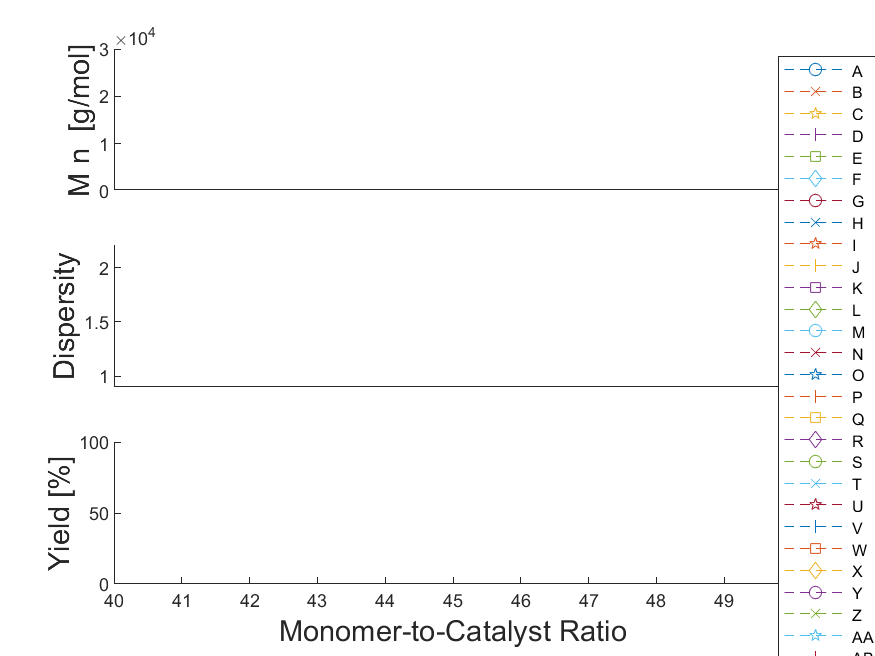

Supplement: Supplementary file 1 [file lg5c00121_si_001.zip › 22_series_Monomer-to-Catalyst Ratio.png]

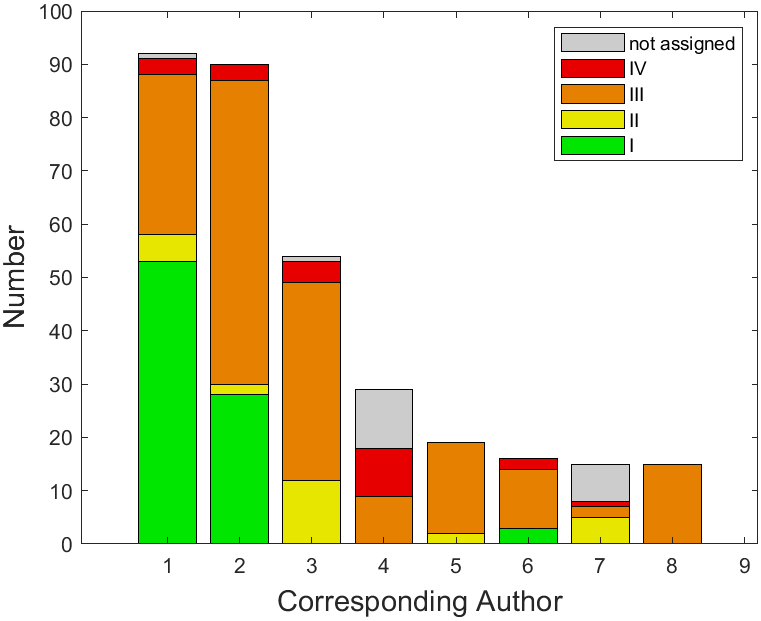

Supplement: Supplementary file 1 [file lg5c00121_si_001.zip › 23_categorization_Corresponding Author.png]

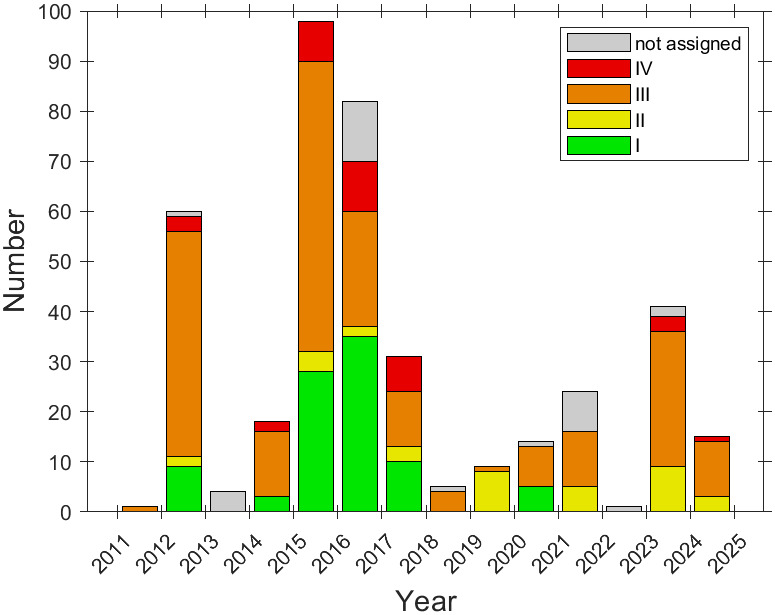

Supplement: Supplementary file 1 [file lg5c00121_si_001.zip › 24_categorization_Year.png]

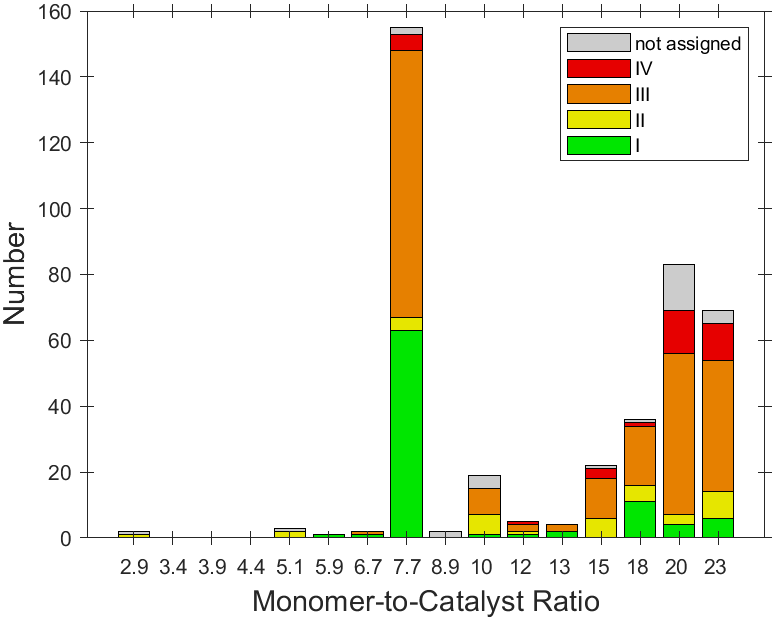

Supplement: Supplementary file 1 [file lg5c00121_si_001.zip › 25_categorization_Monomer-to-Catalyst Ratio.png]

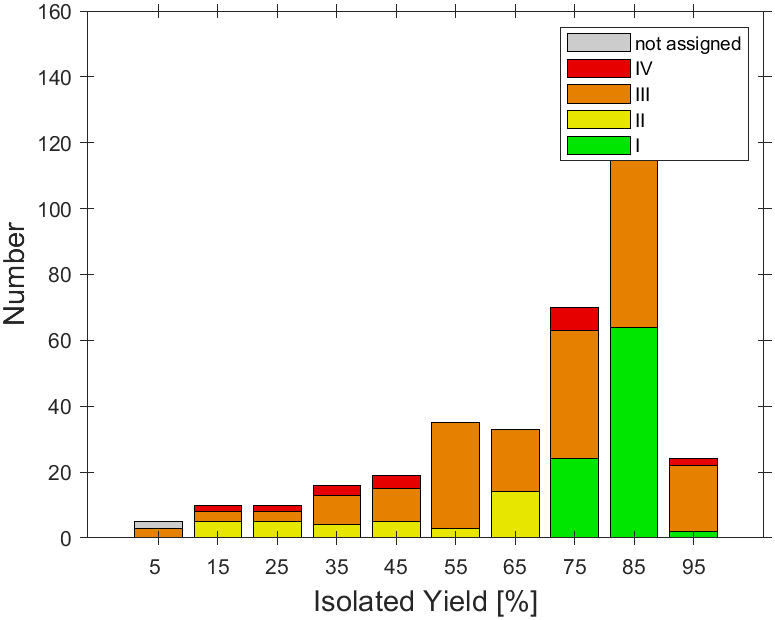

Supplement: Supplementary file 1 [file lg5c00121_si_001.zip › 26_categorization_Isolated Yield.png]

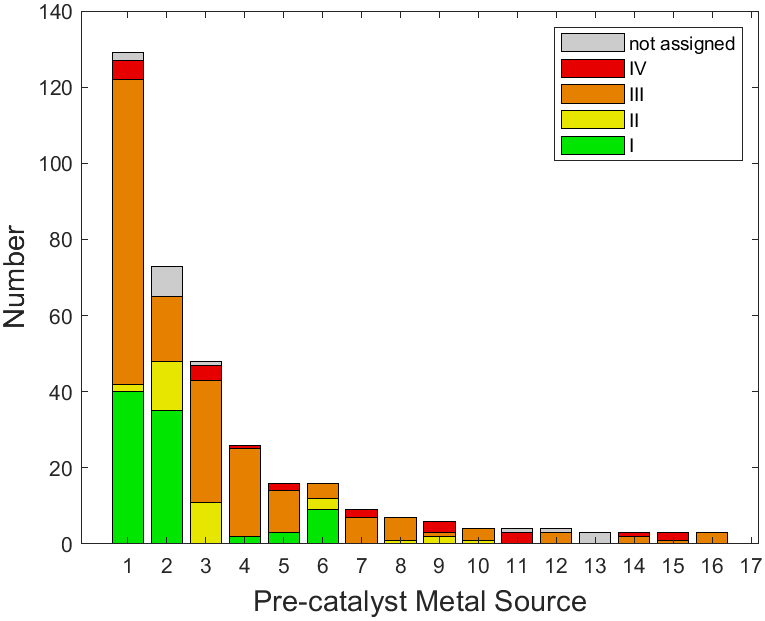

Supplement: Supplementary file 1 [file lg5c00121_si_001.zip › 01_categorization_Pre-catalyst Metal Source.png]

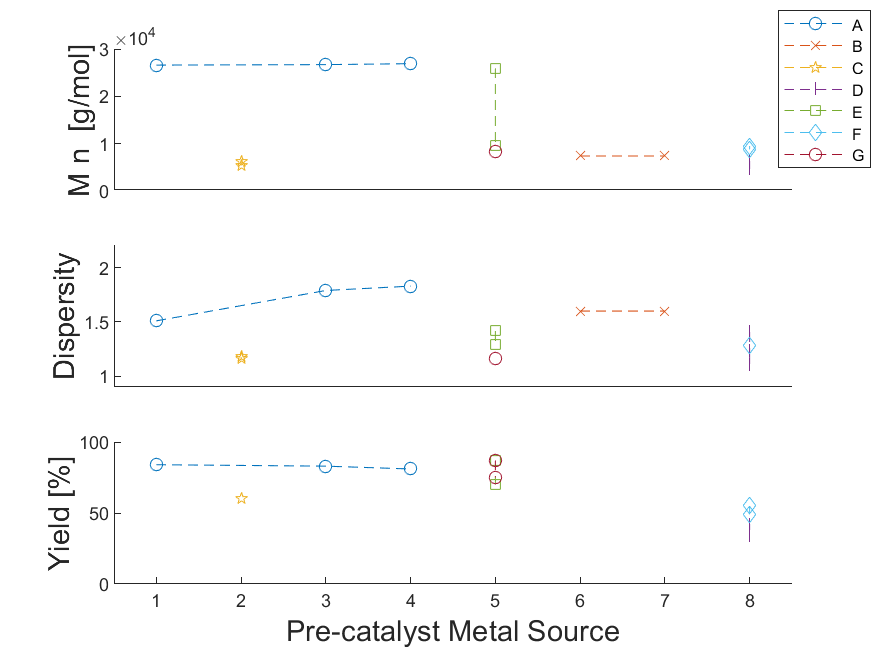

Supplement: Supplementary file 1 [file lg5c00121_si_001.zip › 01_series_Pre-catalyst Metal Source.png]

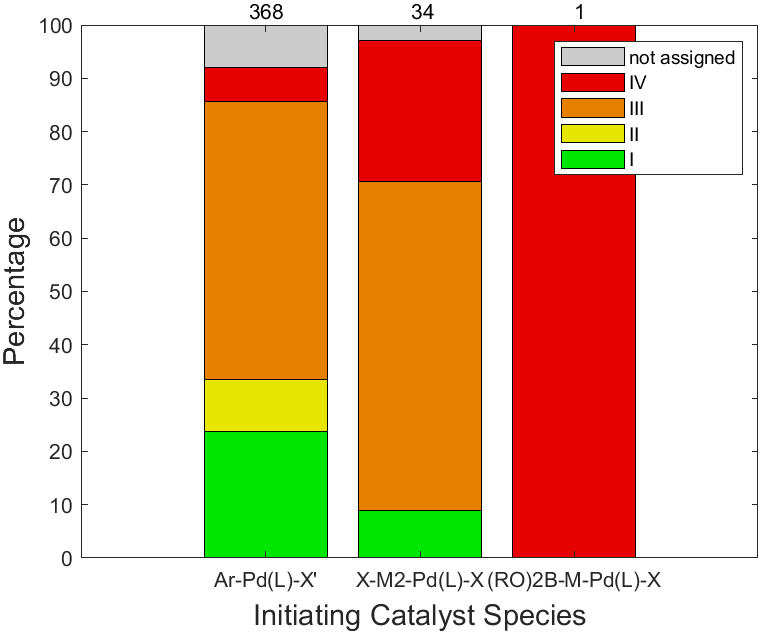

Supplement: Supplementary file 1 [file lg5c00121_si_001.zip › 02_categorization_Initiating Catalyst Species.png]

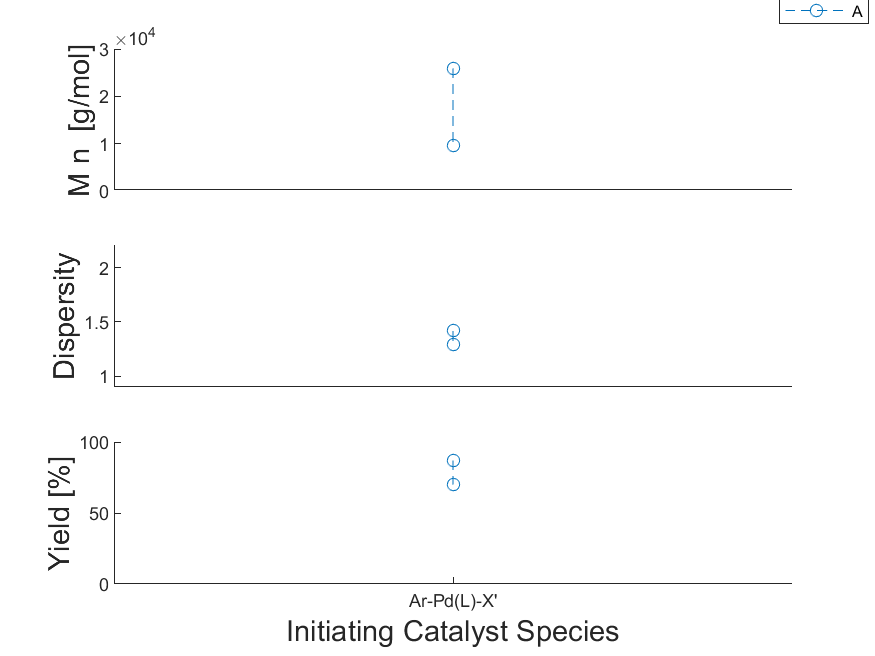

Supplement: Supplementary file 1 [file lg5c00121_si_001.zip › 02_series_Initiating Catalyst Species.png]
