# Supplementary material for: Global Analysis of Polyfluorene via AB-Type Suzuki–Miyaura Polymerization: Empirical and Mechanistic Rationalization of Structural and Reaction Parameters on Molar Mass, Dispersity, and Yield
Source: ACS Polym Au. 2025 Dec 22;6(1):6–32. doi: 10.1021/acspolymersau.5c00121 (PMC12903433; doi:10.1021/acspolymersau.5c00121)
Supplement: Supplementary file 2 [file lg5c00121_si_002.pdf]

Supporting Information

Global analysis of polyfluorene *via* AB-type Suzuki–Miyaura polymerization: Empirical and mechanistical rationalization of structural and reaction parameters on molar mass, dispersity and yield

Alexander Kleine[a,†], *Ulrich S. Schubert*[a,b,*], *Michael Jäger*[a,b,*]

(a) Institute of Organic and Macromolecular Chemistry (IOMC), Friedrich Schiller University Jena, 07743 Jena, Germany

(b) Center for Energy and Environmental Chemistry Jena (CEEC Jena), Friedrich Schiller University Jena, 07743 Jena, Germany

†PRESENT ADDRESS: Fraunhofer Institute for Applied Polymer Research IAP, Research Division Polymeric Materials and Composites PYCO, Schmiedestraße 5, 15745 Wildau, Germany

Table of content

1 Essentials of Suzuki-Miyaura cross-coupling towards polymerization 3

1.1 General Suzuki–Miyaura cross-coupling mechanism 3

1.2 Polymer initiation 4

1.3 Chain-growth behavior 5

1.4 Step-growth behavior 6

1.5 Capping reactions 7

2 “Application-oriented” experiments 8

3 Reference 11

1 Essentials of Suzuki-Miyaura cross-coupling towards polymerization

The following section explains the commonly accepted fundamental aspects of the Pd-mediated Suzuki-Miyaura cross coupling and its implications for polymerization of the corresponding bifunctional monomers (AB-type).

1.1 General Suzuki–Miyaura cross-coupling mechanism

The Suzuki–Miyaura cross-coupling mechanism is explored in great depth,[1, 2] and the most important aspects will be briefly re-called (Scheme 1). The reaction is typically performed in an aqueous/organic solvent mixture with inorganic bases and a Pd-catalyst systems. Once an active Pd^0 catalyst bearing free coordination sites forms, the oxidative addition (OA) of an aryl halide (Ar–X) can occur to form an Ar–Pd^{II}–X complex which undergoes a transmetalation (TM) step with an aryl boron derivative (Ar'–$B(OR)_2$) to yield Ar–Pd^{II}–Ar'. It has been shown that an activation of the Pd center or the boron site is necessary, typically by *in situ* formed hydroxide ions (OH^-). The catalytic cycle is closed by reductive elimination (RE) to release the Ar–Ar' cross-coupling product and re-provide an active catalyst form. Notably, most of the AB-type polymerizations are initiated by a mono-functional aryl halide in order to maintain control of the α-terminus (*vide infra*).

Scheme 1. Simplified schematic representation of the Suzuki–Miyaura cross-coupling.[a]

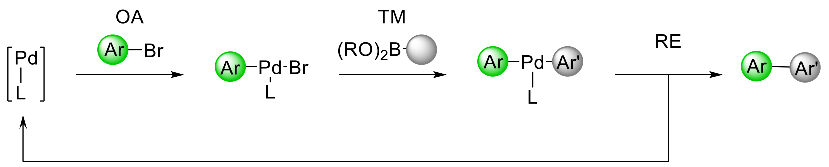

[a]The cross-coupling starts from a Pd^0 catalytic species undergoing oxidative addition (OA), transmetalation (TM) and reductive elimination (RE) releasing the C—C-coupled product and recovering the initial Pd^0 catalytic species. L is ligand(s), $B(OR)_2$ is boronic acid (ester), gray and green circles are aromatic moieties. In the case of the Suzuki–Miyaura polymerization, bifunctional substrates are used to form the polymer chains.

1.2 Polymer initiation

Historically, Pd^{II} salts can be activated for the Suzuki–Miyaura cross-coupling *in situ* by the sacrificial reductive homocoupling of two equivalents of a boronic acid derivative, leading to the release of active Pd^0. Notably, this process can regenerate Pd^{II} that may form due to side reactions in case of (residual) oxygen in the reaction mixture. In the case of small molecule syntheses, the formed homo-coupled product is conveniently removed upon purification. However, in case of the Suzuki–Miyaura polymerization, the homocoupling leads ultimately to AA-terminated polymers (Scheme 2a), which precludes this methodology in view of the desired AB-end group fidelity – despite the possibility for bidirectional growth of polymer chains.

Instead, a Pd^0 precursor (*e.g.* $Pd(dba)_2$) can be used, or a more stable Buchwald-type Pd^{II} palladacycle (Scheme 2b) prone to reductive elimination to release Pd^0 (*e.g.,* upon elimination of carbazole). The Buchwald-type precatalysts benefit from the absence of competing ligands (such as dba), which may compete with the desired oxidative addition and/or transmetalation, *e.g.,* forming dormant species by blocking the free coordination sites. In addition, the initiation is often performed *in situ* prior to polymerization using a monofunctional aryl halide. More recently, the progress towards stable and isolable Pd complexes also enables their direct use as exemplified for systematic screening studies.[3]

Scheme 2. Schematic representation for the formation of the initiating catalyst system from Pd^{II}- or Pd^0-based pre-catalysts.[a]

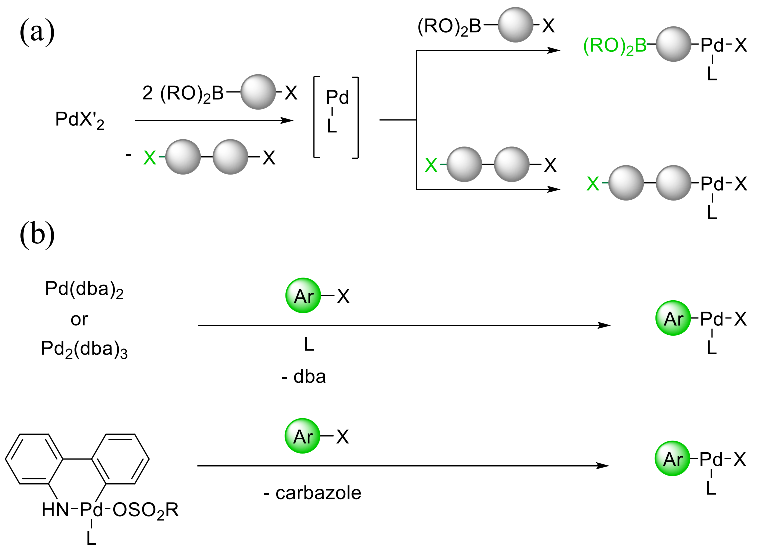

[a]*(a) For Pd^{II}-based precatalysts, activation by reductive coupling leads to a Pd^{0}-active catalyst and dimerized monomer ($X{-}Ar_2{-}X$), which is converted with monomer (top path) to correct α-end group $B(OR)_2$ or the formal incorrect α-end group (X) (bottom path). (b) Conversion with external mono-aryl halide to selectively introduce an α-unit Ar (green sphere). Green highlights the resulting α-end group introduced by the respective activation step. L is ligand, $B(OR)_2$ is boronic acid (ester), X is (pseudo)halide.*

1.3 Chain-growth behavior

In the chain-growth mechanism, the catalyst remains on the polymer chain and the monomer units are successively incorporated. Hence, the reductive elimination step should be followed by the migration of the Pd catalyst fragment *via* weak bonding to the π-system towards the new chain terminus (Scheme 3). As a consequence of this "ring walking" (RW) step,[4] the oxidative addition on the same polymer chain can occur if AB-type monomers are utilized. In essence, the boron derivative group (B) serves for the coupling of the monomer to the polymer chain and the (pseudo)halide (A) forms the new reactive chain end, amenable for the next coupling step. If the polymerization reaction follows this mechanism, a true Suzuki–Miyaura catalyst-transfer polymerization (SCTP) is ensured. A monofunctional initiator (Ar–X) is often used to initiate the polymerization at the α-terminus to prevent chain-chain couplings (*vide infra*). Hence, the monomer/palladium ratio and the monomer conversion can theoretically be utilized to tune the degree of polymerization (DP) as well as to obtain narrow dispersity values ($Đ \leq 1.20$) and a high end group fidelity. Notably, the same polymerization characteristics in

terms of DP and Đ is expected if the catalyst dissociates from the chain but ultimately undergoes oxidative addition to a polymer chain's ω-terminus. Hence, in the ideal SCTP case, the interaction of the Pd^0 with the π-system is of crucial importance, while in the latter case additional contributions with the solvent and/or additives may contribute (*vide infra*).

Scheme 3. Schematic representation of the SCTP behavior after the reductive elimination (RE).[a]

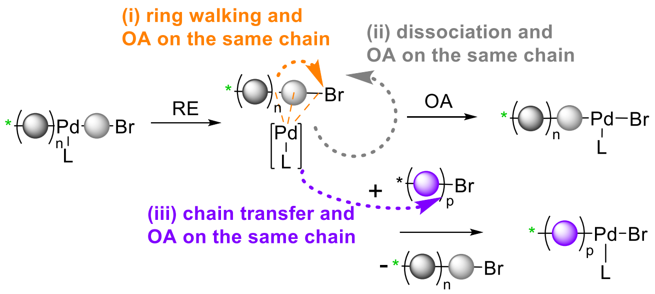

[a]*(i) In case of strict ring walking (RW, in orange) and oxidative addition (OA) at the same chain, (ii) catalyst dissociation but oxidative addition (OA) on the same polymer chain (gray), or (iii) catalyst dissociation and oxidative addition to a different polymer chain (purple) leading to chain transfer. L is ligand. n and p denote different polymer chains. Asterisk denotes α-terminus, typically a monofunctional aryl unit.*

1.4 Step-growth behavior

In contrast to the chain-growth behavior (*vide supra*), the active catalyst fragment reacts with a monomer. If so, new chains are initiated (Scheme 4) that may grow accordingly with the SCTP behavior. Importantly, these chains bear a boron-derivative at the α-terminus, which opens the possibility of coupling between two polymer chains. Consequently, larger DPs can be expected in line with a higher dispersity of up to Đ = 2.0. In addition, the chain coupling results in the release of the active catalyst fragment, so that further new chains can be initiated. In the limit of quantitative chain coupling, the resulting polymer chains should bear only the initial α-end group (typically an aryl unit), while upon intermediate conversion sizable amounts of polymer with boronic acid derivative at the α-terminus are expected. In essence, the conversion as well as the extent of chain-growth *vs.* step-growth will affect the observed degree

of polymerization and dispersity. A high isolated polymer yield would indicate efficient couplings, so that the combination of an unexpectedly high degree of polymerization and high dispersity suggests a sizable contribution of step-growth (*vide infra*).

Scheme 4. Schematic representation of side reactions followed by catalyst dissociation.[a]

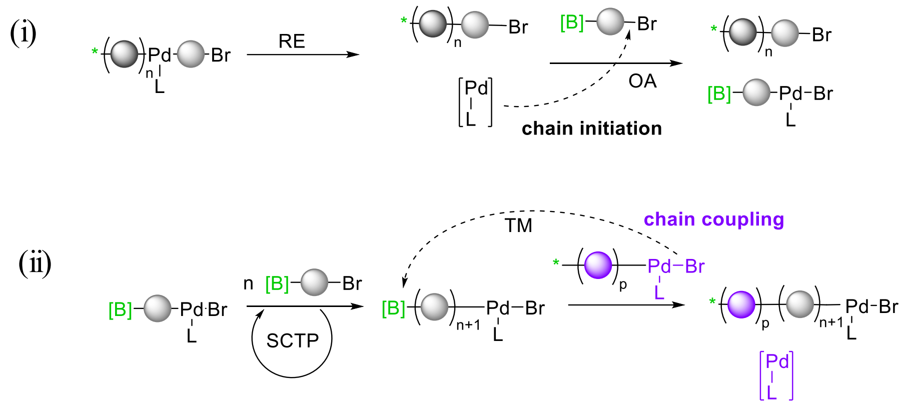

a. (i) Initiation of a new chain upon OA with a monomer, and (ii) SCTP preserving the boronic acid α-terminus (green) capable of subsequent chain couplings (purple) with other active chains, resulting in the release of an active catalyst fragment. L is ligand, [B] is boronic acid derivative.

1.5 Capping reactions

Assuming catalyst dissociation may occur, the presence of surplus monoaryl (pseudo)halide (Ar–X) should lead – after oxidative addition with the active Pd–L catalyst – to the correctly initiated α-terminus *via* Ar–Pd(L)–X as in the initiation stage (*vide supra*). In addition, the Ar–Pd(L)–X is capable to convert the boronic acid α-terminus to the correct end group (Scheme 4, step ii, with p = 0). In most cases, polymerization is quenched by the addition of acid (*e.g.*, aqueous HCl). As a result, the active chain ends are protonated and the corresponding Pd^{II} salts are released. In the case of boronic acid termini, protodeboronation with HCl can occur. Alternatively, a monofunctional boronic acid derivative can be added to cap the ω-terminus yielding heterotelechelic polymers, or a second polymerization process can be started *in situ* to form block copolymers.

With the categorization in hand, this section provides a first overview of studies on poly(fluorene)s. The first part covers reports that focus on the properties and applications of the synthesized polymers and is kept concise as only few polymerization reactions were performed. The second part provides an overview of the studies that explored systematically selected parameters on the polymerization results.

2 "Application-oriented" experiments

This section summarizes the work towards the usage and incorporation of synthesized poly(fluorene) into more complex architectures, *e.g.*, in polymer analogous reactions, as macroinitiators or onto graft polymers. Hence, the performed polymerization reactions were typically not analyzed in depth and yielded mostly moderately broad distributions, as indicated by the available data for categorization (I to IV). Some results indicate the proposed SCTP mechanism while others are assigned to a step-growth polymerization based on the analytical SEC data.

Table 1. Application-oriented studies of poly(fluorene)s via AB-type Suzuki polycondensation.

Entry	1st author (year)	# exp.	M_n [kg/mol] (Đ≤1.2, yield≥70%)	M_n [kg/mol] (Đ≤1.2, yield<70%)	M_n [kg/mol] (1.2<Đ<1.7)	M_n [kg/mol] (Đ>=1.7)	M_n^{max} [kg/mol]	Đ for M_n^{max}	Ref
**1**	Elmalem (2011)	1	-	-	9.6	-	9.6	1.41	5
**2**	Elmalem (2012)	18	-	-	3.9[a]	-	3.9[a]	1.18	6
**3**	Pevzner (2014)	3	-	7.6	20.4 to 28.7	-	28.7	1.35	7
**4**	de Roo (2014)	2	-	4.0[b]	-	-	4. 0[b]	1.20[b]	8
**5**	de Roo (2016)	1	-	-	-	15.8[b]	15.8[b]	2.70[b]	9
**6**	Fischer (2013)	4	-	6.8 to 9.9	15.0	-	15.0	1.25	10
**7**	Zhou (2018)	1	-	5.4[b]	-	-	5.4[b]	1.15[b]	11
**8**	Gutacker (2012)	2	-	-	8.1 to 10.0	-	10.0	1.27	12
**9**	Steverlynck (2016)	1	-	-	-	11.8	11.8	1.80	13
**10**	Tsuji (2017)	1	9.0	-	-	-	9.0	1.20	14
**11**	Jiang (2020)	1	-	-	9.4	-	9.4	1.38	15

**12**	Jiang (2021)	10	-	-	11.0 to 28.7	-	28.7	1.50	16
**13**	Tokita (2021)	1	.	-	4.6[b]	-	4.6	1.46	17
**14**	Fujiki (2022)	1	.	1.1[b]	.	.	1.54[c]	<1.10[c]	18
**15**	Zou (2023)	1	-	-	10.1	-	10.1	1.30	19
**16**	He (2023)	1	-	-	3.8	-	3.8	1.60	20
**17**	Nakamura (2023)	1	-	-	27.7	-	27.7	1.61	21

a. M_n from averaged data by NMR spectroscopy. b. No yield was stated. c. Calculated from MALDI-MS data from Fig. S11 in reference[18].

Elmalem *et al.* (2011) synthesized a poly(fluorene) to compare the polymer to a push-pull-type polymer based on fluorene-2,7-diyl as the donor and benzothiadiazole as the acceptor unit (*Table 1*, entry 1).[5] Next, both Elmalem *et al.* (2012) and Pevzner *et al.* investigated the properties of the synthesized polymers (*Table 1*, entries 2 to 3).[6, 7] Furthermore, Elmalem *et al.* (2012) synthesized heterotelechelic polymers with α- and ω-chain ends with varying electronic characteristics. The influence of the chosen aryl halide and end-functionalization reagents on the charge- and energy-transfer properties was investigated. Pevzner *et al.* used different fluorinated side chains on the fluorene chromophore, examining the obtained opto-electronic properties. The Mecking group performed syntheses of poly(fluorene)s on the surface of cadmium-based quantum dots yielding hybrid particles, using a *grafting to* approach or a *grafting from* approach (*Table 1*, entries 4 and 5).[8, 9] Various reports detail the synthesis of *block* copolymers. Fischer *et al.* synthesized *block* copolymers with polyethylene glycol in a *grafting to* approach to form nanoparticles (*Table 1*, entry 6),[10] while Zhou *et al.* have chosen a *grafting from* approach with 2-(dimethylamino)ethyl methacrylate to form micellular structures (*Table 1*, entry 7).[11] Gutacker *et al.* investigated copolymers in a *grafting from* approach (*Table 1*, entry 8) but used fluorene-based monomers in both blocks that vary only in their side chains. By post-polymerization modification, a neutral-*block*-ionic copolymer was synthesized and analyzed according to its aggregation behavior.[12] Steverlynck *et al.* synthesized *graft*-copolymers with poly(fluorene) in the side chain and poly(thiophene) in the main chain in a

grafting to approach (*Table 1*, entry 9).[13] In a similar approach, Tsuji *et al.* grafted poly(fluorene) to a xanthene-based polymer backbone forming self-assembling polymer-layered structures (*Table 1*, entry 10).[14] Jiang *et al.* (2020) (*Table 1*, entry 11) used poly(fluorene)s for luminescent nanofibers by blending conjugated rod-coil block copolymers with an inorganic perovskite-based sensitizer.[15] Later, Jiang *et al.* (2021) extended the concept to rod-coil block copolymers with bio-based materials to explore stretchable touch-responsive light emitting diodes (*Table 1*, entry 12).[16] Tokita *et al.* (2021) reported a single polymerization that was further exploited in the study (*Table 1*, entry 13).[17] In 2022, Fujiki *et al.* applied the SCTP methodology to hybridized poly(fluorene)s supported by a dendrimer followed by reductive cleavage (*Table 1*, entry 14).[18] Zou *et al.* (2023) prepared helical assemblies featuring circularly polarized luminescence by combining a poly(fluorene)-based block with poly(phenyl isocyanide)s (*Table 1*, entry 15).[19] He *et al.* (2023) functionalized paper with poly(fluorene)s as fluorescent sensors for screening of cell carcinoma (*Table 1*, entry 16).[20] Nakamura *et al.* (2023) reported on single-chain force microscopy and fluorescence spectroscopy to elucidate intra-chain aggregation coupling phenomena (*Table 1*, entry 17).[21] The latter example of block and grafted copolymers that elegantly demonstrate the continuous high interest to use poly(fluorene)s as light- and electro-responsive materials.

Table 2. Overview of optimization studies on AB-type Suzuki polycondensation reactions.[a]

Entry	1st author (year)	# exp.	M_n [kg/mol][b] (Đ≤1.2, yield≥70%)	M_n [kg/mol][b] (Đ≤1.2, yield<70%)	M_n [kg/mol][b] (1.2<Đ<1.7)	M_n [kg/mol][b] (Đ>=1.7)	M_n^{max} [kg/mol]	Đ for M_n^{max}	Ref
**1**	Sui (2014)	20	-	-	10.5 to 70.4 (9)	56.4 to 77.4 (3)	77.4	1.78	22
**2**	Grisorio (2014)	16	14.0 to 27.0 (3)	-	9.9 to 31.7 (11)	20.9 to 21.3 (2)	31.7	1.41	23
**3**	Grisorio (2017)	14	-	-	7.4 to 30.3 (7)	5.0 to 26.9 (7)	30.3	1.24	24
**4**	Zhang (2020)	8	34.8 (1)	-	9.2 to 42.3 (2)	4.9 to 76.4 (4)	76.4	2.00	25
**5**	Ying (2012)	16	-	-	8.1 to 17.7 (15)	24.8 (1)	24.8	1.73	26
**6**	Kosaka (2018)	4	-	-	6.2 to 15.3 (4)	-	15.3	1.43	27
**7**	Zhang (2015)	11	-	6.9 to 8.7 (2)	9.4 (1)	8.9 to 25.7 (8)	25.7	1.74	28
**8**	Sun (2016)	27	-	-	14.7 to 22.0 (12)	12.5 to 20.1 (12)	22.0	1.67	29

**9**	Zhang (2016)	11	-	-	7.4[c]	-	7.4[c]	1.60[c]	30
**10**	Zhang (2012)	25	9.1 to 31.4 (9)	-	4.7 to 25.9 (12)	2.5 to 47.3 (3)	47.3	1.95	31
**11**	Zhang (2015)	13	9.6 to 13.5 (8)	19.4 to 30.0 (2)	6.9 to 9.2 (3)		30.0	1.19	32
**12**	Zhang (2015)	77	6.3 to 31.4 (20)	-	4.7 to 43.1 (54)	2.5 to 29.9 (3)	43.1	1.67	33
**13**	Zhang (2016)	52	6.0 to 17.3 (35)	6.3 to 7.7 (2)	5.3 to 33.4 (15)	-	33.4	1.23	34
**14**	Dong (2017)	17	3.4 to 11.4 (9)	2.5 to 9.4 (4)	7.6 to 10.7 (4)	-	11.4	1.18	35
**15**	Huber (2019)	9	-	2.3 to 11.5 (8)	17.0 (0)	-	17.0	1.63	36
**16**	Kobayashi (2020)	28	4.8 to 21.3 (10)	-	4.8 to 69.4 (17)	-	69.4	1.38	37
**17**	Delabie (2021)	16	-	3.7 to 17.1 (5)	24.5 to 27.4 (3)	-	36.4	1.60	38
**18**	Howell (2023)	56	-	3.5 to 6.2 (11)	3.2 to 13.6 (32)	7.8 to 18.6 (10)	18.6	1.96	39
**19**	Howell (2024)	19	-	4.4 to 5.7 (3)	4.1 to 12.0 (12)	8.2 to 12.7 (4)	12.7	1.98	3

[a]Blue shaded: Mainly Pd^{II} precatalysts. Color shadings of M_n indicate the categorization of results (see text for details). [b]Number in parentheses represents the number of experiments per category. Note that category "non-assigned" is omitted for brevity. [c]Incomplete data (yields reported in the range from 81 to 88%, molar mass from MALDI analysis).

3 Reference

1. D'Alterio, M. C.; Casals-Cruañas, E.; Tzouras, N. V.; Talarico, G.; Nolan, S. P.; Poater, A., Mechanistic Aspects of the Palladium-Catalyzed Suzuki-Miyaura Cross-Coupling Reaction. *Chem. Eur. J.* **2021,** *27* (54), 13481-13493.

2. Escayola, S.; Bahri-Laleh, N.; Poater, A., %<i>V</i>_{Bur} index and steric maps: from predictive catalysis to machine learning. *Chem. Soc. Rev.* **2024,** *53* (2), 853-882.

3. Howell, M. T.; Fronczek, F. R.; Nesterov, E. E., MIDA Boronate Monomers in Suzuki-Miyaura Catalyst-Transfer Polymerization: The Pros and Cons. *Macromolecules* **2024**.

4. Leone, A. K.; Goldberg, P. K.; McNeil, A. J., Ring-Walking in Catalyst-Transfer Polymerization. *J. Am. Chem. Soc.* **2018,** *140* (25), 7846-7850.

5. Elmalem, E.; Kiriy, A.; Huck, W. T. S., Chain-Growth Suzuki Polymerization of n-Type Fluorene Copolymers. *Macromolecules* **2011,** *44* (22), 9057-9061.

6. Elmalem, E.; Biedermann, F.; Johnson, K.; Friend, R. H.; Huck, W. T., Synthesis and photophysics of fully pi-conjugated heterobis-functionalized polymeric molecular wires via Suzuki chain-growth polymerization. *J. Am. Chem. Soc.* **2012,** *134* (42), 17769-77.

7. Pevzner, L.; Auer, M.; Trattnig, R.; Klapper, M.; List-Kratochvil, E. J. W.; Mullen, K., Controlling Polymer Solubility: Polyfluorenes with Branched Semiperfluorinated Side Chains for Polymer Light-Emitting Diodes. *Isr. J. Chem.* **2014,** *54* (5-6), 736-747.

8. de Roo, T.; Haase, J.; Keller, J.; Hinz, C.; Schmid, M.; Seletskiy, D. V.; Cölfen, H.; Leitenstorfer, A.; Mecking, S., A Direct Approach to Organic/Inorganic Semiconductor Hybrid Particles via Functionalized Polyfluorene Ligands. *Adv. Funct. Mater.* **2014,** *24* (18), 2714-2719.

9. de Roo, T.; Huber, S.; Mecking, S., CdSe/CdS—Conjugated Polymer Core–Shell Hybrid Nanoparticles by a Grafting-From Approach. *ACS Macro Lett.* **2016,** *5* (7), 786-789.

10. Fischer, C. S.; Baier, M. C.; Mecking, S., Enhanced brightness emission-tuned nanoparticles from heterodifunctional polyfluorene building blocks. *J. Am. Chem. Soc.* **2013,** *135* (3), 1148-54.

11. Zhou, M.; Li, J.; Zhang, H. H.; Hong, K. L., Stimuli-responsive fiber-like micelles from the self-assembly of well-defined rod-coil block copolymer. *Eur. Polym. J.* **2018,** *103*, 304-311.

12. Gutacker, A.; Lin, C. Y.; Ying, L.; Nguyen, T. Q.; Scherf, U.; Bazan, G. C., Cationic Polyfluorene-b-Neutral Polyfluorene "Rod-Rod" Diblock Copolymers. *Macromolecules* **2012,** *45* (11), 4441-4446.

13. Steverlynck, J.; De Cattelle, A.; De Winter, J.; Gerbaux, P.; Koeckelberghs, G., Energy Transfer in Poly(3-hexylthiophene)-g-Polyfluorene Graft Copolymers. *J. Polym. Sci., Part A: Polym. Chem.* **2016,** *54* (9), 1252-1258.

14. Tsuji, Y.; Morisaki, Y.; Chujo, Y., pi-Conjugated polymer-layered structures: synthesis and self-assembly. *Polym. J.* **2017,** *49* (1), 203-208.

15. Jiang, D. H.; Kobayashi, S.; Jao, C. C.; Mato, Y.; Isono, T.; Fang, Y. H.; Lin, C. C.; Satoh, T.; Tung, S. H.; Kuo, C. C., Light Down-Converter Based on Luminescent Nanofibers from the Blending of Conjugated Rod-Coil Block Copolymers and Perovskite through Electrospinning. *Polymers* **2020,** *12* (1).

16. Jiang, D. H.; Ree, B. J.; Isono, T.; Xia, X. C.; Hsu, L. C.; Kobayashi, S.; Ngoi, K. H.; Chen, W. C.; Jao, C. C.; Veeramuthu, L.; Satoh, T.; Tung, S. H.; Kuo, C. C., Facile one-pot synthesis of rod-coil bio-block copolymers and uncovering their role in forming the efficient stretchable touch-responsive light emitting diodes. *Chem. Eng. J.* **2021,** *418*.

17. Tokita, Y.; Katoh, M.; Kosaka, K.; Ohta, Y.; Yokozawa, T., Precision synthesis of a fluorene-thiophene alternating copolymer by means of the Suzuki-Miyaura catalyst-transfer condensation polymerization: the importance of the position of an alkyl substituent on thiophene of the biaryl monomer to suppress disproportionation. *Polym. Chem.* **2021,** *12* (48), 7065-7072.

18. Fujiki, S. S.; Amaike, K.; Yagi, A.; Itami, K., Synthesis, properties, and material hybridization of bare aromatic polymers enabled by dendrimer support. *Nat. Commun.* **2022,** *13* (1).

19. Zou, H.; Liu, W.; Wang, C.; Zhou, L.; Liu, N.; Wu, Z. Q., Polyfluorene-block-poly(phenyl isocyanide) Copolymers: One-Pot Synthesis, Helical Assembly, and Circularly Polarized Luminescence. *Macromolecules* **2023,** *56* (5), 1875-1883.

20. He, Z. J.; Huang, J. Y.; Shen, W. Y.; Lei, X. Y.; Zhang, Y. F.; Zhu, L. L.; Shen, X. Y.; Zhang, D.; Yu, D.; Zhou, M., A Paper-Based Fluorescent Sensor for Rapid Early Screening of Oral Squamous Cell Carcinoma. *ACS Appl. Mater. Interfaces* **2023,** *15* (20), 24913-24922.

21. Nakamura, T.; Omagari, S.; Liang, X. B.; Tan, Q. W.; Nakajima, K.; Vacha, M., Simultaneous Force and Fluorescence Spectroscopy on Single Chains of Polyfluorene: Effect of Intra-Chain Aggregate Coupling. *ACS Nano* **2023,** *17* (9), 8074-8082.

22. Sui, A. G.; Shi, X. C.; Tian, H. K.; Geng, Y. H.; Wang, F. S., Suzuki-Miyaura catalyst-transfer polycondensation with Pd(IPr)(OAc)(2) as the catalyst for the controlled synthesis of polyfluorenes and polythiophenes. *Polym. Chem.* **2014,** *5* (24), 7072-7080.

23. Grisorio, R.; Mastrorilli, P.; Suranna, G. P., A Pd(AcO)(2)/t-Bu3P/K3PO4 catalytic system for the control of Suzuki cross-coupling polymerisation. *Polym. Chem.* **2014,** *5* (14), 4304-4310.

24. Grisorio, R.; Suranna, G. P., Impact of Precatalyst Activation on Suzuki-Miyaura Catalyst-Transfer Polymerizations: New Mechanistic Scenarios for Pre-transmetalation Events. *ACS Macro Lett.* **2017,** *6* (11), 1251-1256.

25. Zhang, K.; Tkachov, R.; Ditte, K.; Kiriy, N.; Kiriy, A.; Voit, B., AB- Versus AA+BB-Suzuki Polycondensation: A Palladium/Tris(tert-butyl)phosphine Catalyst Can Outperform Conventional Catalysts. *Macromol. Rapid Commun.* **2020,** *41* (1), e1900521.

26. Ying, L.; Zalar, P.; Collins, S. D.; Chen, Z.; Mikhailovsky, A. A.; Nguyen, T. Q.; Bazan, G. C., All-conjugated triblock polyelectrolytes. *Adv. Mater.* **2012,** *24* (48), 6496-501.

27. Kosaka, K.; Uchida, T.; Mikami, K.; Ohta, Y.; Yokozawa, T., AmPhos Pd-Catalyzed Suzuki Miyaura Catalyst-Transfer Condensation Polymerization: Narrower Dispersity by Mixing the Catalyst and Base Prior to Polymerization. *Macromolecules* **2018,** *51* (2), 364-369.

28. Zhang, Z. L.; Hu, P.; Li, X.; Zhan, H. M.; Cheng, Y. X., Investigation of Suzuki-Miyaura Catalyst-Transfer Polycondensation of AB-Type Fluorene Monomer Using Coordination-Saturated Aryl Pd(II) Halide Complexes as Initiators. *J. Polym. Sci., Part A: Polym. Chem.* **2015,** *53* (12), 1457-1463.

29. Sun, H. L.; Zhang, S. R.; Yang, Y. K.; Li, X.; Zhan, H. M.; Cheng, Y. X., Excellent Control of Perylene Diimide End Group in Polyfluorene via Suzuki Catalyst Transfer Polymerization. *Macromolecular Chemistry and Physics* **2016,** *217* (24), 2726-2735.

30. Zhang, S. R.; Zhang, Z. L.; Fu, H. W.; Li, X.; Zhan, H. M.; Cheng, Y. X., Synthesis of polyfluorene containing simple functional end group with aryl palladium(II) complexes as initiators. *J. Organomet. Chem.* **2016,** *825*, 100-113.

31. Zhang, H. H.; Xing, C. H.; Hu, Q. S., Controlled Pd(0)/t-Bu3P-catalyzed Suzuki cross-coupling polymerization of AB-type monomers with PhPd(t-Bu3P)I or Pd2(dba)3/t-Bu3P/ArI as the initiator. *J. Am. Chem. Soc.* **2012,** *134* (32), 13156-9.

32. Zhang, H. H.; Hu, Q. S.; Hong, K., Accessing conjugated polymers with precisely controlled heterobisfunctional chain ends via post-polymerization modification of the OTf group and controlled Pd(0)/t-Bu3P-catalyzed Suzuki cross-coupling polymerization. *Chem Commun (Camb)* **2015,** *51* (80), 14869-72.

33. Zhang, H. H.; Xing, C. H.; Hu, Q. S.; Hong, K. L., 'Controlled Pd(0)/t-Bu3P-Catalyzed Suzuki Cross-Coupling Polymerization of AB-Type Monomers with ArPd(t-Bu3P)X or Pd-2(dba)(3)/t-Bu3P/ArX as the Initiator. *Macromolecules* **2015,** *48* (4), 967-978.

34. Zhang, H. H.; Peng, W.; Dong, J.; Hu, Q. S., t-Bu3P-Coordinated 2-Phenylaniline-Based Palladacycle Complex/ArBr as Robust Initiators for Controlled Pd(0)/t-Bu3P-Catalyzed Suzuki Cross-Coupling Polymerization of AB-Type Monomers. *ACS Macro Lett.* **2016,** *5* (6), 656-660.

35. Dong, J.; Guo, H.; Hu, Q. S., Controlled Pd(0)/Ad(3)P-Catalyzed Suzuki Cross-Coupling Polymerization of AB-Type Monomers with Ad(3)P-Coordinated Acetanilide-Based Palladacycle Complex as Initiator. *ACS Macro Lett.* **2017,** *6* (11), 1301-1304.

36. Huber, S.; Mecking, S., Straightforward Synthesis of Conjugated Block Copolymers by Controlled Suzuki-Miyaura Cross-Coupling Polymerization Combined with ATRP. *Macromolecules* **2019,** *52* (15), 5917-5924.

37. Kobayashi, S.; Fujiwara, K.; Jiang, D. H.; Yamamoto, T.; Tajima, K.; Yamamoto, Y.; Isono, T.; Satoh, T., Suzuki-Miyaura catalyst-transfer polycondensation of triolborate-type fluorene monomer: toward rapid access to polyfluorene-containing block and graft copolymers from various macroinitiators. *Polym. Chem.* **2020,** *11* (42), 6832-6839.

38. Delabie, J.; Ceunen, W.; Detavernier, S.; De Winter, J.; Gerbaux, P.; Verbiest, T.; Koeckelberghs, G., Catechol as a Universal Linker for the Synthesis of Hybrid Polyfluorene/Nanoparticle Materials. *Macromolecules* **2021,** *54* (10), 4582-4591.

39. Howell, M. T.; Kei, P.; Anokhin, M. V.; Losovyj, Y.; Fronczek, F. R.; Nesterov, E. E., Suzuki-Miyaura catalyst-transfer polymerization: new mechanistic insights. *Polym. Chem.* **2023,** *14* (37), 4319-4337.
